# Screening and Molecular Modeling Evaluation of Food Peptides to Inhibit Key Targets of COVID-19 Virus

**DOI:** 10.3390/biom11020330

**Published:** 2021-02-22

**Authors:** Ai-Min Shi, Rui Guo, Qiang Wang, Jin-Rong Zhou

**Affiliations:** 1 Institute of Food Science and Technology, Chinese Academy of Agricultural Sciences/Key Laboratory of Agricultural Product Processing and Quality Control, Ministry of Agricultural and Rural Affairs, Beijing 100193, China; shiaimin@caas.cn (A.-M.S.); guorui923@outlook.com (R.G.); 2 Beth Israel Deaconess Medical Center, Harvard Medical School, Boston, MA 02115, USA; jrzhou@bidmc.harvard.edu

**Keywords:** food peptides, COVID-19 virus, molecular docking, molecular dynamic simulation

## Abstract

Peptide drugs, especially food-derived peptides, have a variety of functional activities including antiviral and may also have a therapeutic effect on COVID-19. In this study, comparing with the reported drugs, 79 peptides were found to bind to the key targets of COVID-19 virus with higher non-covalent interaction, while among them, six peptides showed high non-covalent interactions with the three targets, which may inhibit the COVID-19 virus. In the simulation, peptides of nine to 10 amino acids with a hydrophilic amino acid and acidic amino acid in the middle and aromatic amino acids on the side showed higher binding to angiotensin-converting enzyme 2 (ACE2). Peptides of five to six amino acids with a basic amnio acid in the head, acidic amnio acid in the neck, hydrophobicity group in the middle, and basic amino acids in the tail showed higher binding to COVID-19 virus main protease (M^pro^), while those with basic amino acids and acidic amino acids in the two sides and aromatic amino acids in the middle might have stronger interaction with COVID-19 virus RNA-dependent RNA polymerase (RdRp).

## 1. Introduction

Since December 2019, coronavirus disease 2019 (COVID-19) has been raging for a whole year; over 100 million people were infected, and over 2.2 million people died [1]. Although related to the severe acute respiratory syndrome (SARS) and the Middle East respiratory syndrome (MERS), COVID-19 shows some peculiar pathogenetic, epidemiological, and clinical features. At the molecular level, these three viruses were genus β-Coronavirus with nucleic acid similarity of 79.5% (SARS) and 54.9% (MERS), but the gene length was different (COVID-19, 29.9 kb; SARS, 29.7 kb, MERS, 30.1 kb). Moreover, the functional receptors for COVID-19 and SARS were angiotensin-converting enzyme 2 (ACE2), while it was (dipeptidyl peptidase 4) DPP4 for MERS [2]. Although we have the experience to deal with SARS and MERS, there are still many unknowns about COVID-19. This also resulted in the extremely serious epidemic situation, and the prevention and control pressure are still huge. Specific drugs and antibodies for the prevention and control of COVID-19 are still being studied, and there is still uncertainty about the same type of drugs that have been reported to have certain effects [3,4]. Remdesivir, which was developed to target SARS and MERS, has been reported with effects in inhibiting COVID-19 virus, but the effectiveness and safety are still being questioned [5]. Similarly, the antimalarial drugs, chloroquine, and hydroxychloroquine, have also been applied to fight the COVID-19 virus, but there are still concerns about their strong side effects [6]. Safer and more effective drugs to control COVID-19 are urgently needed.

Polypeptide drugs are widely used in the treatment of various human diseases such as pain, arthritis, cancer, hepatitis, and acquired immune deficiency syndrome (AIDS) due to their wide indications, high safety, and significant efficacy [7,8,9,10,11]. As most disease targets are proteins, polypeptide drugs have incomparable advantages and potentials in the binding ability and structural diversity comparing with small molecule drugs [12]. Moreover, their non-covalent binding to the targets has also been shown with the priority to the covalently bound drug in terms of controlling side effects. Nowadays, few covalent drugs are currently designed to treat anything other than cancer [13,14], and this may also explain the side effects of drugs such as Remdesivir and other covalently bound drugs used to treat COVID-19. Therefore, the screening of peptide drugs represents an important direction in the search for specific drugs to inhibit COVID-19.

In nature, peptides are mostly generated from the proteolysis, and food-derived peptides with 2 to 10 amino acids have been proven to have higher absorption efficiency and functional effects, including an antiviral effect [10,11]. In the face of the abundant varieties of peptide sources and structures, computer-aided screening is the first step in drug screening, one of which is the molecular modeling [15,16]. Although the molecular modeling cannot provide a definitive conclusion of the activity, the results may provide important information on initiating drug developments [17].

Based on the in-depth study on the infection mechanism of COVID-19 virus, three major action sites may be key targets for treatment, including COVID-19 virus spike protein receptor binding domain angiotensin-converting enzyme 2 (ACE2) [18,19], COVID-19 virus main protease (COVID-19 virus M^pro^) [20], and COVID-19 virus RNA-dependent RNA polymerase (COVID-19 virus RdRp) [21]. The research findings from our group and other investigators indicate that non-specific binding peptides may have similar inhibitory activity to ACE and ACE2 as they show highly homology [22]. Meanwhile, ACE is a type of metal ion-dependent enzyme with “active pocket” structure [23], and its inhibiting mechanism is quite similar to that of COVID-19 M^pro^ and RdRp; all contain an “active pocket or other molecule like metal ion, nucleotide” [20,21]. 

In this study, we first widely collected the food-derived peptides and then systematically studied the interaction between peptides and three key targets, ACE2, COVID-19 M^pro^, and RdRp. Through calculation and comparison, we evaluated the binding affinity of collected food-derived peptides with those key targets of COVID-19 virus and identify the most potential peptides that may inhibit those three targets and their interaction models, aiming to provide necessary information for the development of more effective drugs against COVID-19.

## 2. Material and Methods

### 2.1. Receptor and Ligands Preparation

The structure of human angiotensin-converting enzyme 2 (ACE2, the COVID-19 coronavirus spike protein receptor binding domain), COVID-19 coronavirus main protease (COVID-19 virus M^pro^), and RNA-dependent RNA polymerase (COVID-19 virus RdRp) were downloaded from the Protein Data Bank (PDB) website with necessary treatment. The structure of ACE2 was derived from “Crystal structure of SARS-CoV-2 spike receptor-binding domain bound with ACE2, PDB No. 6M0J” with deleting the spike protein [19]. The structure of COVID-19 virus M^pro^ was derived from “The crystal structure of COVID-19 main protease in complex with an inhibitor N3, PDB No. 6LU7” with deleting the inhibitor N3 (cysteine residues were replenished) [20]. The docking site for ACE2 was defined from the PDB Site Records and the interaction between ACE2 and COVID-19 virus spike protein. The structure of COVID-19 virus RdRp was derived from “Template RNA and Remdesivir bound RdRp complex, PDB No. 7BV2” with deleting the inhibitor Remdesivir (Uracil residues were replenished) [21]. 

The peptides were collected from the latest reviews on ACE-inhibitory peptides derived from the natural food resources including plant, animal, marine, and mushroom [23,24,25,26,27,28,29]. All the peptides were drawn using Chem3D software (Version 15.0, Cambridge Software Inc., Cambridge, MA, USA, 2018), and then, one step of optimization was employed to get the effective structure.

### 2.2. Molecular Docking Simulation

The inhibition mechanism of peptides against human angiotensin-converting enzyme 2 (ACE2, the COVID-19 coronavirus spike protein binding receptor), COVID-19 coronavirus main protease (COVID-19 virus M^pro^), and RNA-dependent RNA polymerase (RdRp) were conducted through the molecular docking and dynamic test using Discovery studio client software (v16.1.0.15350) (BIOVIA, San Diego, CA, USA) based on our previous methods [15,23,24]. MLN-4760, inhibitor N3, and Remdesivir were set as positive controls [18,20,21]. The main procedures were as follows. 

(a) Defining the active site: After loading the structure of receptor into Discovery studio client software (DS 4.0), all water molecules and ligands were removed, and the *conforming, hierarchical, adaptive refinement methods* (CHARMs) force field was applied using the PREPARE PROTEIN tool in DS 4.0. Then, we chose to define the active sites based on the active cavities of the receptor and PDB site records.

(b) Molecular docking: CDOCKER tools in DS 4.0 were used for the molecular docking. We first loaded the structure of peptides and three targets into DS 4.0 and then chose the active site described in Section 2.1. The CHARMs force field was a default during the whole docking process. 

(c) Conformation Scoring. After the docking process, the top 10 conformation poses were generated for each ligand based on docking score. The conformation with the highest CDocker energy (Score, Total Interaction Energy) was obtained (“-” represents inter-attraction). Then, the interaction energy of the ligand–receptor interaction was calculated, and interaction sites were illustrated in 2D and 3D images. Then, the main interaction forces and key amino acids could be identified.

(d) Analysis of peptides overlapping: Based on the calculation of interaction energy (absolute value), we could obtain a series of peptides with high potential inhibitory activities against the receptor. Through the overlapping of these peptides and analysis of surface properties, we could conclude the basic principle for drug searching.

### 2.3. Molecular Dynamic Simulation

To analyze the structure changes of the receptor including ACE2, COVID-19 virus M^pro^, and COVID-19 RdRp caused by molecule docking, the molecular dynamics simulation of the ligand–receptor complex was performed using the SIMULATION tool in the DS 4.0 with CHARMs force field. Specifically, the ligand–receptor complex was first pretreated in the absence of a water molecule, and later, a 7 Å solvation shell was added. At same time, 0.145 M NaCl was used to simulate the human environment. Two minimization cycles (steepest descent and conjugate gradient) were performed until the root mean square (RMS) of energy gradient was <0.1 kcal/mol·Å [15,30]. The steepest descent cycle was performed with 2000 steps (time step: 0.001 ps), while a conjugate gradient was performed with 1000 steps (time step: 0.001 ps). The SHAKE algorithm was applied throughout the molecular dynamics (MD) simulation to hold all the bonds involving hydrogen atoms. The long-range electrostatic forces were treated with the PME method. After minimization, the sample was gradually heated to a target temperature from 50 to 300 K over an interval of 5 ps. After this heating process, a 5000 steps-long (time step: 0.002 ps) equilibration phase was applied. The production stage was performed in 50,000 steps using a time step of 0.002 ps using NPT canonical assembly. The decay time for the temperature coupling was 5.0 ps. The whole complex structure changes were illustrated by highlighting the ligand and docking pocket comparing with the complex structure before molecular dynamic simulation. After the molecular dynamic simulation, 50 conformation poses were produced, and we could obtain the pose with the lowest total energy. The structure changes before MD and after MD could be analyzed, while the interaction between the ligand and receptor could also be illustrated in 2D and 3D images. 

## 3. Results

### 3.1. Candidate Peptides That Target ACE2

From the latest research studies, the COVID-19 virus could bind to ACE2 on the surface of host cells. By binding to ACE2, the virus starts the first step in cell invasion. Inhibition of virus binding to ACE2 may be an important strategy for preventing viral infections. Based on the crystalline structure of the COVID-19 virus spike protein receptor binding domain ACE2 complex, the main binding sites could be illustrated (Supplementary Materials, Figure S1A–C). By docking the peptides to the COVID-19 virus spike protein RBD of ACE2, we found 187 peptides that could bind to ACE2 with the -CDocker energy (Score, Total Interaction Energy) varying from 14.592 to 225.598 kcal/mol (Supplementary Materials, Table S1). For the reported inhibitor of ACE2, including MLN-4760 and DX600 (Supplementary Materials, Figure S1D,E), we also found that the MLN-4760 could bind to ACE2 at the COVID-19 virus spike receptor binding domain with the -CDocker energy (Score) of 21.028 kcal/mol (Supplementary Materials, Table S2; Figure S2A–E). The DX600 could not bind to ACE2 at this specific domain.

**Figure 1:**
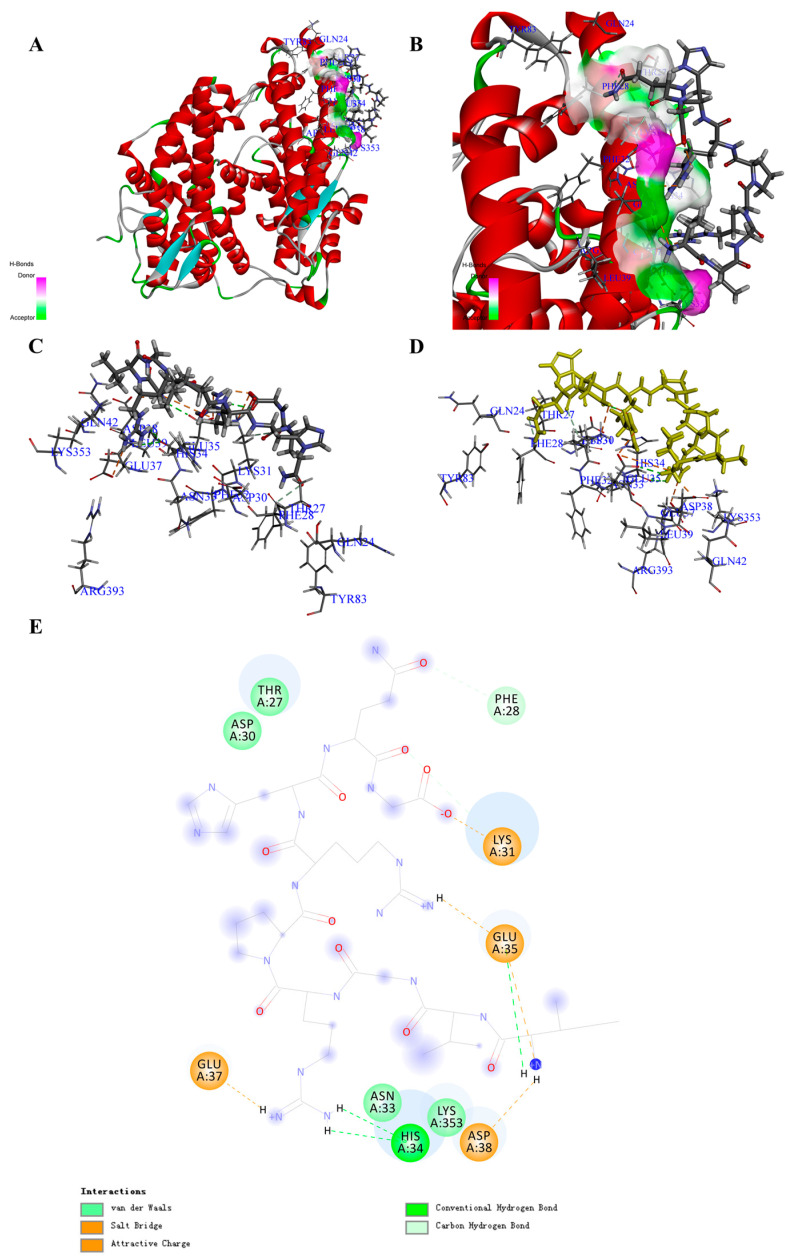
Structure of the COVID-19 virus spike protein receptor binding domain angiotensin-converting enzyme 2 (ACE2) complex containing potential inhibitory peptide (PA1) and its binding modes. (**A**) Three-dimensional (3D) structure of the ACE2 complex containing PA1. For clarity, the interface receptor amino acids residues are shown in thin stick style, and PA1 is shown in thick stick style. ACE2 is downloaded from PDB (No. 6M0J) and generated in DS software with deletion of the ligands. The different colors represent the different secondary structure: red is α-helix, blue is β-fold, green is β-turn, white is random coil. PA1 is the peptide, Ile-Val-Gly-Arg-Pro-Arg-His-Gln-Gly, with the highest total interaction energy to ACE2. (**B**) Enlarged view of the interface in the ACE2 complex containing PA1. (**C**,**D**) Binding site between PA1 and ACE2 and the main interaction residues. Dashed line with a different color represents the non-covalent interaction between PA1 and ACE2. (**E**) 2D representation of interactions between the PA1 and amino acid residues of ACE2.

Based on the screening by -CDocker energy (Score), we chose 30 peptides with the highest scores for the interaction analysis (Supplementary Materials, Table S2). Finally, we obtained the interaction counts, residues, and energy between peptides and ACE2, and the peptide having the highest total interaction energy was PA1 with an amino acids sequence of Ile-Val-Gly-Arg-Pro-Arg-His-Gln-Gly. The total interaction energy (absolute value) between PA1 and ACE2 was 330.556 kcal/mol, which was made up of 10 non-covalent bonds (Table 1; Supplementary Materials, Table S8). Specifically, five electrostatic (including five attractive charges and three salt bridges) and five H-bonds were formed between peptide and amino acid residues including GLU35, ASP38, GLU37, ASP30, HIS34, THR27, PHE32, GLN24, and ASN33 (Figure 1A–E; Supplementary Materials, Table S8). Comparing with the reported drugs, the total interaction energy (absolute value) for MLN-4760 was 86.407 kcal/mol, and the main interaction amino acid residues were GLU37, LYS31, ASP38, LYS353, THR27, GLU35, ASP30, and ASN33 (Table 2). The main forces for MLN-4760 were H-bond and hydrophobic (Supplementary Materials, Table S5). Through the calculation of interaction between the COVID-19 spike protein and ACE2, the total interaction energy (absolute value) was 158.342 kcal/mol, which was contributed by GLN24, ASP30, LYS31, HIS34, GLU35, ASP38, TYR41, GLN42, MET82, TYR83, LYS353, and GLY354 (Table 2 and Supplementary Materials, Figure S5). The main interaction forces were H-bond and hydrophobic interaction (Supplementary Materials, Table S5) [19]. The identified peptides that could bind to the receptor-binding domain of ACE2 with higher interaction energy are listed in Table 1 and Table 2.

The docking poses in Figure S5 clearly show how the MLN-4760 and virus spike protein bind to ACE2. The bonding region was the surface of two helical chains of ACE2, the middle region was highly hydrophilic, and both sides were highly hydrophobic. For the COVID-19 virus spike protein, it was like “handle” bound to ACE2, where lots of H-bond appeared in the middle region and hydrophobic interaction appeared on both sides.

Through overlapping the peptides of higher interaction energy bound to ACE2, we obtained the “portrayal” of the peptides that might be potential for the development of the natural inhibitory drug (Figure S3A–D). The peptide commonly had 9–10 amino acids (Ave. MW ≈ 1.14 KDa) (Table 3), with hydrophilic amino acid and acidic amino acid in the middle and aromatic amino acids on the side (Supplementary Materials, Figure S3E,F). The overlapping results could also show that the α-helix at SER19 to TYR50 was the main binding site, which was half covered by the peptides such as “tweezer” (Supplementary Materials, Figure S3G,H). This kind of site occupation and spatial obstruction might give the possibility for the peptides inhibiting the interaction of COVID-19 spike protein and ACE2 [31].

Moreover, after molecular dynamic simulation, we could obtain the most stable conformation with the lowest total energy. For instance, the PA1 could also show a much stronger interaction to ACE2. Comparing with the complex before molecular dynamic simulation, the total interaction energy (absolute value) between PA1 and amino acid residues increased from 330.556 to 440.463 kcal/mol (Supplementary Materials, Figure S4A–D), and the interaction bond counts increased from 10 to 15 with more H-bonds formed (Supplementary Materials, Tables S5 and S11; Figure S4E). This result suggests that PA1 may be a potential candidate bound to ACE2 for inhibiting the interaction of the COVID-19 virus, although further experimental verification is required. 

Docking results between 187 peptides and ACE2 could be found in Supplementary Materials, Table S1. The interaction analysis results of the 30 peptides with the highest -CDocker energy (Score) could be found in Supplementary Materials, Table S2.

### 3.2. Candidate Peptides That Target COVID-19 Virus M^pro^

The COVID-19 virus M^pro^ is another target that can directly stop the replication and construction of the enzyme and protein for the new virus. By docking the peptides to COVID-19 virus M^pro^ (Supplementary Materials, Figure S6A–D), a total of 167 peptides could successfully bind to the COVID-19 virus M^pro^, and the -CDocker energy (Score) varied from 21.009 kcal/mol to 131.774 kcal/mol (Supplementary Materials, Table S1).

**Figure 2:**
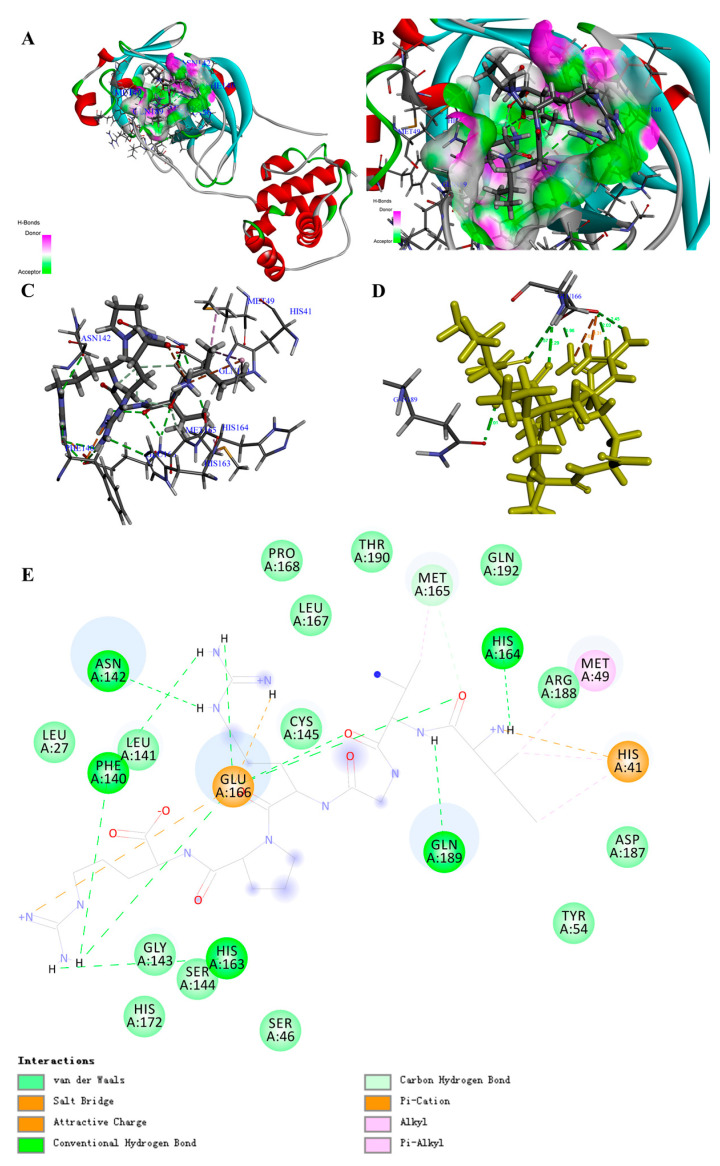
Structure of the COVID-19 virus main protease (COVID-19 virus Mpro) complex containing peptide (PM1) and its binding modes. (**A**) Three-dimensional (3D) structure of the COVID-19 virus M^pro^ complex containing PM1. For clarity, the interface receptor amino acids residues are shown in thin stick style and PM1 is shown in thick stick style. COVID-19 virus M^pro^ is downloaded from PDB (No. 6LU7) and generated in DS software with deleting the ligands. Different color represents different secondary structure, red is α-helix, blue is β-fold, green is β-turn, white is random coil. PM1 is the peptide, Ile-Val-Gly-Arg-Pro-Arg, with the highest total interaction energy to COVID-19 virus M^pro^. (**B**) Enlarged view of the interface in COVID-19 virus M^pro^ complex containing PM1. (**C**,**D**) Binding site between PM1 and COVID-19 virus M^pro^ and the main interaction residues. The dashed line with the different color represents the non-covalent interaction between PM1 and COVID-19 virus M^pro^. (**E**) 2D representation of interactions between PM1 and amino acid residues of COVID-19 virus M^pro^.

Based on the screening by -CDocker energy (Score), we chose 30 peptides with the highest score for the interaction analysis and obtained the interaction counts, residues, and energy between peptides and COVID-19 virus M^pro^ (Supplementary Materials, Table S3). For instance, the highest total interaction energy (absolute value) between peptide (Ile-Val-Gly-Arg-Pro-Arg) and COVID-19 virus M^pro^ was -366.214 kcal/mol, which was made up of 24 bonds (Table 1; Supplementary Materials, Table S9). Specifically, 2 attractive charges, 12 H-bonds, 1 Pi-Cation, 2 Alkyl, and 2 Pi-Alkyls were formed between the peptide and amino acid residues including HIS41, MET49, ASN142, GLY143, CYS145(SG), HIS164, MET165, GLU166, GLN189, and THR190 (Figure 2A–E; Supplementary Materials, Table S9). Comparing with the reported drugs, the total interaction energy (absolute value) for Ritonavir was 87.46983 kcal/mol, while it was -102 kcal/mol for Lopinavir (Table 2; Figure S6E,F). This result is in accordance with the clinical report of Lopinavir [32]. With regard to the interaction residues, GLY143, CYS145, MET165, GLU166, and GLN189 were the common targets to inhibit COVID-19 virus M^pro^, and the main interaction forces for Ritonavir and Lopinavir were H-bond and Pi bond (Supplementary Materials, Table S6). Through the in-depth review of the reported inhibitor N3 (Supplementary Materials, Figure S6D), we could also find the similar interaction sites. The main difference was that the interaction between inhibitor N3 and COVID-19 virus M^pro^ contained a covalent bond (C-S), which was the main interaction force (Supplementary Materials, Figure S7A–E; Table S6) [20], since the interaction energy (absolute value) of no-covalent bonds for inhibitor N3 was just 37.435 kcal/mol (Table 2). 

The docking poses in Figure S10 clearly illustrated that the active site of COVID-19 virus M^pro^ was similar to a deep “pocket”, and two hydrophobicity residues were guarded similar to a “door switch” (Supplementary Materials, Figure S8A–D). All the drugs and peptides (top 3 of total interaction energy (absolute value)) were inserted into the pocket site of the COVID-19 virus M^pro^.

Through overlapping the peptides of higher interaction energy, we obtained the “portrayal” of the peptides that might have potential for the development of natural inhibitory drugs to target COVID-19 virus M^pro^. The peptide commonly had five to six amino acids (Ave. MW ≈ 0.67 KDa) (Table 3), with a basic amino acid of the “head” section, an acidic amino acid of the “neck” section, a hydrophobicity group of the mid-section, and basic amino acids of the “tail” section (Supplementary Materials, Figure S8E–H).

Moreover, after molecular dynamic simulation, we could obtain the most stable conformation with the lowest total energy. For instance, the PM1 could also show stronger interaction to COVID-19 virus M^pro^ (Supplementary Materials, Figure S9A–D). Comparing with the complex before molecular dynamic simulation, the interaction bond counts between PM1 and amino acid residues increased from 19 to 29, although the total interaction energy (absolute value) varied from −366.21436 to −341.462362 kcal/mol (Supplementary Materials, Tables S6 and S11; Figure S9E). This result suggests that PM1 may be a potential candidate for inhibiting COVID-19 virus M^pro^. Certainly, further experimental verification and clinical trials are needed in the future.

Docking results between 187 peptides and COVID-19 virus M^pro^ are shown in Supplementary Materials, Table S1. The interaction analysis results of the 30 peptides with the highest -CDocker energy (Score) can be found in Supplementary Materials, Table S3.

### 3.3. Candidate Peptides That Target COVID-19 Virus RdRp

During the replication and transcription of viral genomes, the RNA-dependent RNA polymerase (RdRp) plays an important role that might be another target to prevent the transmission and infection of COVID-19 virus. By docking the peptides to COVID-19 virus RdRp (Supplementary Materials, Figure S11A–D), in total, we found 173 peptides that could bind to RdRp, and the -CDocker energy (Score) varied from −320.607 to 186.305 kcal/mol (Supplementary Materials, Table S1).

**Figure 3:**
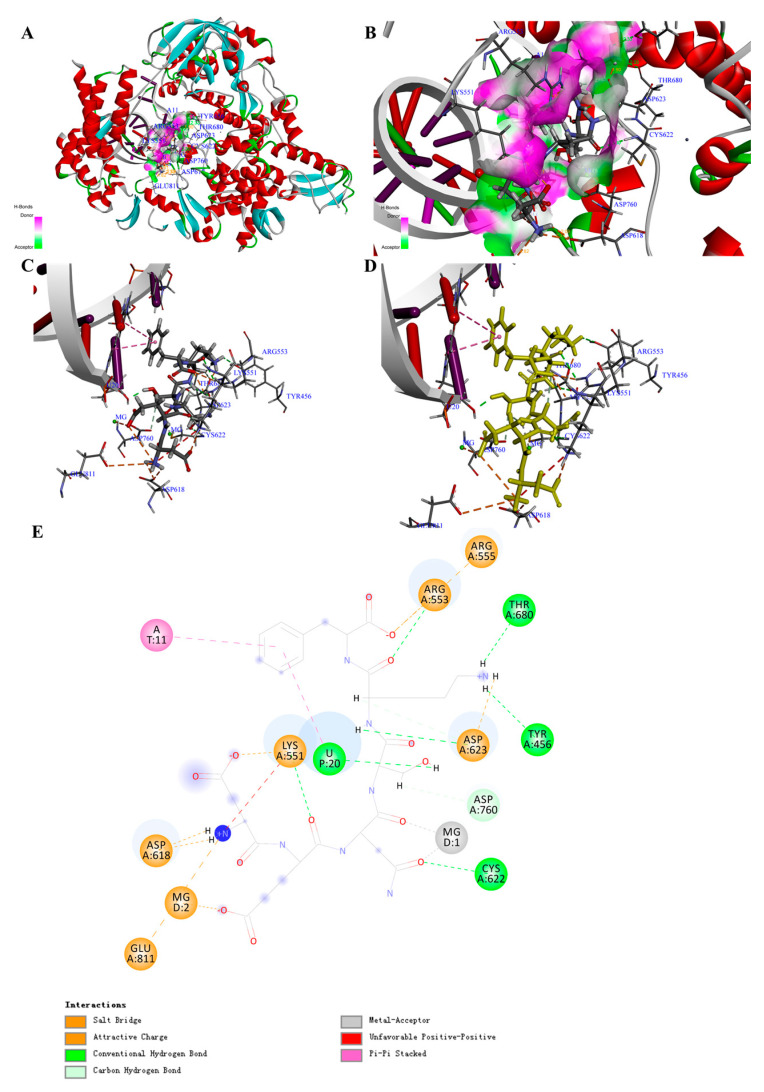
Structure of the COVID-19 virus RNA-dependent RNA polymerase (COVID-19 virus RdRp) complex containing peptide (PR1) and its binding modes. (**A**) Three-dimensional (3D) structure of the COVID-19 virus RdRp complex containing PR1. For clarity, the interface receptor amino acids residues are shown in thin stick style and PR1 is shown in thick stick style. COVID-19 virus RdRp is downloaded from PDB (No. 7BV2) and generated in DS software with deleting the RMP and POP ligands. Different colors represent different secondary structures: red is α-helix, blue is β-fold, green is β-turn, white is random coil. PR1 is the peptides, Asp-Glu-Asn-Ser-Lys-Phe, with the highest total interaction energy to COVID-19 virus RdRp. (**B**) Enlarged view of the interface in COVID-19 virus RdRp complex containing PR1. (**C**,**D**) Binding site between PR1 and COVID-19 virus RdRp and the main interaction residues. Dashed lines with different colors represent the non-covalent interaction between PR1 and COVID-19 virus RdRp. (**E**) Two-dimensional (2D) representation of interactions between PR1 and amino acid residues of COVID-19 virus RdRp.

By comparing the -CDocker energy (Score), we obtained 30 peptides with the highest scores, which might show higher binding energy and inhibitory activity. The interaction counts, residues, and energy between the peptides and COVID-19 virus RdRp were listed in Table S4. Peptide Asp-Glu-Asn-Ser-Lys-Phe (PR1) showed the highest total interaction energy (absolute value): 870.287 kcal/mol (Table 1), and the main interaction residues for COVID-19 virus RdRp were MG1, MG2, A11, U10, U20, ASP452, LYS545, LYS551, ARG553, ARG555, LYS621, ASP623, and LYS798 (Figure 3A–E). In total, 23 non-covalent bonds were formed between PR1 and COVID-19 virus RdRp. Specifically, eight electrostatic interactions, nine H-bonds, two metal acceptors, and four Pi-Pi stacked were the main interaction forces (Supplementary Materials, Table S10). The non-covalent interaction energy (absolute value) between the drug Remdesivir (Supplementary Materials, Figure S11D) and COVID-19 virus RdRp was 223.131 kcal/mol (Table 2; Supplementary Materials, Figure S11E,F), which was contributed by two electrostatic interactions, three H-bonds, and four hydrophobic interactions (Supplementary Materials, Figure S12A–E; Table S7). The O-P covalent bond between Remdesivir and RNA was thought to be a key mechanism for inhibiting RNA replication (Supplementary Materials, Figure S12D,E; Table S7) [21], and the main interaction amino acid residues for Remdesivir were MG1, MG2, U20, A11, LYS545, ARG555, CYS622, SER682, THR687, ASN691, and SER759, which were quite similar to PR1 (Table 2).

The results of docking poses in Figure S15 could clearly illustrate that the active site for COVID-19 virus RdRp was combined with RNA and the key domain was formed similar to a “pore canal” (Supplementary Materials, Figure S13A–D). All the drugs and peptides (top three of the total interaction energy (absolute value)) were inserted into the pocket site of the COVID-19 virus RdRp. Furthermore, the region away from RNA showed higher hydrophily, which was used to deliver the substrate for RNA polymerization.

Through overlapping the peptides of higher interaction energy, we obtained the “portrayal” of the peptides, which might indicate potential for the development of natural inhibitory drugs that target the COVID-19 virus RdRp. The peptide commonly had five to six amino acids (Ave. MW ≈ 0.67 KDa) (Table 3), with a basic amino acid and an acidic amino acid in the two sides and aromatic amino acids in the middle (Figure S13E–H).

Moreover, after molecular dynamic simulation, we could obtain the most stable conformation with the lowest total energy. For instance, the PR1 could show stronger interaction to COVID-19 virus RdRp (Supplementary Materials, Figure S14A–D). Comparing with the complex before molecular dynamic simulation, the interaction bond counts between PR1 and amino acid residues increased from 23 to 30, while the total interaction energy (absolute value) increased from 870.287 to 971.613 kcal/mol (Supplementary Materials, Tables S7 and S11; Figure S14E). This result suggests that PR1 may serve as a potential candidate for inhibiting COVID-19 virus RdRp. 

Docking results between 187 peptides and COVID-19 virus RdRp could be found in Supplementary Materials, Table S1. The interaction analysis results of the 30 peptides with highest -CDocker energy (Score) could be found in Supplementary Materials, Table S4.

## 4. Discussion

In this study, the results from molecular simulation demonstrate that certain naturally food-derived peptides may have inhibitory activities to the potential targets of COVID-19 virus with PA1 as a potential inhibitor for ACE2, PM1 as a potential inhibitor for M^pro^, and PR1 as a potential inhibitor for RdRp. All these peptides could be obtained from plants and animals such as soybean, peanut, corn, walnut, pork, chicken, salmon, sardine, etc. (Supplementary Materials, Table S1) 

In the processing of infection, COVID-19 virus directly binds to ACE2 with the help of the spike protein and then enters the cell through receptor-mediated endocytosis [18]. ACE2 has high homology with ACE, and in some reports, the ACE inhibitor has been shown to have the effect of slowing down the infection and damage of pneumonia risk [33]. Although it is unclear if the ACE inhibitor really works on COVID-19, the consensus is that inhibiting the interaction between ACE2 and the spike protein of COVID-19 could stop the infection. After docking 187 natural peptides to ACE2 in the area of the COVID-19 virus spike receptor binding domain, we found a series of peptides that could interact with ACE2. Specifically, for peptide PA1, the total interaction energy (absolute value) was more than twice that of the spike protein residues (158.34175 kcal/mol). For MLN-4760, we could indicate a certain interaction with ACE2 at the area of COVID-19 virus spike receptor binding domain, and for the ACE inhibitory peptides, results were similar. These results strongly supported our hypothesis that the ACE inhibitor could also bind to ACE2 and might show a potential effect to stop the interaction between ACE2 and the spike protein of COVID-19 virus. From our results, the α-helix at SER19 to TYR50 of ACE2 was the main active site interacting with the COVID-19 virus spike protein. Through the overlapping of the peptides with high total interaction energy, it suggested that the peptides with nine to 10 amino acids (Ave. MW ≈ 1.14KDa) might be more suitable for taking up the active sites. At the conformation level, peptides with hydrophilic amino acids, acidic amino acids in the middle, and aromatic amino acids on the two sides might cover the whole α-helix and reduce the interaction between hydrophilic groups of ACE2 and the spike protein. Moreover, after the molecular dynamic simulation, in the lowest energy conformation of the PA1–ACE2 complex, their interaction energy (absolute value) has increased from 330.556 to 440.463 kcal/mol and interaction counts have changed from 10 to 15 with more H-bonds formed. Meanwhile, the binding of peptides has also significantly changed the conformation of ACE2, especially in the area of the COVID-19 virus spike receptor binding domain. This also suggests that a peptide with certain sequences, structure, and conformation might be helpful for preventing the infection of COVID-19 virus at the initial stage through taking up the active sites and blocking their interaction. Moreover, comparing with the conventional ACE-inhibitory drugs, these peptides are of natural origin and do not produce toxic side effects when overused, which might help reduce the risk of traditional drugs when treating COVID-19 [34].

The proliferation of COVID-19 virus relies on a variety of non-structural proteins, including Main protease (M^pro^), Papain-like protease, Helicase (Nsp13), RNA-dependent RNA polymerase (RdRp), N-terminal exoribonuclease and C-terminal guanine-N7 methyl transferase (Nsp14), Uridylate-specific endoribonuclease (Nsp15), 2’-O-methyltransferase (Nsp16), and Nsp10 [21,35]. Among them, M^pro^ and RdRp are the main inhibitory targets reported. Nowadays, lots of drugs such as Remdesivir have been reported at the clinical level with a potential therapeutic effect against the COVID-19 virus. However, there are also many reports on their side effects. According to the latest research, the main interaction for Remdesivir was the covalent bond between Redesivir monophosphate (RMP) and uracil in RdRp [21,36]. Although the covalent bond may largely determine its inhibitory effect, the non-covalent interaction between the inhibitor and receptor also plays an important role. Actually, it is worth noting that the drug and target are permanently bound by a covalent bond, and this irreversibility will bring about a series of side effects [13]. For this reason, more and more researchers have confirmed that the non-covalent bond drug might be more useful. 

Here, our results showed that 163 peptides could bind to M^pro^, and 30 of them with the highest -CDocker energy (Score) had a total interaction energy (absolute value) over 64.279 kcal/mol. Peptides PM1 could bind to M^pro^ with the total interaction energy of 366.214 kcal/mol, while Ritonavir and Lopinavir showed the total interaction energy (absolute value) of 87.470 kcal/mol and 102.500 kcal/mol, respectively. This is consistent with the existing reports that Ritonavir and Lopinavir can be used to treat COVID-19. Meanwhile, from the latest research, inhibitor N3 also showed high interaction with M^pro^, which was mainly contributed by the C-S covalent bond between CYS145 and N3 and 23 non-covalent bonds [20]. From our results, peptide PM1 formed 19 non-covalent bonds, which is higher than Ritonavir and Lopinavir. Although there is no covalent bond formed between PM1 and M^pro^, the total non-covalent interaction energy is ten times comparing with inhibitor N3, and their interaction residues are mainly concreted at GLU166, GLN189, MET165, CYS145, HIS41, ASN142, and HIS164. This suggests that PM1 and other food peptides could have the potential for inhibiting COVID-19 virus M^pro^. In addition, it has been reported that the key inhibitory sites were located at the random coil that ranged from THR25 to ALA191 [20]. Our results showed all the peptides and reported drugs were well inserted into the “pocket” and the amino acid residues at GLN189 and ASN142 formed two conventional hydrogen bonds with peptides that function as a “door switch”, which results in the full occupancy of the key active sites. Moreover, from the overlapping of peptides, we can also find out that the peptides with five to six amino acids (Ave. MW ≈ 0.67 KDa) could bind to M^pro^, and at the conformation level, peptides with basic amino acids, aliphatic amino acids, and aromatic amino acids might well interact with the residues inside the pocket and also bind tightly to the “door switch”. In addition, after molecular dynamic simulation, the peptide PA1 was still inside the pocket, and more interaction bonds were formed, even though the total interaction energy (absolute value) was slightly reduced. However, the absolute value of the interaction energy was still higher than that of Ritonavir and Lopinavir and also higher than the non-covalent interaction energy of inhibitor N3. Therefore, our results support that certain peptides, especially PM1, might be good candidates for the drug development targeting the COVID-19 virus M^pro^.

Our screening found that 172 peptides could bind to RdRp, and 30 of them with the highest scores had the total interaction energy (absolute value) ranging from 213.13233 to 870.28747 kcal/mol. Peptide PR1 had the highest interaction energy with 23 formed bonds and the metal ion (MG) and the nucleotide as the main interaction residues. It has been reported that Remdesivir was metabolized into phosphates (RMP) and then interacted with the uracil (U20). The O-P covalent bond formed and the heterocyclic nitrogen of RMP was bound to the uracil (U10) through two H-bonds [21,36]. Through the calculation, its total non-covalent interaction energy (absolute value) is 233.131 kcal/mol, which is lower than PR1 (870.287 kcal/mol). Although there is no covalent bond formed between PR1 and RdRp, the total non-covalent interaction energy is four times that of Remdesivir. The main interaction residues are similar, including MG1, MG2, U20, A11, LYS545, and ARG555. This suggests that PR1 also could have potential for inhibiting COVID-19 virus RdRp. Our results also showed that the active sites have formed similar to a “pore canal” with higher hydrophilicity, which is used for the transporting of nucleotide substrates. At the end, the RNA chain binds to RdRp. It has been reported that the RdRp-inhibitory mechanism for Remdesivir was via the formation of covalent bonds to prevent the polymerization of RNA [21]. Our results also suggest that the non-covalent interaction between RMP and RdRp amino acid residues might block the “pore canal” and reduce the binding probability of the substrate to the active site. The overlapping results of 30 peptides show that they mostly have five to six amino acids (Ave. MW ≈ 0.67 KDa), show their tight interaction with RNA and MG, and give the brief structure predication of peptides with high interaction energy. Specifically, peptides show more basic charges close to RNA, while there are more acidic groups near the magnesium ions. Peptides with a higher proportion of basic amino acid in the end (close to RNA) and acidic groups in the middle (close to MG) may show higher interaction energy with RdRp. Through the molecular dynamic simulation, the ligand–receptor complex with the lowest energy has been obtained, and the interaction energy between peptides and RdRp has been strengthened. For PR1, the interaction energy has increased to 971.613 kcal/mol with the interaction counts of 30. This can be explained by the introduction of the third magnesium ion. It has been reported that the RdRp strongly relies on the magnesium ion, and comparing with the reported drugs, our results suggest that except for the interaction with RNA, a strong interaction between peptides and MG could make it potential candidate for inhibiting COVID-19 virus RdRp. 

## 5. Conclusions

Since the outbreak of COVID-19, there is an urgent need for effective prevention and treatment. Although many potential inhibitors have been reported at the clinical and laboratory levels, the development of drugs with natural, less side effects remains one of the top priorities. In this paper, we have proved that natural food-derived peptides could bind to ACE2, COVID-19 virus M^pro^, and RdRp with higher non-covalent interaction energy than reported drugs or inhibitors. We also obtained six peptides (Val-Ser-Gly-Ala-Gly-Arg-Tyr, Val-Met-Asp-Lys-Pro-Gln-Gly, Val-Ile-Glu-Lys-Tyr-Pro, Lys-Asp-Tyr-Arg-Leu, Asp-Glu-Asn-Ser-Lys-Phe, Asn-Asn-Asn-Pro-Phe-Lys-Phe) showing the potential inhibitory effect to all these three targets. Through the molecular dynamic simulation, we have also confirmed that the interaction between peptides and COVID-19 could be further strengthened, while the receptor–ligand complex tends to be structurally stable. All these results suggest that natural food peptides might be potential candidates for drug development to prevent and control the infection of COVID-19 virus. Our study results provide critically important evidence to prioritize further experimental research for the development of therapeutic drugs against the COVID-19 virus. The sequence, structure, and conformation of peptides could also benefit from the further optimization of drugs.

## Figures and Tables

**Table 1:** Docking results and binding sites for the potential peptides inhibiting the possible control targets of the COVID-19 virus.

Target	Drug Information	Main Interaction Residues	Non-Covalent Interaction		
			Total Interaction Energy (kcal/mol)	Total VDW Interaction Energy (kcal/mol)	Total Electrostatic Interaction Energy (kcal/mol)
ACE2	Ile-Val-Gly-Arg-Pro-Arg-His-Gln-Gly (PA1)	GLU35, ASP38, GLU37, ASP30, HIS34, THR27, PHE32, GLN24, ASN33	−330.55571	−39.05066	−291.50505
	Phe-Gln-Lys-Pro-Lys-Arg (PA2)	ASP30, GLU35, GLU37, ASP38, HIS34, THR27, PHE28	−283.75101	−30.18061	−253.5704
	Gly-Asp-Leu-Gly-Lys-Thr-Thr-Thr-Val-Ser-Asn-Trp-Ser-Pro-Pro-Lys-Tyr-Lys-Asp-Thr-Pro (PA3)	ASP30, GLU37, GLU35, LEU29, ASP38, THR27, LYS31, ALA386, ALA387,	−272.21947	−68.03403	−204.18544
M^pro^	Ile-Val-Gly-Arg-Pro-Arg (PM1)	HIS41, MET49, PHE140, ASN142, HIS163, HIS164, MET165, GLU166, GLN189	−366.21436	−52.81027	−313.40409
	Phe-Gln-Lys-Pro-Lys-Arg (PM2)	THR26, PHE140, GLY143, MET165, GLU166, ASP187, GLN189, THR190	−308.37838	−59.35911	−249.01927
	Tyr-Asn-Lys-Leu (PM3)	HIS41, PHE140, LEU141, ASN142, MET165, GLU166, GLN189	−306.44818	−54.43838	−252.00979
Rdrp	Asp-Glu-Asn-Ser-Lys-Phe (PR1)	MG1, MG2, A11, U10, U20, ASP452, LYS545, LYS551, ARG553, ARG555, LYS621, ASP623, LYS798	−870.28747	−44.73179	−825.55568
	Ile-Ala-Glu (PR2)	MG1, MG2, U20, LYS551, ARG553, ARG555, LYS621, CYS622	−737.55523	−14.8947	−722.66053
	Ile-Val-Val-Glu (PR3)	MG1, MG2, A19, U20, LYS551, ARG624, ARG553, LYS621, CYS622, ARG624, SER682	−613.53135	−25.86384	−587.66751

**Table 2:** Docking results and binding sites for the reported drugs and ligand residues inhibiting the possible targets of COVID-19 virus.

Target	Drug Information	Main Interaction Residues	Covalent Bond	Non-Covalent Interaction		
				Total Interaction Energy (kcal/mol)	Total VDW Interaction Energy (kcal/mol)	Total Electrostatic Interaction Energy (kcal/mol)
ACE2	MLN-4760 C_19_H_23_Cl_2_N_3_O_4_ CAS No.: 305335-31-3	GLU37, LYS31, ASP38, LYS353, THR27, GLU35, ASP30, ASN33	/	−86.40653	−26.30758	−60.09895
	COVID-19 virus Spike receptor binding domain	GLN24, ASP30, LYS31, ASP38, TYR41, GLN42, MET82, TYR83, LYS353, GLY354	/	−158.34175	3.79202	−162.13377
M^pro^	Inhibition N3 C_35_H_48_N_6_O_8_ CAS:NA	MET49, HIS41, LEU141, GLY143, CYS145, HIS163, HIS164, MET165, GLU166, GLN189, THR190	C-S	−36.018383	−6.372228	−29.646155
Rdrp	Remdesivir C_27_H_35_N_6_O_8_P CAS No.: 1809249-37-3	MG1, MG2, U20, A11, LYS545, ARG555, CYS622, SER682, THR687, ASN691, SER759	P-O	−223.13061	−24.07386	−199.05675

**Table 3:** The summary for the peptides with potential high inhibitory activity comparing with reported drugs (molecular docking simulation level).

Target	Content	Peptides (Based on the Data of Peptides with TOP 30 Interaction Energy)	Reported Drugs
ACE2	Interaction Counts	14	13
	Main Interaction Residues	LYS353, LYS31, ASP38, HIS34, GLU35, PHE390, GLU37, ASP30	GLU37, LYS31, ASP38, LYS353, THR27, GLU35, ASP30, ASN33
	Main Interaction Types	Salt Bridge, Attractive Charge, H-bond, Electrostatic, Hydrophobic	H-bond, Alkyl, Pi-Alkyl
	Typical Grops	Hydrophilic amino acid, acidic amino acid, Aromatic amino acids	hydrophilic amino acid, Aromatic amino acids
M^pro^	Interaction Counts	16	14 (including 1 Covalent Bond)
	Main Interaction Residues	GLU166, GLN189, MET165, CYS145, HIS41, ASN142, HIS164	GLU166, GLY143, GLN189, MET165, CYS145
	Main Interaction Types	H-bond, Salt Bridge, Attractive Charge, Pi-Sigma, Pi-Sulfur, Pi-Alkyl, Pi-Cation, Alkyl	Covalent bond, H-bond Pi-Sigma, Pi-Sulfur, Pi-Pi T-shaped, Alkyl, Pi-Alkyl
	Typical Grops	Basic amino acid, Aliphatic amino acid, Aromatic amino acids	Benzene ring, Aromatic five-membered heterocyclic ring
RdRp	Interaction Counts	18	11 (including 1 Covalent Bond)
	Main Interaction Residues	MG1, MG2, U20, U10, U18, A11, LYS551, LYS621, ASP452, LYS621, ARG553	MG1, MG2, U20, A11, LYS545, ARG555, CYS622, SER682, THR687, ASN691, SER759
	Main Interaction Types	Salt Bridge; Attractive Charge, H-bond, Metal-Acceptor	Covalent bond, H-bond, Attractive Charge, Pi-Pi Stacked
	Typical Grops	basic amino acid and acidic amino acid, Aromatic amino acids	phosphate group, Six-membered azacyclic compounds

## Data Availability

The data presented in this study are available on request from the corresponding author.

## Supplementary Materials

The following are available online at https://www.mdpi.com/2218-273X/11/2/330/s1, Table S1. Docking results of the natural food peptides inhibiting the COVID-19 virus M^Pro^. Table S2 Interaction counts, residues and energy of the peptides inhibiting ACE2. Table S3 Interaction counts, residues and energy of the peptides inhibiting the COVID-19 virus M^pro^. Table S4 Interaction counts, residues and energy of the peptides inhibiting the COVID-19 virus RdRp. Table S5 Interaction between reported drugs, Covid-19 virus Spike protein residues and ACE2. Table S6 Interaction mechanism between reported drugs and COVID-19 virus M^pro^. Table S7 Interaction mechanism between reported drugs and COVID-19 virus RdRp. Table S8 Interaction mechanism between screening peptides and the ACE2. Table S9 Interaction mechanism between screening peptides and the COVID-19 virus M^pro^. Table S10 Interaction mechanism between screening peptides and the COVID-19 virus RdRp. Table S11 Interaction mechanism between PA1, PM1, PR1 and three targets after molecular dynamic simulation. Figure S1 Structure of the ACE2 and reported inhibitory drugs. Figure S2. Structure of ACE2 complex containing Inhibitor MLN-4760 and its binding modes. Figure S3. Structure feature of the docking pocket based on the overlap inhibitors. Figure S4 Structure of the ACE2 complex containing PA1 and its binding modes after molecule dynamic simulation. Figure S5 Docking Poses of the top three peptides inhibiting ACE2 comparing with MLN-4760. Figure S6 Structure of the COVID-19 virus M^pro^ and reported inhibitory drugs. Figure S7 Structure of the COVID-19 virus M^pro^ complex containing Inhibitor N3 and its binding modes. Figure S8. Structure feature of the docking pocket based on the overlap inhibitors. Figure S9 Structure of the COVID-19 virus M^pro^ complex containing PM1 and its binding modes after molecule dynamic simulation. Figure S10 Docking Poses of the top three peptides inhibiting COVID-19 virus Mpro comparing with Inhibitor N3. Figure S11 Structure of the COVID-19 virus RdRp and reported inhibitory drugs. Figure S12 Structure of the COVID-19 virus RdRp complex containing Remdesivir and its binding modes. Figure S13 Structure feature of the docking pocket based on the overlap inhibitors. Figure S14 Structure of the COVID-19 virus RdRp complex containing PR1 and its binding modes after molecular dynamic simulation. Figure S15 Docking Poses of the top three peptides inhibiting COVID-19 virus RdRp comparing with Remdesivir.

## Abbreviations

CHARMS	conforming, hierarchical, adaptive refinement methods
MD	molecular dynamic
MG	magnesium ion
NPT	constant-pressure, constant-temperature
PME	particle-mesh-ewald
POP	phosphonium ion
RBD	receptor binding domain
RMP	Redesivir monophosphate
RMS	root mean square
