# Supplementary material for: Screening and Molecular Modeling Evaluation of Food Peptides to Inhibit Key Targets of COVID-19 Virus
Source: Biomolecules. 2021 Feb 22;11(2):330. doi: 10.3390/biom11020330 (PMC7926797; doi:10.3390/biom11020330)
Supplement: Supplementary file 1 [file biomolecules-11-00330-s001.pdf]

Supplemental Files

Supplemental Tables

Table S1. Docking results of the natural food peptides inhibiting the COVID-19 virus M^{Pro}.

**Peptides (Amino Acid Sequences, N-C)**	**Resources**	**Molecular Weight (KDa)**	**IC50 for ACE inhibitory (μM)**	**CDocker Energy** (Target to ACE2)	**CDocker Energy** **(**Target to M^{pro}**)**	**CDocker Energy** **(**Target to RdRp**)**
Gly-Pro-Leu	Alaska pollack	0.29	2.65	39.1395	54.8402	71.7642
Gly-Pro-Met	Alaska pollack	0.3	17.13	36.0879	48.3654	73.0383
Val-Glu-Cys-Tyr-Gly-Pro-Asn-Arg-Pro-Gln-Phe	Algae protein waste (Chlorella vulgaris)	1.31	29.6	110.681	0 pose	0 pose
Lys-Leu-Lys-Phe-Val	Antartic krill	0.63	30	92.4957	113.828	121.897
Val-Arg	Atlantic salmon (Salmo salar L.) skin	0.27	1214	55.0572	60.9173	80.2457
Ala-Pro	Atlantic salmon (Salmo salar L.) skin	0.19	322	29.3173	34.9225	56.2805

Val-Ser-Gly-Ala-Gly-Arg-Tyr	Bitter melon seed	0.71	8.64	97.5157	96.0571	139.931
Glu-Val-Met-Ala-Gly-Asn-Leu-Tyr-Pro-Gly	Blue mussel (Mytilus edulis)	1.05	18.4	107.701	0 pose	0 pose
His-Glu-Arg-Asp-Pro-Thr-His-Ile-Lys-Trp-Gly-Asp	Bonito	1.49	8	152.459	0 pose	0 pose
Ile-Val-Gly-Arg-Pro-Arg-His-Gln-Gly	Bonito	1.02	2.4	91.5994	0 pose	-320.607
Ile-Lys-Pro-Leu-Asn-Tyr	Bonito	0.75	43	82.2876	95.329	129.328
Asp-Tyr-Gly-Leu-Tyr-Pro	Bonito	0.73	62	79.2338	95.8259	143.327
Ile-Val-Gly-Arg-Pro-Arg-His	Bonito	0.83	300	78.2676	0 pose	99.1444
Ile-Trp-His-His-Thr	Bonito	0.69	5.8	77.7969	97.2244	128.441
Ile-Val-Gly-Arg-Pro-Arg	Bonito	0.7	300	71.081	99.5343	101.812
Leu-Lys-Pro-Asn-Met	Bonito	0.6	2.4	67.1057	92.8846	115.571
Ala-Leu-Pro-His-Ala	Bonito	0.51	10	57.5337	84.785	111.241
Ile-Trp-His	Bonito	0.45	3.5	55.5474	65.1508	102.623
Leu-Lys-Pro	Bonito	0.36	0.32	51.2789	61.4139	76.3937
Ile-Lys-Pro	Bonito	0.36	6.9	50.9351	66.526	78.0459
Phe-Gln-Pro	Bonito	0.39	12	41.7034	47.4254	87.186
Tyr-Pro-Lys	Broccoli	0.41	10.5μg/mL	54.8054	73.2083	86.04
Tyr-Gln-Tyr	Buckwheat	0.47	4	70.0438	78.5407	106.41
Ile-Asn-Ser-Gln	Buckwheat	0.46	36	66.6592	82.2977	119.605
Val-Lys	Buckwheat	0.25	13	58.9498	69.2361	77.0963
Ile-Thr-Phe	Buckwheat	0.38	49	57.774	64.9095	100.366
Leu-Gly-Ile	Buckwheat	0.3	29	57.404	63.7958	88.9747
Leu-Phe	Buckwheat	0.28	126	44.8015	60.2472	78.8194

Pro-Ser-Tyr	Buckwheat	0.37	16	31.1784	48.3299	81.9871
Gly-Pro-Pro	Buckwheat	0.27	6.25μg/mL	24.4807	31.2158	53.7009
Phe-Gln-Lys-Pro-Lys-Arg	Chicken	0.8	14	88.6762	108.56	114.122
Phe-Lys-Gly-Arg-Tyr-Tyr-Pro	Chicken	0.93	0.55	87.6715	0 pose	129.016
Leu-Lys-Ala	Chicken	0.33	8.5	65.3697	78.9901	96.0038
Ile-Lys-Trp	Chicken	0.45	0.21	64.9252	76.5321	90.6945
Leu-Ala-Pro	Chicken	0.3	3.2	35.0494	51.1808	75.1243
Ile-Val-Val-Glu	Chlorella vulgaris	0.46	315.3	69.7253	84.3935	142.345
Ala-Glu-Leu	Chlorella vulgaris	0.33	57.1	61.573	67.722	119.378
Phe-Ala-Leu	Chlorella vulgaris	0.35	26.3	58.9602	61.567	100.895
Ala-Phe-Leu	Chlorella vulgaris	0.35	63.8	57.2925	70.5206	94.7731
Val-Val-Pro-Pro-Ala	Chlorella vulgaris	0.48	79.5	36.2395	50.163	85.2355
Ala-Tyr	Corn	0.25	14.2	48.7742	60.1951	81.0812
Lys-Pro	Fish sauce	0.24	-	35.256	49.2011	60.2831
Arg-Pro	Fish sauce	0.27	-	29.6649	44.054	55.497
Asn-Tyr	Garlic	0.3	32.6	51.9439	64.445	90.5215
Asn-Phe	Garlic	0.28	46.3	49.8824	57.4418	91.837
Ser-Tyr	Garlic	0.27	66.3	49.201	60.2226	77.8788
Ser-Phe	Garlic	0.25	130.2	45.3901	59.1071	80.9671

Tyr-Asn	Hard clam (Meretrix lusoria)	0.3	51	49.9959	55.5418	92.34
Lys-Asp-Tyr-Arg-Leu	Mung bean	0.69	26.5	104.907	109.957	151.06
Lys-Leu-Pro-Ala-Gly-Thr-Leu-Phe	Mung bean	0.85	13.4	78.2686	0 pose	143.249
Val-Thr-Pro-Ala-Leu-Arg	Mung bean	0.66	82.4	63.3964	85.6367	117.655
Gly-Val-His-His-Ala	Muscle of cuttlefish	0.52	71.8	75.369	98.9819	122.472
Ala-His-Ser-Tyr	Muscle of cuttlefish	0.48	11.6	73.9002	79.4709	118.176
Asp-Phe-Gly	Muscle of cuttlefish	0.34	44.7	62.8213	71.7534	128.37
Val-Ile-Ile-Phe	Muscle of cuttlefish	0.49	8.7	61.1591	76.6925	108.835
Met-Ala-Trp	Muscle of cuttlefish	0.41	16.32	56.3364	64.9407	96.9609
Ala-Val-Val	Muscle of cuttlefish	0.29	66.6	56.3083	69.5332	88.3258
Gly-Asp-Ala-Pro	Muscle of cuttlefish	0.36	22.5	56.3081	64.797	107.859
Ala-Gly-Ser-Ser	Muscle of cuttlefish	0.32	672.1	56.2894	75.9675	101.166
Ile-Ala-Val	Muscle of cuttlefish	0.3	153.4	54.549	58.1217	91.6562
Gly-His-Gly	Muscle of cuttlefish	0.27	122	53.9345	65.4526	91.1758

Ala-Gly-Ser-Pro	Muscle of cuttlefish	0.33	37.2	51.592	54.4728	80.6016
Val-Tyr-Ala-Pro	Muscle of cuttlefish	0.45	6.1	50.9287	64.1419	91.5151
Ala-Gly-Ser	Muscle of cuttlefish	0.23	527.9	50.6913	61.6507	88.9137
Phe-Gly-Gly	Muscle of cuttlefish	0.28	82.5	49.8777	59.9647	94.6106
Arg-Leu-Ser-Gly-Gln-Thr-Ile-Glu-Val-Thr-Ser-Glu-Tyr-Leu-Phe-Arg-His	Mushroom	2.04	1140	189.618	0 pose	0 pose
Arg-Leu-Pro-Ser-Glu-Phe-Asp-Leu-Ser-Ala-Phe-Leu-Arg-Ala	Mushroom	1.62	460	166.189	0 pose	0 pose
Val-Ile-Glu-Lys-Tyr-Pro	Mushroom	0.75	97μg	89.96	99.7399	147.539
Gly-Glu-Pro	Mushroom	0.3	40μg	44.6213	51.1087	98.0567
Phe-Tyr	oyster	0.33	-	47.9542	59.3323	87.2182
Ala-Trp	oyster	0.28	-	45.5299	58.3324	84.4831
Gly-Trp	oyster	0.26	-	44.4327	59.4702	82.5931
Val-Val-Tyr-Pro-Trp-Thr-Gln-Arg-Phe	Oyster (Crassostrea talienw hanensis Crosse)	1.2	66	99.7855	0 pose	0 pose
Lys-Glu-Asp-Asp-Glu-Glu-Glu-Glu-Gln-Glu-Glu-Glu	Pea	1.54	64.04	179.736	0 pose	0 pose
Asn-Asn-Asn-Pro-Phe-Lys-Phe	Peanut	0.88	74	95.5468	93.9746	130.481
Lys-Leu-Tyr-Met-Arg-Pro	Peanut	0.81	5.2	73.1097	91.7287	121.053

Arg-Met-Leu-Gly-Gln-Thr-Pro-Thr-Lys	pork	1.03	34	93.6283	0 pose	119.099
Arg-Met-Leu-Gly-Gln-Thr-Pro	pork	0.8	503	78.7074	82.658	18.099
Thr-Thr-Asn	pork	0.33	672.7	59.3562	68.8339	103.856
Met-Asn-Pro-Pro-Lys	pork	0.59	945.5	55.863	74.4531	99.7062
Ile-Thr-Thr-Asn-Pro	pork	0.54	549	54.6761	71.5716	108.947
Ile-Thr-Thr	pork	0.33	678.2	52.2229	61.4292	94.7824
Thr-Asn-Pro	pork	0.33	207.4	41.2421	53.778	81.1258
Met-Asn-Pro	pork	0.36	66.6	41.1995	54.8968	74.7071
Pro-Pro-Lys	pork	0.34	>1000	31.3164	45.9001	54.0093
Asn-Pro-Pro	pork	0.33	290.5	30.5809	40.2884	70.9078
Tyr-Ser-Lys	Rice	0.4	76	67.1826	82.1003	97.9799
Thr-Gln-Val-Tyr	Rice	0.51	18.2	49.4336	92.9703	128.223
Trp-Met	salmon	0.34	96.6	45.3623	57.2926	82.3442
Trp-Ala	salmon	0.28	277.3	45.2982	54.2807	87.7365
Leu-Trp	salmon	0.32	17.4	44.9223	60.2372	80.3438
Met-Trp	salmon	0.34	9.9	44.9105	58.6607	76.9788
Ile-Trp	salmon	0.32	4.7	43.9203	47.393	82.3783
Val-Trp	salmon	0.3	2.5	42.9988	52.0641	80.9498
Ala-Lys-Lys	sardine	0.35	3.13	72.9772	88.5264	93.086
Arg-Phe-His	sardine	0.46	330	58.3606	82.5693	90.0671
Lys-Tyr	sardine	0.31	1.63	56.9444	67.4777	92.5333
Arg-Val-Tyr	sardine	0.44	205.6	53.7134	73.2173	99.0151
Val-Tyr	sardine	0.28	10	47.7018	59.9214	79.1668
Arg-Tyr	sardine	0.34	51	46.4787	62.0804	79.2417
Gly-Trp-Ala-Pro	sardine	0.43	3.86	46.1926	54.7507	94.1058
Met-Tyr	sardine	0.31	193	46.017	60.7401	79.4418

Gly-Arg-Pro	sardine	0.33	20	45.1127	54.9556	71.9237
Met-Phe	sardine	0.3	44.7	45.0199	56.7105	86.9169
Val-Ile-Phe	Sea bream scales	0.38	7.5	51.1304	65.8012	97.2221
Gly-Tyr	Sea bream scales	0.24	265	44.3012	56.0515	78.6358
Gly-Phe	Sea bream scales	0.22	708	42.5349	58.6997	73.2081
Met-Glu-Gly-Ala-Gln-Glu-Ala-Gln-Gly-Asp	Sea cucumber (Acaudina molpadioidea)	1.04	15.9	128.97	0 pose	-94.3087
Glu	Sea cucumber gelatin	0.15	0.0142mg/mL	42.8858	43.4469	100.655
Asp	Sea cucumber gelatin	0.13	0.0142mg/mL	39.5067	40.9199	97.8258
Ala	Sea cucumber gelatin	0.09	0.0142mg/mL	32.1803	34.5972	55.5184
Gly	Sea cucumber gelatin	0.08	0.0142mg/mL	31.9556	33.3468	54.0535
Pro	Sea cucumber gelatin	0.12	0.0142mg/mL	14.5923	21.0092	36.6503
His-Trp-Thr-Thr-Gln-Arg	Seaweed pipefish (Syngnathus schlegeli)	0.83	1570	94.9058	119.623	145.482

Thr-Phe-Pro-His-Gly-Pro	Seaweed pipefish (Syngnathus schlegeli)	0.65	833	46.7665	69.5194	99.0912
Glu-Tyr	Shark meat	0.31	2.68	59.3942	64.9632	107.739
Phe-Glu	Shark meat	0.29	1.45	56.7751	59.4203	114.737
Cys-Phe	Shark meat	0.27	1.96	44.4003	52.8926	79.4626
Lys-Pro-Pro-Glu-Thr-Val	Shrimp (Acetes chinensis)	0.67	24.1	69.2369	91.1622	153.762
Phe-Cys-Val-Leu-Arg-Pro	Shrimp (Acetes chinensis)	0.73	12.3	69.1027	74.7753	105.837
Ile-Phe-Val-Pro-Ala-Phe	Shrimp (Acetes chinensis)	0.69	3.4	61.9887	77.7556	113.017
Asp-Phe	Shrimp (Acetes chinensis)	0.28	2.15	53.7645	59.932	110.527
Gly-Thr-Gly	Shrimp (Acetes chinensis)	0.23	5.54	49.1007	60.9424	85.9278
Ser-Thr	Shrimp (Acetes chinensis)	0.21	4.03	44.8186	55.5176	74.6754
Gln-Leu-Gly-Phe-Leu-Gly-Pro-Arg	Skate skin	0.89	148	94.9958	0 pose	-90.7701

Pro-Gly-Pro-Leu-Gly-Leu-Thr-Gly-Pro	Skate skin	0.81	95	51.2588	65.933	99.5353
Val-Met-Asp-Lys-Pro-Gln-Gly	Soybean	0.77	39	99.2486	113.728	173.231
Leu-Ile-Val-Thr-Gln	Soybean	0.57		83.0311	97.4666	125.822
Tyr-Val-Val-Phe-Lys	Soybean	0.65	44	82.6515	104.851	135.47
Leu-Ala-Ile-Pro-Val-Asn-Lys-Pro	Soybean	0.85	70	82.4624	78.3288	109.02
Tyr-Leu-Ala-Gly-Asn-Gln	Soybean	0.67	14	77.2252	108.513	152.18
Pro-Asn-Asn-Lys-Pro-Phe-Gln	Soybean	0.84	33	68.819	84.5337	115.44
Asn-Trp-Gly-Pro-Leu-Val	Soybean	0.68	21	68.2657	92.3658	127.268
His-His-Leu	Soybean	0.41	2.2μg/mL	63.973	74.5858	96.8476
Ile-Tyr-Leu-Leu	Soybean	0.52	42	61.2862	83.3561	118.551
Phe-Phe-Leu	Soybean	0.43	37	58.0166	71.2733	95.5043
Val-Leu-Ile-Val-Pro	Soybean	0.54	1.69	56.7937	69.0061	106.204
Asp-Gly	Soybean	0.19	12.3	56.7062	52.7829	97.7544
Asp-Leu-Pro	Soybean	0.34	4.8	52.5041	56.2467	107.716
Ile-Phe-Leu	Soybean	0.39	44.8	51.8395	65.8355	95.9672
Ile-Pro-Pro-Gly-Val-Pro-Tyr-Trp-Thr	Soybean	1.03	64	48.8103	0 pose	55.1015
Trp-Leu	Soybean	0.32	29.9	44.5778	57.657	
Ile-Ala	Soybean	0.2	153	43.1437	53.6416	82.4815
Ile-Ala-Tyr-Lys-Pro-Ala-Gly	spinach	0.72	4.2	87.7535	77.7556	144.336
Met-Arg-Trp-Arg-Asp	spinach	0.76	2.1	81.3465	106.999	158.88
Leu-Arg-Ile-Pro-Val-Ala	spinach	0.67	0.38	60.9283	93.3838	111.265
Met-Arg-Trp	spinach	0.49	0.6	60.4825	69.3185	94.8687
Ile-Ala-Glu	Spirulina platensis	0.33	34.7	61.4131	70.3608	124.363
Val-Ala-Phe	Spirulina platensis	0.34	35.8	52.0633	61.9796	86.5646

Ile-Ala-Pro-Gly	Spirulina platensis	0.36	11.4	43.1264	52.1073	86.2585
Gly-Pro-His-Tyr-Gly-His-Tyr-His-Tyr-Gly-Phe-Leu-Gly-Pro-His-Tyr-Gly-His-Tyr-Ser	Squid gelatin	2.35	256.82	181.374	0 pose	0 pose
Gly-Pro-Leu-Gly-Leu-Leu-Gly-Phe-Leu-Gly-Pro-Leu-Gly-Leu-Ser	Squid gelatin	1.41	90.03	116.84	0 pose	0 pose
Phe-Val-Asn-Pro-Gln-Ala-Gly-Ser	Sunflower	0.82	6.9	86.7603	90.8816	124.075
Asp-Glu-Asn-Ser-Lys-Phe	Terminalia chebula Tree	0.74	100uM	116.49	131.774	186.305
Gly-Asp-Leu-Gly-Lys-Thr-Thr-Thr-Val-Ser-Asn-Trp-Ser-Pro-Pro-Lys-Tyr-Lys-Asp-Thr-Pro	tuna	2.29	11.28	225.598	0 pose	0 pose
Trp-Pro-Glu-Ala-Ala-Glu-Leu-Met-Met-Glu-Val-Asp-Pro	tuna	1.52	21.6	124.607	0 pose	0 pose
Pro-Thr-His-Ile-Lys-Trp-Gly-Asp	tuna	0.95	-	102.88	0 pose	95.2256
Met-Ile-Phe-Pro-Gly-Ala-Gly-Gly-Pro-Glu-Leu	tuna	1.09	26.3	91.3204	0 pose	17.3319
Tyr-Asn-Lys-Leu	Wakame	0.54	21	83.009	108.759	118.127
Lys-Phe-Tyr-Gly	Wakame	0.51	90.5	81.6918	91.5439	114.986
Ala-Ile-Tyr-Lys	Wakame	0.49	213	79.4384	92.1615	116.461
Tyr-Lys-Tyr-Tyr	Wakame	0.64	64.2	76.3791	98.2529	132.235
Trp-Pro-Glu-Arg-Pro-Pro-Gln-Ile-Pro	Walnut	1.12	25.67μg/mL	65.6935	0 pose	0 pose
Asp-Tyr-Val-Gly-Asn	Wheat	0.57	0.72	82.3448	103.513	148.806
Asp-Ile-Gly-Tyr-Tyr	Wheat	0.63	3.4	81.919	99.3684	151.02
Thr-Tyr-Leu-Gly-Ser	Wheat	0.54	0.86	76.6415	95.5223	124.406

Ala-Pro-Gly-Ala-Gly-Val-Tyr	Wheat	0.63	1.7	71.6609	83.808	131.568
Gly-Gly-Val-Ile-Pro-Asn	Wheat	0.56	1.74	64.8567	86.2326	125.609
Thr-Val-Val-Pro-Gly	Wheat	0.47	2.2	57.4289	69.5636	104.557
Ile-Val-Tyr	Wheat	0.39	0.48	52.4483	65.7914	98.7118
Thr-Val-Pro-Tyr	Wheat	0.48	2	49.4336	69.2129	106.453
Ile-Tyr	Wheat	0.29	2.1	48.9221	55.8944	84.8079
Tyr-Leu	Wheat	0.29	16.4	47.3905	60.2082	91.7647
Leu-Tyr	Wheat	0.29	6.4	47.1263	62.6824	82.3029
Thr-Phe	Wheat	0.27	17.8	46.8764	60.8154	80.0819
Thr-Ala-Pro-Tyr	Wheat	0.45	13.6	46.6614	61.9975	0 pose
Val-Phe-Pro-Ser	Wheat	0.45	0.46	45.6551	58.6882	84.0747
Ala-Phe	Wheat	0.24	15.2	45.1201	58.9469	77.5333
Val-Phe	Wheat	0.26	9.2	43.9864	52.8629	86.6991
Ile-Ala-Pro	Wheat	0.3	2.7	41.3319	48.2411	74.0103
Tyr-Tyr-Ala-Pro-Phe-Asp-Gly-Ile-Leu	wine	1.06	83	91.9443	0 pose	-209.268
Trp-Val-Pro-Ser-Val-Tyr	wine	0.75	25.7	71.7613	85.2863	115.459
Ser-Trp-Ser-Phe	wine	0.53	76.3	64.2405	85.4116	113.219
Tyr-Tyr-Ala-Pro-Phe	wine	0.66	26.4	56.5007	79.0219	108.536
Ala-Trp-Pro-Phe	wine	0.52	18.3	47.5761	66.3371	103.157
Ile-Pro-Pro-Gly-Val-Pro-Tyr	wine	0.74	17.5	40.464	30.8374	92.0471
				80.1308	0 pose	85.8086

Table S2 Interaction counts, residues and energy of the peptides inhibiting ACE2.*

Peptides	MW	Interaction Residues	Total Interaction Energy (kcal/mol)	Total VDW Interaction Energy (kcal/mol)	Total Electrostatic Interaction Energy (kcal/mol)
Ile-Val-Gly-Arg-Pro-Arg-His-Gln-Gly	1.02	GLU35, ASP38, GLU37, ASP30, HIS34, THR27, PHE32, GLN24, ASN33	-330.55571	-39.05066	-291.50505
Phe-Gln-Lys-Pro-Lys-Arg	0.8	ASP30, GLU35, GLU37, ASP38, HIS34, THR27, PHE28	-283.75101	-30.18061	-253.5704
Gly-Asp-Leu-Gly-Lys-Thr-Thr-Thr-Val-Ser-Asn-Trp-Ser-Pro-Pro-Lys-Tyr-Lys-Asp-Thr-Pro	2.29	ASP30, GLU37, GLU35, LEU29, ASP38, THR27, LYS31, ALA386, ALA387,	-272.21947	-68.03403	-204.18544
Pro-Thr-His-Ile-Lys-Trp-Gly-Asp	0.95	ASP30, LYS68, LYS31, HIS34, GLN42, THR27, LEU39	-271.67106	-37.23784	-234.43322
Lys-Glu-Asp-Asp-Glu-Glu-Glu-Glu-Gln-Glu-Glu-Glu	1.54	LYS353, ARG393, LYS31, ARG559, ASN33, PHE390, PRO389	-264.82035	-47.29192	-217.52843
Val-Val-Tyr-Pro-Trp-Thr-Gln-Arg-Phe	1.2	ASP30, GLU35, GLU37, ASP38, LYS68, LYS31	-257.80548	-38.67454	-219.13094
His-Trp-Thr-Thr-Gln-Arg	0.83	GLU37, ASP30, LYS31, ASP350, ASP38, ALA386, THR27, ASN33, HIS34, PHE356	-253.97496	-45.45561	-208.51934
Lys-Asp-Tyr-Arg-Leu	0.69	ASP30, ASP38, GLU35, LYS31, GLU37, THR27, HIS34	-213.45445	-26.10099	-187.35346
Arg-Leu-Pro-Ser-Glu-Phe-Asp-Leu-Ser-Ala-Phe-Leu-Arg-Ala	1.62	GLU37, ASP30, LYS31, ASP38, GLU35, HIS34, GLN388, GLN76, THR27, PHE28	-209.91021	-60.94457	-148.96564

Phe-Val-Asn-Pro-Gln-Ala-Gly-Ser	0.82	ASP30, LYS31, LYS353, HIS34, ASP38	-208.71607	-30.06295	-178.65312
Arg-Met-Leu-Gly-Gln-Thr-Pro-Thr-Lys	1.03	ASP30, LYS353, GLU35, ASP355, TYR41, PHE28, GLY354	-207.89889	-40.75087	-167.14802
Lys-Leu-Lys-Phe-Val	0.63	GLU35, ASP30, ASP38, LYS353, HIS34	-201.1654	-22.51514	-178.65026
Gly-Pro-His-Tyr-Gly-His-Tyr-His-Tyr-Gly-Phe-Leu-Gly-Pro-His-Tyr-Gly-His-Tyr-Ser	2.35	ASP30, LYS31, ASP38, ALA387, HIS34, GLU37, LEU39, LYS68, ASN90, THR92	-196.42645	-61.79483	-134.63163
Gln-Leu-Gly-Phe-Leu-Gly-Pro-Arg	0.89	GLU35, ASP30, GLU37, ASP38, HIS34, LYS353, GLN42	-182.86518	-36.162	-146.70318
Val-Ser-Gly-Ala-Gly-Arg-Tyr	0.71	ASP30, GLU35, LYS353, ALA36, THR27, ASP38, LEU39	-182.41406	-30.94987	-151.46419
Phe-Lys-Gly-Arg-Tyr-Tyr-Pro	0.93	GLU37, GLU35, ASP30, ASP38, HIS34, THR27	-182.14174	-38.69778	-143.44396
Ile-Ala-Tyr-Lys-Pro-Ala-Gly	0.72	ASP30, LYS353, GLU35, GLU37, GLN42, HR27, HIS34, LEU39	-176.27213	-30.06695	-146.20518
Asn-Asn-Asn-Pro-Phe-Lys-Phe	0.88	GLU37, ASP30, LYS31, HIS34, ASP38, GLU35, THR27	-176.26119	-36.84469	-139.4165
Trp-Pro-Glu-Ala-Ala-Glu-Leu-Met-Met-Glu-Val-Asp-Pro	1.52	LYS353, LYS31, ARG393, GLU75, LYS68, GLN42, GLY354, LEU39, THR27, PHE72	-175.65145	-52.19757	-123.45388
Glu-Val-Met-Ala-Gly-Asn-Leu-Tyr-Pro-Gly	1.05	LYS31, GLU37, ASP38, HIS34, THR27, GLN76, ASN33, PRO389, ARG393, PHE390	-174.37733	-42.16906	-132.20827

Gly-Pro-Leu-Gly-Leu-Leu-Gly-Phe-Leu-Gly-Pro-Leu-Gly-Leu-Ser	1.41	GLU35, ARG393, ASP38, HIS34, ASN33, PRO389, PHE390, THR27, LEU391, LEU39	-135.22308	-66.13282	-69.09026
Val-Ile-Glu-Lys-Tyr-Pro	0.75	LYS353, ASP30, LYS31, HIS34, GLU35	-135.07714	-24.27743	-110.79971
Asp-Glu-Asn-Ser-Lys-Phe	0.74	LYS353, LYS31, ASP30, HIS34, GLU35, LEU39	-125.50169	-34.14301	-91.35869
Met-Ile-Phe-Pro-Gly-Ala-Gly-Gly-Pro-Glu-Leu	1.09	LYS31, GLU37, HIS34, ASP38, LEU39, THR27	-125.2866	-30.64579	-94.64081
Val-Glu-Cys-Tyr-Gly-Pro-Asn-Arg-Pro-Gln-Phe	1.31	GLU35, LYS353, ASP38, ASP30, LYS31, GLN76	-111.08386	-39.69438	-71.38949
Met-Glu-Gly-Ala-Gln-Glu-Ala-Gln-Gly-Asp	1.04	LYS26, LYS31, ASP38, HIS34, THR27, LYS68, LYS353	-99.4708	-44.94334	-54.52746
Val-Met-Asp-Lys-Pro-Gln-Gly	0.77	GLU35, ASP30, LYS353, HIS34, LYS31, _GLU37	-99.04573	-30.31308	-68.73265
Tyr-Tyr-Ala-Pro-Phe-Asp-Gly-Ile-Leu	1.06	ARG393, LYS353, ASP38, GLU35, GLY354, ASN33, PRO389, HIS34, LYS31	-98.35432	-41.71706	-56.63726
His-Glu-Arg-Asp-Pro-Thr-His-Ile-Lys-Trp-Gly-Asp	1.49	ARG393, LYS353, LYS31, PHE390, THR27, LEU39, PRO389	-91.90103	-46.05346	-45.84758
Arg-Leu-Ser-Gly-Gln-Thr-Ile-Glu-Val-Thr-Ser-Glu-Tyr-Leu-Phe-Arg-His	2.04	LYS353, LYS31, GLU37, ASP38, GLN42, TYR83, GLN24, ARG357, HIS34, PHE327	-89.33021	-72.84742	-16.48279

*Results show the 30 peptides with highest CDocker energy (Scores) target to ACE2 (domain for Covid-19 spike receptor, 6M0J) from Table S1.

Table S3 Interaction counts, residues and energy of the peptides inhibiting the COVID-19 virus M^{pro}.*

Peptides	Molecular Weight (KDa)	Interaction Residues	Non-covalent Interaction		
			Total Interaction Energy (kcal/mol)	Total VDW Interaction Energy (kcal/mol)	Total Electrostatic Interaction Energy (kcal/mol)
Ile-Val-Gly-Arg-Pro-Arg	0.70	HIS41, MET49, PHE140, ASN142, HIS163, HIS164, MET165, GLU166, GLN189	-366.21436	-52.81027	-313.40409
Phe-Gln-Lys-Pro-Lys-Arg	0.80	THR26, PHE140, GLY143, MET165, GLU166, ASP187, GLN189, THR190	-308.37838	-59.35911	-249.01927
Tyr-Asn-Lys-Leu	0.54	HIS41, PHE140, LEU141, ASN142, MET165, GLU166, GLN189	-306.44818	-54.43838	-252.00979
Met-Arg-Trp-Arg-Asp	0.76	HIS41, PHE140, ASN142, CYS145, HIS164, GLU166	-286.85337	-53.53641	-233.31695
His-Trp-Thr-Thr-Gln-Arg	0.83	THR26, LEU27, PHE140, ASN142, CYS145, MET165, GLU166, LEU167 PRO168, HIS172, GLN189, GLN192	-274.87816	-73.98887	-200.8893
Lys-Leu-Lys-Phe-Val	0.63	LEU27, HIS41, GLY143, LEU141, SER144, CYS145, HIS163, MET165, GLU166, GLN189	-270.66224	-57.62319	-213.03905
Leu-Arg-Ile-Pro-Val-Ala	0.67	HIS41, MET49, PHE140, ASN142, CYS145, HIS163, HIS164 MET165, GLU166, LEU167, PRO168, GLN189	-263.5587	-62.7658	-200.7929

Tyr-Lys-Tyr-Tyr	0.64	MET49, PHE140, LEU141, SER144, CYS145, HIS163, MET165, GLU166, ASP187	-239.83012	-57.70609	-182.12403
Lys-Asp-Tyr-Arg-Leu	0.69	GLY143, SER144, CYS145, HIS164, MET165, GLU166, LEU167, PRO168, ARG188, GLN189	-239.13404	-48.9994	-190.13464
Tyr-Val-Val-Phe-Lys	0.65	MET49, PHE140, GLY143, MET165, GLU166, PRO168, LEU167, GLN189, THR190	-238.71907	-49.52335	-189.19571
Lys-Leu-Tyr-Met-Arg-Pro	0.81	THR26, HIS41, MET49, PHE140, GLY143, CYS145, HIS163, HIS164, MET165, GLU166, LEU167, PRO168, GLN189:	-235.05273	-71.74284	-163.30988
Leu-Lys-Pro-Asn-Met	0.60	LEU141, ASN142, GLY143, CYS145, HIS163, GLU166, GLN189:	-225.43308	-50.10753	-175.32554
Ala-Ile-Tyr-Lys	0.49	HIS41, ASN142, SER144, CYS145, HIS164, GLU166, LEU167, PRO168	-223.44699	-41.18654	-182.26046
Ile-Lys-Pro-Leu-Asn-Tyr	0.75	HIS41, THR26, PHE140, HIS164 MET165, GLU166, PRO168, GLN189, THR190, ALA191, GLN192	-218.76625	-64.2948	-154.47145
Thr-Gln-Val-Tyr	0.51	HIS41, MET49, GLY143, CYS145, HIS164, MET165, GLU166, GLN189	-201.01855	-44.28236	-156.73619

Lys-Phe-Tyr-Gly	0.51	MET49, PHE140, ASN142 HIS163, MET165, GLU166, GLN189, THR190	-183.87152	-42.62191	-141.24962
Asp-Glu-Asn-Ser-Lys-Phe	0.74	HIS41, ASN142, GLY143, CYS145, HIS164, GLU166, ASP187, GLN189, THR190	-181.25431	-59.62864	-121.62567
Leu-Ile-Val-Thr-Gln	0.57	PHE140, LEU141, SER144, CYS145, HIS163, HIS164, MET165, GLU166, GLN189	-177.30849	-46.52093	-130.78757
Asn-Asn-Asn-Pro-Phe-Lys-Phe	0.88	THR26, HIS41, ASN142, CYS145, HIS163, HIS164, MET165, GLU166, ARG188, GLN189, THR190, ALA191	-176.67979	-60.63914	-116.04065
Thr-Tyr-Leu-Gly-Ser	0.54	HIS41, MET49, PHE140, GLY143, CYS145, HIS163, HIS164, MET165, GLU166, GLN189	-166.27914	-47.43856	-118.84058
Val-Ile-Glu-Lys-Tyr-Pro	0.75	THR25, HIS41, MET49, ASN142, SER144, CYS145, MET165, GLU166, PRO168, GLN189	-162.41476	-47.68632	-114.72844
Asn-Trp-Gly-Pro-Leu-Val	0.68	LEU27, HIS41, MET49, ASN142, GLY143, CYS145, HIS163, HIS164, MET165, GLU166, GLN189	-146.84581	-59.68022	-87.16558
Val-Met-Asp-Lys-Pro-Gln-Gly	0.77	HIS41, ASN142, GLY143, HIS163, HIS164, GLU166, LEU167, PRO168, GLN189	-144.03871	-52.10908	-91.92963

Ile-Trp-His-His-Thr	0.69	HIS41, MET49, ASN142, GLY143, CYS145, HIS163, MET165, GLU166, ARG188, GLN189	-140.81248	-65.66636	-75.14612
Asp-Ile-Gly-Tyr-Tyr	0.63	THR24, MET49, GLY143, SER144, CYS145, ET165, GLU166, PRO168, ASP187, GLN189	-125.82687	-59.33094	-66.49592
Gly-Val-His-His-Ala	0.52	LEU141, GLY143, CYS145 MET165, GLU166, ASP187 ARG188, GLN189	-117.34984	-48.36107	-68.98877
Tyr-Leu-Ala-Gly-Asn-Gln	0.67	SER46, MET49, ASN142, HIS164, MET165, GLU166, PRO168, GLN189	-110.46483	-56.58424	-53.88059
Val-Ser-Gly-Ala-Gly-Arg-Tyr	0.71	HIS41, PHE140, LEU141, SER144, CYS145, HIS163, HIS164, MET165, GLU166, LEU167, PRO168, THR190	-101.72858	-57.68198	-44.04661
Asp-Tyr-Gly-Leu-Tyr-Pro	0.73	MET49, ASN142, MET165, GLU166, PRO168, ASP187, ARG188, GLN189	-80.6193	-49.3429	-31.2764
Asp-Tyr-Val-Gly-Asn	0.57	HIS41, MET49, PHE140, ASN142, GLY143, SER144, CYS145, HIS163, HIS164, MET165 GLU166, PRO168, GLN189, THR190	-64.27936	-52.46305	-11.81631

*Results show the 30 peptides with highest CDocker energy (Scores) target to M^{pro} (6LU7) from Table S1.

Table S4 Interaction counts, residues and energy of the peptides inhibiting the COVID-19 virus RdRp.*

Peptides	Molecular Weight (KDa)	Interaction Residues	Non-covalent bond		
			Total Interaction Energy (kcal/mol)	Total VDW Interaction Energy (kcal/mol)	Total Electrostatic Interaction Energy (kcal/mol)
Asp-Glu-Asn-Ser-Lys-Phe	0.74	MG1, MG2, A11, U10, U20, ASP452, LYS545, LYS551, ARG553, ARG555, LYS621, ASP623, LYS798	-870.28747	-44.73179	-825.55568
Ile-Ala-Glu	0.33	MG1, MG2, U20, LYS551, ARG553, ARG555, LYS621, CYS622	-737.55523	-14.8947	-722.66053
Ile-Val-Val-Glu	0.46	MG1, MG2, A19, U20, LYS551, ARG624, ARG553, LYS621, CYS622, ARG624, SER682	-613.53135	-25.86384	-587.66751
Lys-Leu-Pro-Ala-Gly-Thr-Leu-Phe	0.85	MG1, MG2, A11, U10, A19, U20, ASP452, LYS551, ARG553, LYS621, ASP623, SER759, LYS798	-590.98988	-63.0242	-527.96568
Thr-Gln-Val-Tyr	0.51	MG1, MG2, A11, U10, U20, LYS551, ARG553, LYS621, CYS622, ASP623, SER682, ASP684	-588.04935	-41.32527	-546.72408
Met-Arg-Trp-Arg-Asp	0.76	MG1, MG2, U18, A19, U20, U10, A11 U12, ASP452, LYS551, ARG553, THR556, LYS621, ASP623, SER759	-563.53801	-55.12032	-508.41769
Asp-Phe-Gly	0.34	MG1, MG2, U10, U20, LYS551, ARG553, ARG555, PRO620, LYS621, CYS622, ASP623	-559.43939	-15.22035	-544.21904
Val-Ile-Glu-Lys-	0.75	MG1, MG2, U18, A19, U20, LYS551,	-556.01799	-53.81719	-502.2008

Tyr-Pro		ARG553, ARG555, LYS621, ARG624, SER682, SER814			
Phe-Lys-Gly-Arg-Tyr-Tyr-Pro	0.93	MG1, A11, U10, U18, A19, U20, LYS551, ARG553, LYS621, ASP623, ASP684, LYS798	-551.53861	-67.64523	-483.89338
Asp-Tyr-Val-Gly-Asn	0.57	MG1, MG2, A19, U20, U10, LYS551, ARG553, PRO620, LYS621, CYS622, ARG624, ASN691	-528.40075	-41.27113	-487.12962
Gly-Gly-Val-Ile-Pro-Asn	0.56	MG1, MG2, A19, U20, LYS551, ARG553, ARG555, LYS621, ASP623, SER682, ASP760	-518.09597	-33.40101	-484.69497
Ala-Pro-Gly-Ala-Gly-Val-Tyr	0.63	MG1, MG2, A11, U10, YS551, ARG553, ARG555, LYS621, ASP623, SER682, ASP684	-509.1754	-44.55624	-464.61916
Lys-Pro-Pro-Glu-Thr-Val	0.67	MG1, MG2, A11, U10, LYS551, ARG553, ARG555, CYS622, ASP623, THR680,	-493.8504	-42.42373	-451.42667
Asp-Ile-Gly-Tyr-Tyr	0.63	MG1, MG2, LYS545, LYS551, ARG553, ARG555, THR556, ARG624, THR680, ASN691	-483.6117	-44.51806	-439.09364
Val-Met-Asp-Lys-Pro-Gln-Gly	0.77	MG1, MG2, U18, A19, U20, ASP452, LYS551, LYS621, CYS622, ASP623, ARG553, THR556	-464.82911	-49.75556	-415.07355
Tyr-Lys-Tyr-Tyr	0.64	MG1, MG2, A11, U10, U20, ASP452, LYS551, ARG553, ARG555, THR556, LYS621, ASP623, ASP760	-442.09392	-44.16422	-397.92971

Leu-Ile-Val-Thr-Gln	0.57	MG1, MG2, A11, U10, A19, U20, LYS551, ARG553, LYS62, CYS622, ARG624	-437.07126	-40.80698	-396.26428
Ile-Ala-Tyr-Lys-Pro-Ala-Gly	0.72	MG1, MG2, U10, U18, A19, U20, LYS551, LYS621, CYS622, ASP623, SER759, ASP760	-424.93775	-54.41667	-370.52108
Ile-Lys-Pro-Leu-Asn-Tyr	0.75	MG1, MG2, A11, U10, U20, ASP452, LYS551, ARG555, THR556, ASP623, THR680, LYS798, TYR456	-424.60092	-45.90159	-378.69933
Lys-Asp-Tyr-Arg-Leu	0.69	MG1, MG2, A11, U10, U20, ASP452, ILE548, LYS551, ARG555, THR556, ASP623, ASP760	-405.73027	-46.42355	-359.30672
Asp-Tyr-Gly-Leu-Tyr-Pro	0.73	MG1, MG2, U20, LYS551, ARG553, ARG555, THR556, TYR619, LYS621, ARG624, SER682	-394.1501	-45.26762	-348.88248
Val-Ser-Gly-Ala-Gly-Arg-Tyr	0.71	MG1, MG2, A19, U20, A11, U10, LYS551, ARG553, ARG555, ASP618, LYS621, ASP623, GLU811	-392.18258	-45.26009	-346.92249
Tyr-Leu-Ala-Gly-Asn-Gln	0.67	MG1, MG2, U20, U12, LYS551, ARG553, ARG555, LYS621, CYS622, ASP623, ASP760	-376.36536	-47.56956	-328.7958
Ile-Trp-His-His-Thr	0.69	MG1, MG2, A11, U10, ASP452, LYS551, ARG553, VAL557, ASP623, LYS798	-349.45456	-48.04922	-301.40534
Thr-Tyr-Leu-Gly-Ser	0.54	MG1, MG2, A11, U10, ASP452, ARG553, THR556, PRO620, LYS621,	-349.33547	-44.58196	-304.75351

		ASP623, SER682			
Phe-Val-Asn-Pro-Gln-Ala-Gly-Ser	0.82	MG1, MG2, A11, U10, LYS551, ARG553, ARG555, ASP623, ASP623, ARG836	-346.05993	-50.36526	-295.69467
Asn-Trp-Gly-Pro-Leu-Val	0.68	MG1, MG2, A11, ASP452, LYS551, THR556, PRO620, LYS621, ASP623, SER682, LYS798	-342.11034	-41.62035	-300.48999
His-Trp-Thr-Thr-Gln-Arg	0.83	MG1, MG2, A11, U18, A19, U20, LYS551, LYS621, CYS622, ASP623, THR687	-325.04129	-63.24914	-261.79215
Asn-Asn-Asn-Pro-Phe-Lys-Phe	0.88	MG1, MG2, U10, U18, A19, U20, ASP623, ASP760, ASP761, SER814	-230.30088	-56.8155	-173.48537
Tyr-Val-Val-Phe-Lys	0.65	MG1, MG2, A19, U20, LYS551, LYS621, ASP623, ASP760, ASP761, SER814	-213.13233	-52.588	-160.54433

*Results show the 30 peptides with highest CDocker energy (Scores) target to COVID-19 virus RdRp (7BV2) from Table S1.

Table S5 Interaction between reported drugs, Covid-19 virus Spike protein residues and ACE2. *

Drugs	*Interacting group*	*Distance (Å)*	*Types*	*Interaction Counts*
MLN-4760	A:LYS31:HZ3 - MLN-4760: N4	2.11892	H-bond	6
	A:LYS353:HZ1 - MLN-4760:O19	1.76768	H-bond	
	MLN-4760:H32 - A:GLU35:OE1	2.56052	H-bond	
	MLN-4760:Cl21 - A:LYS31	4.52192	Alkyl	
	A:HIS34 - MLN-4760	3.9847	Pi-Alkyl	
	A:HIS34 - MLN-4760:C26	4.79819	Pi-Alkyl	
Covid-19 virus Spike protein residues (Short as Sp)	SP:LYS417:NZ - A:ASP30:OD2	2.9049	Salt Bridge	19
	A:LYS31:NZ - SP:GLU484:OE1	4.39378	Attractive Charge	
	A:TYR41:OH - SP:THR500:OG1	2.70747	H-bond	
	A:GLN42:NE2 - SP:GLY446:O	3.24366	H-bond	
	A:GLN42:NE2 - SP:TYR449:OH	2.78751	H-bond	
	A:TYR83:OH - SP:ASN487:OD1	2.78788	H-bond	
	A:LYS353:NZ - SP:GLY496:O	3.084	H-bond	
	SP:TYR449:OH - A:ASP38:OD2	2.69516	H-bond	
	SP:ASN487:ND2 - A:GLN24:OE1	2.68768	H-bond	
	SP:GLN493:NE2:B - A:GLU35:OE1	3.13204	H-bond	
	SP:GLY502:N - A:LYS353:O	2.78374	H-bond	
	A:HIS34:CD2 - SP:TYR453:OH	2.86041	H-bond	
	A:TYR83:OH - SP:PHE486	4.08594	Pi-Donor H-Bond	
	SP:LEU455:CD1 - A:HIS34	3.84933	Pi-Sigma	
	A:TYR83 - SP:PHE486	5.14407	Pi-Pi Stacked	
	A:LYS353:C,O;GLY354:N - SP:TYR505	4.00384	Amide-Pi Stacked	
	SP:PHE486 - A:MET82	4.74448	Pi-Alkyl	
	SP:TYR489 - A:LYS31	4.75224	Pi-Alkyl	
	SP:TYR505 - A:LYS353	4.62094	Pi-Alkyl	

* In A:XXX123:HZ3 - MLN-4760: N4, A represents the receptor chain, XXX represents the abbreviation of amino acid molecule, 123 represents the serial number of this amino acid residue molecule of receptor, H represents the interacting atom of this amino acid residue, Z3 represents serial number of this aton in the residues; MLN-4760 is the ligand meolecule, N represents the interacting atom of MLN-4760, 4 represents serial number of this aton in theMLN-4760.

Table S6 Interaction mechanism between reported drugs and COVID-19 virus M^{pro}.

Drugs	*Interacting group*	*Distance (Å)*	*Types*	*Interaction Counts*
Inhibition N3 (Short as N3)	A:GLY143:HN - N3:0106:O	1.92616	H-bond	23 Non-covalent bond; 1 Covalent bond
	A:GLU166:HN - N3:VAL3:O	1.93628	H-bond	
	N3:ALA2:HN - A:THR190:O	1.79854	H-bond	
	N3:VAL3:HN - A:GLU166:O	1.79536	H-bond	
	N3:LEU4:HN - A:GLN189:OE1	1.91794	H-bond	
	N3:PJE5:H8 - A:PHE140:O	2.38037	H-bond	
	N3:PJE5:H8 - A:GLU166:OE2	2.7507	H-bond	
	N3:PJE5:H9 - A:HIS163:NE2	1.73845	H-bond	
	N3:PJE5:H10 - A:HIS164:O	2.176	H-bond	
	A:MET165:HA - N3:VAL3:O	2.72823	H-bond	
	A:MET165:HA - N3:PJE5:N5	2.68266	H-bond	
	A:GLN189:HA - N3:ALA2:O	2.48559	H-bond	
	N3:ALA2:HA - A:GLU166:O	2.64181	H-bond	
	N3:VAL3:HA - A:GLN189:OE1	2.82411	H-bond	
	N3:LEU4:HA - A:HIS164:O	2.84778	H-bond	
	N3:PJE5:H7 - A:ASN142:OD1	2.62311	H-bond	
	A:LEU141:C,O;ASN142:N - N3:PJE5	4.14249	Amide-Pi Stacked	
	A:MET49 - N3:LEU4	4.73045	Alkyl	
	N3:ALA2 - A:MET165	4.57008	Alkyl	
	N3:ALA2 - A:LEU167	5.46094	Alkyl	
	A:HIS41 - N3:LEU4	4.26542	Pi-Alkyl	
	N3:02J1 - A:PRO168	4.84869	Pi-Alkyl	
	N3:02J1 - A:ALA191	4.53889	Pi-Alkyl	
	**CYS145:SG – N3:PJE5:C20**	**1.76519**	**Covalent Bond**	
Ritonavir	A:GLU166:HN - Ritonavir: O13	1.97035	H-bond	14
	A:GLU166:HN - Ritonavir: O28	2.87207	H-bond	
	**Ritonavir: H56 - A:GLN189:OE1**	**1.98223**	**H-bond**	
	A:GLY143:HA1 - Ritonavir: O25	2.94655	H-bond	
	A:MET165:HA - Ritonavir: O13	2.87353	H-bond	
	Ritonavir: H77 - A:THR190:O	2.89883	H-bond	
	Ritonavir: H80 - A:THR190:O	2.47189	H-bond	
	Ritonavir:H82 - A:THR26:O	2.56857	H-bond	

	**A:CYS145:SG - Ritonavir**	**5.08306**	**Pi-Sulfur**	
	A:ASN142:C,O;GLY143:N - Ritonavir:	3.80822	Amide-Pi Stacked	
	Ritonavir:C33 - A:MET165	4.88587	Alkyl	
	Ritonavir:C46 - A:PRO168	4.4653	Alkyl	
	Ritonavir - A:MET49	4.64556	Pi-Alkyl	
	Ritonavir - A:PRO168	4.61105	Pi-Alkyl	
Lopinavir	Lopinavir::H80 - A:HIS163:NE2	2.11133	H-bond	15
	Lopinavir::H54 - A:GLN189:OE1	2.65	H-bond	
	Lopinavir::H55 - A:GLN189:OE1	2.62608	H-bond	
	Lopinavir::H55 - Lopinavir::O37	2.76231	H-bond	
	Lopinavir::H58 - A:LEU141:O	2.5251	H-bond	
	Lopinavir::H63 - A:GLU166:OE1	2.70252	H-bond	
	Lopinavir::H82 - Lopinavir::O37	2.44415	H-bond	
	A:GLU166:OE1 - Lopinavir:	4.7799	Pi-Anion	
	A:GLY143:HN - Lopinavir	3.01133	Pi-Donor Hydrogen Bond	
	Lopinavir::H68 - A:HIS41	2.83522	Pi-Sigma	
	A:CYS145:SG - Lopinavir	4.91915	Pi-Sulfur	
	A:HIS41 - Lopinavir	5.47488	Pi-Pi T-shaped	
	Lopinavir::C26 - A:CYS145	4.64695	Alkyl	
	Lopinavir::C27 - A:MET165	4.56785	Alkyl	
	Lopinavir: - A:MET165	4.38108	Pi-Alkyl	

Table S7 Interaction mechanism between reported drugs and COVID-19 virus RdRp.

Drugs	*Interacting group*	*Distance (Å)*	*Types*	*Interaction Counts*
Remdesivir (short as RMP)	D:MG1:MG - P:RMPH47:H56	3.55564	Attractive Charge	10 Non-covalent bond; 1 Covalent bond
	D:MG2:MG - P:RMP101:O5	3.92852	Attractive Charge	
	P:RMP101:N5 - P:U20:O4	3.2051	H-bond	
	P:RMP101:N5 - T:U10:O4	3.1925	H-bond	
	T:U10:N3 - P:RMP101:N4	3.03083	H-bond	
	P:RMP101:C6 - A:ASP760:OD2	2.99026	H-bond	
	P:U20 - P:RMP101	4.72649	Pi-Pi Stacked	
	P:RMP101 - P:U20	4.03164	Pi-Pi Stacked	
	P:RMP101 - T:A11	4.08375	Pi-Pi Stacked	
	P:RMP101 - T:A11	5.67343	Pi-Pi Stacked	
	**U20:O3' - RMP101:P1**	**1.71622**	**Covalent Bond**	
Ribavirin	A:GLU166:HN - Ritonavir: O13	1.97035	H-bond	14
	A:GLU166:HN - Ritonavir: O28	2.87207	H-bond	
	**Ritonavir: H56 - A:GLN189:OE1**	**1.98223**	**H-bond**	
	A:GLY143:HA1 - Ritonavir: O25	2.94655	H-bond	
	A:MET165:HA - Ritonavir: O13	2.87353	H-bond	
	Ritonavir: H77 - A:THR190:O	2.89883	H-bond	
	Ritonavir: H80 - A:THR190:O	2.47189	H-bond	
	Ritonavir:H82 - A:THR26:O	2.56857	H-bond	
	**A:CYS145:SG - Ritonavir**	**5.08306**	**Pi-Sulfur**	
	A:ASN142:C,O;GLY143:N - Ritonavir:	3.80822	Amide-Pi Stacked	
	Ritonavir:C33 - A:MET165	4.88587	Alkyl	
	Ritonavir:C46 - A:PRO168	4.4653	Alkyl	
	Ritonavir - A:MET49	4.64556	Pi-Alkyl	
	Ritonavir - A:PRO168	4.61105	Pi-Alkyl	
Favipiravir	Lopinavir::H80 - A:HIS163:NE2	2.11133	H-bond	15
	Lopinavir::H54 - A:GLN189:OE1	2.65	H-bond	
	Lopinavir::H55 - A:GLN189:OE1	2.62608	H-bond	
	Lopinavir::H55 - Lopinavir::O37	2.76231	H-bond	
	Lopinavir::H58 - A:LEU141:O	2.5251	H-bond	
	Lopinavir::H63 - A:GLU166:OE1	2.70252	H-bond	
	Lopinavir::H82 - Lopinavir::O37	2.44415	H-bond	

A:GLU166:OE1 - Lopinavir:	4.7799	Pi-Anion
A:GLY143:HN - Lopinavir	3.01133	Pi-Donor Hydrogen Bond
Lopinavir::H68 - A:HIS41	2.83522	Pi-Sigma
A:CYS145:SG - Lopinavir	4.91915	Pi-Sulfur
A:HIS41 - Lopinavir	5.47488	Pi-Pi T-shaped
Lopinavir::C26 - A:CYS145	4.64695	Alkyl
Lopinavir::C27 - A:MET165	4.56785	Alkyl
Lopinavir: - A:MET165	4.38108	Pi-Alkyl

Table S8 Interaction mechanism between screening peptides and the ACE2 .*

Peptides	*Interaction*	*Distance (Å)*	*Types*	*Interaction Counts*
Ile-Val-Gly-Arg-Pro-Arg-His-Gln-Gly (Abbreviated to PA1)	PA1: H3 - A:ASP38:OD2	1.94851	Salt Bridge; Attractive Charge	10
	PA1:H63 - A:GLU37:OE2	2.6123	Salt Bridge; Attractive Charge	
	PA1:H100 - A:GLU35:OE2	2.71804	Salt Bridge; Attractive Charge	
	A:LYS31:NZ - PA1:O148	2.90642	Attractive Charge	
	PA1:N1 - A:GLU35:OE2	5.28968	Attractive Charge	
	PA1:H4 - A:GLU35:OE1	2.29616	H-bond	
	PA1:H65 - A:HIS34:O	2.40955	H-bond	
	PA1:H66 - A:HIS34:O	2.70468	H-bond	
	A:PHE28:HA - PA1:O135	2.77846	H-bond	
	A:LYS31:HE1 - PA1:O140	2.95593	H-bond	
Phe-Gln-Lys-Pro-Lys-Arg (Abbreviated to PA2)	PA2:H95 - A:ASP38:OD2	1.98822	Salt Bridge; Attractive Charge	12
	PA2:N1 - A:ASP30:OD2	2.95322	Attractive Charge	
	PA2:N92 - A:GLU35:OE2	5.00577	Attractive Charge	
	PA2:N114 - A:GLU37:OE2	2.96477	Attractive Charge	
	PA2:H4 - A:ASP30:OD1	2.29513	H-bond	
	PA2:H37 - A:THR27:O	2.93469	H-bond	
	PA2:H93 - A:GLU35:OE1	2.18778	H-bond	
	PA2:H118 - A:GLU37:OE2	2.11005	H-bond	
	A:LYS31:HA - PA2:O34	3.00994	H-bond	
	A:HIS34:HD2 - PA2:O121	2.65761	H-bond	
	A:HIS34:HD2 - PA2:O122	2.49258	H-bond	
	PA2:H91 - A:GLU35:OE1	2.79475	H-bond	
Gly-Asp-Leu-Gly-Lys-Thr-Thr-Thr-Val-Ser-Asn-Trp-Ser-Pro-Pro-Lys-Tyr-Lys-Asp-Thr-Pro	PA3:H2 - A:GLU37:OE2	2.16467	Salt Bridge; Attractive Charge	16
	PA3:N64 - A:ASP30:OD2	2.67404	Attractive Charge	
	A:LYS31:HZ2 - PA3:O187	2.33302	H-bond	
	A:LYS31:HZ3 - PA3:O176	1.7755	H-bond	
	A:LYS353:HZ1 - PA3:O111	2.21185	H-bond	
	A:PHE390:HN - PA3:O9	2.97158	H-bond	
	PA3:H77 - A:ALA387:O	3.09668	H-bond	
	PA3:H105 - A:GLU37:OE2	2.26274	H-bond	
	PA3:H150 - A:GLU35:OE1	2.09259	H-bond	
	A:HIS34:HA - PA3:O104	2.66704	H-bond	
	A:LYS353:HE2 - PA3:O111	2.52645	H-bond	
	PA3:H25 - A:ALA387:O	2.39437	H-bond	
	PA3:N1 - A:HIS34	4.35511	Electrostatic	
	A:HIS34 - PA3	3.98427	Hydrophobic	

(Abbreviated to PA3)	A:HIS34 - PA3	3.63477	Hydrophobic	
	PA3:C35 - A:PRO389	4.81983	Hydrophobic	
Pro-Thr-His-Ile-Lys-Trp-Gly-Asp (Abbreviated to PA4)	A:LYS68:HZ2 - PA4:O129	2.00227	Salt Bridge; Attractive Charge	17
	PA4:H3 - A:ASP30:OD2	2.12252	Salt Bridge; Attractive Charge	
	PA4:H84 - A:ASP38:OD2	2.17334	Salt Bridge; Attractive Charge	
	PA4:N83 - A:GLU35:OE2	5.28958	Attractive Charge	
	A:LYS31:HZ3 - PA4:O66	1.86	H-bond	
	PA4:H2 - A:ASP30:OD1	2.02314	H-bond	
	PA4:H18 - A:ASP30:OD1	2.16127	H-bond	
	PA4:H24 - A:ASP30:O	2.47484	H-bond	
	PA4:H86 - A:GLU35:OE1	2.54447	H-bond	
	PA4:H90 - A:GLU35:OE2	2.37627	H-bond	
	PA4:H70 - A:GLU35:OE2	2.43157	H-bond	
	PA4:H81 - A:ASP38:OD2	2.47518	H-bond	
	A:LYS31:HZ1 - PA4	2.43541	Pi-Cation; Pi-Donor H-bond	
	A:GLU35:OE2 - PA4	3.269	Pi-Anion	
	A:GLU35:OE2 - PA4	3.7227	Pi-Anion	
	A:HIS34 - PA4	4.56624	Pi-Pi T-shaped	
	PA4:C54 - A:LYS31	5.30883	Alkyl	
Lys-Glu-Asp-Asp-Glu-Glu-Glu-Glu-Gln-Glu-Glu-Glu (Abbreviated to PA5)	A:LYS31:HZ1 - PA5: O49	2.01721	Salt Bridge; Attractive Charge	15
	A:LYS31:HZ2 - PA5:O76	1.97737	Salt Bridge; Attractive Charge	
	A:LYS353:NZ - PA5:O91	3.01341	Attractive Charge	
	A:ARG559:NE - PA5:O186	4.53243	Attractive Charge	
	PA5:N19 - A:GLU35:OE2	5.07936	Attractive Charge	
	A:LYS31:HZ1 - PA5:O63	2.68919	H-bond	
	A:ARG559:HH21 - PA5:O186	2.13148	H-bond	
	PA5:H157 - A:ALA387:O	2.98983	H-bond	
	A:LYS31:HE1 - PA5:O49	2.89315	H-bond	
	A:HIS34:HD2 - PA5:O123	2.98101	H-bond	
	A:LYS353:HA - PA5:O120	3.07609	H-bond	
	A:LYS353:HE1 - PA5:O120	2.55082	H-bond	
	A:ALA387:HA - PA5:O168	2.78271	H-bond	
	PA5:H67 - A:GLU35:OE2	2.66516	H-bond	
	PA5:H174 - A:ALA387:O	2.66012	H-bond	

*Results show the 5 peptides with highest Total interaction energy from Table 2

Table S9 Interaction mechanism between screening peptides and the COVID-19 virus M^{pro}.*

Peptides	*Interaction*	*Distance (Å)*	*Types*	*Interaction Counts*
Ile-Val-Gly-Arg-Pro-Arg (Abbreviated to PM1)	PM1: N61 - A:GLU166:OE2	3.64703	Attractive Charge	19
	PM1:N99 - A:GLU166:OE2	3.30671	Attractive Charge	
	A:GLU166:HN - PM1:O21	2.28661	H-bond	
	A:GLU166:HN - PM1:O37	2.56828	H-bond	
	PM1:H4 - A:HIS164:O	1.88095	H-bond	
	PM1:H23 - A:GLN189:OE1	2.0108	H-bond	
	PM1:H59 - A:ASN142:OD1	2.97247	H-bond	
	PM1:H63 - A:GLU166:OE1	1.96391	H-bond	
	PM1:H65 - A:PHE140:O	2.12258	H-bond	
	PM1:H66 - A:GLU166:OE2	2.02788	H-bond	
	PM1:H103 - A:PHE140:O	2.65029	H-bond	
	PM1:H103 - A:GLU166:OE2	2.44563	H-bond	
	PM1:H104 - A:HIS163:NE2	2.87107	H-bond	
	A:MET165:HA - PM1:O21	2.26398	H-bond	
	PM1:N1 - A:HIS41	4.49594	Pi-Cation	
	PM1:C9 - A:MET49	3.53167	Alkyl	
	PM1:C32 - A:MET165	3.57556	Alkyl	
	A:HIS41 - PM1:C9	4.16457	Pi-Alkyl	
	A:HIS41 - PM1:C16	4.4944	Pi-Alkyl	
Phe-Gln-Lys-Pro-Lys-Arg (Abbreviated to PM2)	PM2:H59 - A:GLU166:OE2	1.86885	Salt Bridge; Attractive Charge	10
	A:GLY143:HN - PM2:O121	2.13224	H-bond	
	PM2:H3 - A:THR190:O	1.92953	H-bond	
	PM2:H58 - A:PHE140:O	1.79836	H-bond	
	PM2:H77 - A:GLN189:OE1	1.96272	H-bond	
	PM2:H94 - A:ASP187:O	2.03056	H-bond	
	PM2:H119 - A:THR26:O	1.79572	H-bond	
	A:GLY143:HA1 - PM2:O122	2.53776	H-bond	
	PM2:H67 - A:GLN189:OE1	2.42272	H-bond	
	PM2 - A:MET165	4.51996	Pi-Alkyl	
Tyr-Asn-Lys-Leu (Abbreviated to PM3)	PM3:H57 - A:GLU166:OE2	2.15489	Salt Bridge; Attractive Charge	11
	A:GLU166:HN - PM3:O23	2.00926	H-bond	
	PM3:H3 - A:GLU166:O	1.907	H-bond	
	PM3:H21 - A:GLU166:OE1	2.03261	H-bond	
	PM3:H25 - A:GLN189:OE1	2.12846	H-bond	
	PM3:H55 - PM3:O20	1.78198	H-bond	
	PM3:H56 - A:PHE140:O	1.89707	H-bond	

	A:HIS41:HD2 - PM3:O79	2.68909	H-bond	
	A:ASN142:HA - PM3:O59	2.58219	H-bond	
	A:MET165:HA - PM3:O23	2.66361	H-bond	
	PM3:H52 - A:LEU141:O	3.05206	H-bond	
Met-Arg-Trp-Arg-Asp (Abbreviated to PM4)	PM4:H86 - A:GLU166:OE1	2.42509	Salt Bridge; Attractive Charge	16
	PM4:N36 - A:GLU166:OE2	4.77933	Attractive Charge	
	A:GLU166:HN - PM4:O101	2.37075	H-bond	
	PM4:H21 - A:CYS145:SG	2.72862	H-bond	
	PM4:H21 - A:HIS164:O	2.70662	H-bond	
	PM4:H34 - A:PHE140:O	2.60278	H-bond	
	PM4:H34 - A:GLU166:OE2	2.60835	H-bond	
	PM4:H37 - A:ASN142:OD1	2.1775	H-bond	
	PM4:H40 - A:PHE140:O	2.93106	H-bond	
	PM4:H40 - A:GLU166:OE2	1.97429	H-bond	
	PM4:H89 - A:GLU166:O	1.90816	H-bond	
	A:ASN142:HA - PM4:O43	2.52998	H-bond	
	PM4:H6 - A:HIS164:O	2.4645	H-bond	
	PM4:H47 - A:ASN142:OD1	2.39291	H-bond	
	PM4:N1 - A:HIS41	4.69369	Pi-Cation	
	PM4:C14 - A:CYS145	4.41926	Alkyl	
His-Trp-Thr-Thr-Gln-Arg (Abbreviated to PM5)	A:GLN192:HN - PM5:O83	2.16811	H-bond	25
	PM5:H35 - A:THR26:O	2.08623	H-bond	
	PM5:H59 - A:GLN189:OE1	2.18449	H-bond	
	PM5:H65 - A:GLN189:OE1	2.09377	H-bond	
	PM5:H73 - A:GLU166:O	2.43214	H-bond	
	PM5:H86 - A:MET165:SD	2.46247	H-bond	
	PM5:H103 - A:GLU166:OE1	2.48411	H-bond	
	PM5:H106 - A:PRO168:O	1.97075	H-bond	
	PM5:H109 - A:GLU166:OE1	1.94799	H-bond	
	PM5:H110 - A:LEU167:O	2.61688	H-bond	
	PM5:H110 - A:PRO168:O	2.41148	H-bond	
	A:MET165:HA - PM5:O57	2.60098	H-bond	
	A:PRO168:HD2 - PM5:O83	2.39443	H-bond	
	A:HIS172:HD2 - PM5:N17	2.74009	H-bond	
	A:GLN189:HA - PM5:O71	3.0006	H-bond	
	PM5:H6 - A:ASN142:OD1	2.36018	H-bond	
	PM5:H16 - A:PHE140:O	2.49219	H-bond	
	PM5:H16 - A:GLU166:OE2	2.7702	H-bond	
	PM5:H47 - A:GLN189:OE1	2.81447	H-bond	
	PM5:H49 - A:HIS164:O	2.45424	H-bond	
	PM5:H63 - A:GLN189:OE1	2.59823	H-bond	
	PM5:H45 - A:HIS41	3.31117	H-bond (Pi-Donor)	

A:CYS145:SG - PM5	5.44422	Pi-Sulfur
A:CYS145:SG - PM5	5.70904	Pi-Sulfur
PM5 - A:LEU27	5.30182	Pi-Sulfur

*Results show the 5 peptides with highest Total interaction energy from Table 2.

Table S10 Interaction mechanism between screening peptides and the COVID-19 virus RdRp*

Peptides	*Interaction*	*Distance (Å)*	*Types*	*Interaction Counts*
Asp-Glu-Asn-Ser-Lys-Phe (Abbreviated to PR1)	A:ARG553:HH12 - PR1: O97	2.41968	Salt Bridge; Attractive Charge	23
	A:ARG553:HH22 - PR1:O97	2.81945	Salt Bridge; Attractive Charge	
	PR1:H3 - A:ASP618:OD2	2.08635	Salt Bridge; Attractive Charge	
	PR1:H4 - A:ASP618:OD2	2.38828	Salt Bridge; Attractive Charge	
	PR1:H73 - A:ASP623:OD2	1.90158	Salt Bridge; Attractive Charge	
	A:LYS551:NZ - PR1:O12	2.71162	Attractive Charge	
	D:MG2:MG - PR1:O27	2.32324	Attractive Charge	
	PR1:N1 - A:GLU811:OE1	4.81828	Attractive Charge	
	A:LYS551:HZ3 - PR1:O29	1.96312	H-bond	
	A:ARG553:HH22 - PR1:O76	2.70151	H-bond	
	A:CYS622:HN - PR1:O38	2.29641	H-bond	
	PR1:H52 - P:U20:O3'	1.9553	H-bond	
	PR1:H56 - A:ASP623:OD1	2.44019	H-bond	
	PR1:H72 - A:THR680:O	2.33369	H-bond	
	PR1:H74 - A:TYR456:OH	2.33397	H-bond	
	PR1:H50 - A:ASP760:OD2	2.6515	H-bond	
	PR1:H58 - A:ASP623:OD2	2.65037	H-bond	
	D:MG1:MG - PR1:O38	2.41282	Metal-Acceptor	
	D:MG1:MG - PR1:O43	2.20717	Metal-Acceptor	
	P:U20 - PR1	4.61919	Pi-Pi Stacked	
	T:A11 - PR1	5.60532	Pi-Pi Stacked	
	P:U20 - PR1	4.61919	Pi-Pi Stacked	
	T:A11 - PR1	5.60532	Pi-Pi Stacked	
Ile-Ala-Glu (Abbreviated to PR2)	PR2:H2 - A:ASP760:OD2	1.91332	Salt Bridge; Attractive Charge	12
	A:LYS551:NZ - PR2:O47	3.43924	Attractive Charge	
	A:ARG553:NE - PR2:O47	4.62578	Attractive Charge	
	D:MG1:MG - PR2:O44	2.36557	Attractive Charge; Metal-Acceptor	
	D:MG2:MG - PR2:O44	4.41751	Attractive Charge	
	A:ARG553:HH11 - PR2:O47	2.20739	H-bond	
	P:U20:HO3' - PR2:O43	2.21264	H-bond	
	PR2:H4 - P:U20:O3'	1.8496	H-bond	
	PR2:H6 - A:ASP760:OD2	2.69392	H-bond	

	D:MG1:MG - PR2:O21	2.90434	Metal-Acceptor	
	D:MG1:MG - PR2:O31	2.32222	Metal-Acceptor	
	D:MG2:MG - PR2:O43	2.40091	Metal-Acceptor	
Ile-Val-Val-Glu (Abbreviated to PR3)	PR3:H4 - P:U20:OP2	1.91985	Salt Bridge; Attractive Charge	18
	A:LYS551:NZ - PR3:O66	5.47077	Attractive Charge	
	D:MG1:MG - PR3:O66	2.3133	Attractive Charge; Metal-Acceptor	
	D:MG1:MG - PR3:O69	5.42222	Attractive Charge	
	D:MG2:MG - PR3:O69	2.50496	Attractive Charge	
	A:ARG553:HH12 - PR3:O65	1.89595	H-bond	
	P:U20:HO3' - PR3:O69	2.11714	H-bond	
	PR3:H3 - P:U20:O5'	2.58682	H-bond	
	P:U20:H5'1 - PR3:O69	2.70014	H-bond	
	P:U20:H2' - PR3:O21	2.70435	H-bond	
	P:U20:H3' - PR3:O21	2.34884	H-bond	
	PR3:H6 - P:U20:OP2	2.82406	H-bond	
	PR3:H41 - P:U20:O3'	2.50738	H-bond	
	D:MG1:MG - PR3:O53	2.19776	Metal-Acceptor	
	D:MG2:MG - PR3:O68	2.3325	Metal-Acceptor	
	PR3:C32 - A:ARG555	4.29544	Alkyl	
	PR3:C48 - A:CYS622	4.69943	Alkyl	
	P:U20 - PR3:C9	4.93808	Pi-Alkyl	
Lys-Leu-Pro-Ala-Gly-Thr-Leu-Phe (Abbreviated to PR4)	D:MG1:MG - PR4:O128	2.36297	Attractive Charge	16
	D:MG2:MG - PR4:O128	4.30656	Attractive Charge	
	A:LYS551:HZ3-PR4:O107	1.71639	H-bond	
	P:U20:HO3' - PR4:O74	1.91296	H-bond	
	PR4:H4 - T:U10:O4	2.80496	H-bond	
	PR4:H20 - P:U20:O2	2.53946	H-bond	
	PR4:H21 - A:SER759:OG	1.83105	H-bond	
	PR4:H21 - P:U20:O2'	2.26354	H-bond	
	PR4:H6 - T:A11:N1	2.55503	H-bond	
	PR4:H47 - A:THR556:O	3.07951	H-bond	
	PR4:H61 - A:ASP623:OD1	2.76345	H-bond	
	PR4:H71 - P:U20:O3'	2.35833	H-bond	
	PR4:H72 - A:ASP760:OD2	2.62081	H-bond	
	PR4:H80 - P:U20:OP2	2.58014	H-bond	
	D:MG1:MG - PR4:O67	2.25232	Metal-Acceptor	
	PR4:N1 - T:A11	3.72561	Pi-Cation	
	A:ALA547 - PR4:C34	4.05304	Alkyl	
	A:ARG555 - PR4:Molecule	4.96088	Alkyl	
	PR4:C34 - A:LYS545	4.31785	Alkyl	
	PR4:C38 - A:LYS545	5.00295	Alkyl	
	A:HIS439 - PR4:C98	5.26638	Pi-Alkyl	

	P:U20 - PR4:C38	4.87137	Pi-Alkyl	
Thr-Gln-Val-Tyr (Abbreviated to PR5)	D:MG1:MG - PR5:O71	4.30386	Attractive Charge	19
	D:MG2:MG - PR5:O71	2.3125	Attractive Charge; Metal-Acceptor	
	A:ARG553:HH22 - PR5:O28	2.58957	H-bond	
	A:THR687:HG1 - PR5:O9	2.12986	H-bond	
	P:U20:HO3' - PR5:O71	2.53737	H-bond	
	PR5:H4 - A:SER682:O	2.5108	H-bond	
	PR5:H31 - A:THR556:O	3.03792	H-bond	
	A:ARG555:HD1 - PR5:O67	2.73452	H-bond	
	A:SER682:HB1 - PR5:O16	2.37202	H-bond	
	A:SER682:HB2 - PR5:O16	2.66282	H-bond	
	P:U20:H2' - PR5:O33	2.58855	H-bond	
	P:U20:H3' - PR5:O33	2.82291	H-bond	
	PR5:H37 - P:U20:O3'	2.78801	H-bond	
	D:MG1:MG - PR5:O49	2.36053	Metal-Acceptor	
	D:MG1:MG - PR5:O70	2.39655	Metal-Acceptor	
	PR5:N1 - T:A11	3.99193	Pi-Cation	
	P:U20:OP2 - PR5:Molecule	4.07934	Pi-Anion	
	PR5:C40 - A:CYS622	4.09356	Alkyl	
	PR5:C44 - A:CYS622	4.5621	Alkyl	

*Results show the 5 peptides with highest Total interaction energy target to COVID-19 virus RdRp (7BV2) from Table 2.

Table S11 Interaction mechanism between PA1, PM1, PR1 and three targets after molecular dynamic simulation.

Peptides	*Interaction*	*Distance (Å)*	*Types*	*Interaction Counts*
PA1	A:LYS31:HZ1 - PA1: O148	1.94423	Salt Bridge; Attractive Charge	15
	PA1:H63 - A:GLU37:OE2	2.08591	Salt Bridge; Attractive Charge	
	PA1:N1 - A:GLU35:OE2	2.70357	Attractive Charge	
	PA1:N1 - A:ASP38:OD2	5.17246	Attractive Charge	
	A:LYS31:HZ3 - PA1:O140	1.70062	H-bond	
	PA1:H59 - A:ASP38:OD1	2.18924	H-bond	
	PA1:H65 - A:HIS34:O	1.82896	H-bond	
	PA1:H66 - A:GLU37:OE2	1.86262	H-bond	
	PA1:H103 - A:GLU35:OE1	1.87533	H-bond	
	A:THR27:HB - PA1:O135	2.79888	H-bond	
	A:PHE28:HA - PA1:O135	2.95023	H-bond	
	A:LYS31:HA - PA1:O123	3.0928	H-bond	
	A:LYS31:HE1 - PA1:O148	2.5989	H-bond	
	PA1:H6 - A:GLU35:OE2	2.59029	H-bond	
	PA1:H95 - A:LYS31:O	2.68283	H-bond	
PM1	PM1:N61 - A:GLU166:OE2	5.57125	Attractive Charge	29
	PM1:N99 - A:GLU166:OE2	4.30797	Attractive Charge	
	A:HIS41:HE2 - PM1:O106	2.0784	H-bond	
	A:GLY143:HN - PM1:O82	2.04654	H-bond	
	A:CYS145:HN - PM1:O107	1.92452	H-bond	
	A:GLU166:HN - PM1:O21	2.31394	H-bond	
	PM1:H2 - A:HIS164:O	2.27254	H-bond	
	PM1:H23 - A:GLN189:OE1	1.99195	H-bond	
	PM1:H59 - A:LEU141:O	1.92681	H-bond	
	PM1:H65 - A:PHE140:O	1.79023	H-bond	
	PM1:H97 - A:PHE140:O	2.17889	H-bond	
	PM1:H97 - A:LEU141:O	2.47941	H-bond	
	PM1:H101 - A:GLU166:OE1	1.82131	H-bond	
	PM1:H103 - A:PHE140:O	2.05009	H-bond	
	PM1:H104 - A:GLU166:OE1	2.13798	H-bond	
	A:ASN142:HA - PM1:O82	2.64404	H-bond	
	A:GLY143:HA2 - PM1:O107	2.74514	H-bond	
	A:MET165:HA - PM1:O21	2.39579	H-bond	
	PM1:H25 - A:GLN189:OE1	2.97215	H-bond	
	PM1:H41 - A:GLU166:OE1	2.67991	H-bond	
	PM1:H71 - A:ASN142:OD1	2.81867	H-bond	
	PM1:H94 - A:LEU141:O	2.99877	H-bond	

	PM1:H95 - A:SER144:OG	2.41723	H-bond	
	PM1:N1 - A:HIS41	4.44526	Pi-Cation	
	PM1:C9 - A:MET49	3.54189	Alkyl	
	PM1:C28 - A:PRO168	5.01045	Alkyl	
	PM1:C32 - A:MET165	4.8575	Alkyl	
	A:HIS41 - PM1:C9	4.63614	Pi-Alkyl	
	A:HIS41 - PM1:C16	4.36184	Pi-Alkyl	
PR1	A:LYS551:HZ1 - PR1:O12	1.71641	Salt Bridge; Attractive Charge	30
	PR1:H2 - A:ASP618:OD2	1.81317	Salt Bridge; Attractive Charge	
	D:MG1:MG - PR1:O97	2.21519	Attractive Charge; Metal-Acceptor	
	D:MG2:MG - PR1:O27	2.18792	Attractive Charge	
	D:MG3:MG - PR1:O27	4.50982	Attractive Charge	
	PR1:N1 - A:GLU811:OE2	4.57648	Attractive Charge	
	PR1:N71 - A:ASP623:OD2	2.94757	Attractive Charge	
	A:ARG553:HH11 - PR1:O29	2.48148	H-bond	
	A:ARG553:HH11 - PR1:O38	1.74514	H-bond	
	P:U20:HO3' - PR1:O26	2.51858	H-bond	
	PR1:H3 - A:LYS798:O	2.34771	H-bond	
	PR1:H4 - A:LYS798:O	2.06495	H-bond	
	PR1:H31 - A:ASP760:OD2	2.73991	H-bond	
	PR1:H45 - A:LYS621:O	3.02386	H-bond	
	PR1:H73 - A:TYR456:OH	1.92205	H-bond	
	PR1:H74 - A:THR680:O	2.0661	H-bond	
	PR1:H74 - A:SER681:O	1.63416	H-bond	
	A:LYS551:HE1 - PR1:O12	2.78797	H-bond	
	A:LYS551:HE2 - PR1:O29	2.74768	H-bond	
	A:ARG555:HD2 - PR1:O76	3.01891	H-bond	
	P:U20:H5'2 - PR1:O26	2.21427	H-bond	
	P:U20:H3' - PR1:O26	2.95747	H-bond	
	PR1:H47 - A:LYS621:O	2.85489	H-bond	
	PR1:H47 - A:ASP623:OD1	2.55335	H-bond	
	PR1:H69 - A:THR556:O	2.42739	H-bond	
	D:MG1:MG - PR1:O43	2.05859	Metal-Acceptor	
	D:MG1:MG - PR1:O51	2.10445	Metal-Acceptor	
	D:MG1:MG - PR1:O96	2.26875	Metal-Acceptor	
	D:DENSKF - A:LYS545	5.28614	Pi-Alkyl	
	D:DENSKF - A:ARG555	4.94682	Pi-Alkyl	

Supplemental Figures

Supplemental Figure S1 Structure of the ACE2 and reported inhibitory drugs.

A, Structure downloaded from PDB website (PDB: 6M0J).

B, Blank structure of ACE2 by taking out the COVID-19 spike protein receptor binding domain from 6M0J.

C, Docking site of ACE2, marked in red sphere object with radiu of 22.718 Å.

D-F, Structure of reported molecules with inhibitory activity against the ACE2. D represents the structure of MLN-4760, CAS NO. 305335-31-3; E represents DX600, CAS NO. N/A. The structure is downloaded from DrugBank.

Supplemental Figure S2. Structure of ACE2 complex containing Inhibitor MLN-4760 and its binding modes.

A, 3D Structure of ACE2 complex containing inhibitor MLN-4760. For clarity, the interface receptor amino acids residues are shown in thin stick style and inhibitor MLN-4760 is shown in thick stick style. ACE2 is downloaded from PDB (No. 6M0J) and generated in DS software with deleting the ligands. Different color represents different secondary structure, red is α-helix, blue is β-fold, green is β-turn, white is random coil.

B, Enlarged view of the interface in ACE2 complex containing inhibitor MLN-4760.

C-D, Binding site between Inhibitor MLN-4760 and ACE2 and the main interaction residues. Dashed line with different color represents the Non-covalent interaction between Inhibitor MLN-4760 and ACE2.

E, 2D representation of interactions between the Inhibitor MLN-4760 and amino acid residues of ACE2.

Supplemental Figure S3. Structure feature of the docking pocket based on the overlap inhibitors.

A, H-Bonds donor and acceptor property of the docking site of ACE2. The overlapped inhibitors are shown in line style and the surface color represents the H-bonds donor and acceptor property, red represents donor and green represents acceptor.

B, Hydrophobicity property of the docking site of ACE2. The overlapped inhibitors are

shown in line style and the surface color represents the hydrophobicity property, brown represents more hydrophobicity and blue represents more hydrophilicity.

C, Ionizability property of the docking site of ACE2. The overlapped inhibitors are shown in line style and the surface color represents the Ionizability property, blue represents more basic; red represents more acidic.

D, Aromatic property of the docking site of ACE2. The overlapped inhibitors are shown in line style and the surface color represents the aromatic property, blue represents more edge of aromatic group and brown represents more face of aromatic group.

E-F, Overlap of all inhibitory molecules and their representative feature in different area. Red dashed ring in E represents the typical H-bond acceptor of inhibitor and blue dashed ring represents the H-bond donor. Red dashed ring in F represents the typical acidic site of inhibitor and blue dashed ring represents the typical basic site.

G-H, Structure feature of complex of the overlapped inhibitors and the main bind amnio acid residues.

Supplemental Figure S4 Structure of the ACE2 complex containing PA1 and its binding modes after molecule dynamic simulation.

A, 3D Structure of the ACE2 complex containing PA1. For clarity, the interface receptor amino acids residues are shown in thin stick style and PA1 is shown in thick stick style. ACE2 is downloaded from PDB (No. 6M0J) and generated in DS software with deleting the ligands. Different color represents different secondary structure, red is α-helix, blue is β-fold, green is β-turn, white is random coil. PA1 is the peptide, Ile-Val-Gly-Arg-Pro-Arg-His-Gln-Gly.

B, Enlarged view of the interface in ACE2 complex containing PA1.

C-D, Binding site between PA1 and ACE2 and the main interaction residues. Dashed line with different color represents the Non-covalent interaction between PA1 and ACE2.

E, 2D representation of interactions between PA1 and amino acid residues of ACE2.

Supplemental Figure S5 Docking Poses of the top three peptides inhibiting ACE2

comparing with MLN-4760.

The inhibitors are shown in stick style and the background color represents the hydrophobicity, brown show more hydrophobicity and blue show less.

Supplemental Figure S6 Structure of the COVID-19 virus M^{pro} and reported inhibitory drugs.

A, Structure downloaded from PDB website (PDB: 6LU7).

B, Blank structure of the COVID-19 virus M^{pro} by taking out the inhibitor N3 from 6LU7.

C, Docking site from the receptor cavities with manually modification, S1-S5.

D-F, Structure of reported molecular (drugs) with inhibitory activity against the COVID-19 virus M^{pro}. D represents inhibitory N3 from 6LU7; E represents Ritonavir, CAS NO. 155213-67-5; F represents Lopinavir, CAS, NO. 192725-17-0. The structure is downloaded from DrugBank.

Supplemental Figure S7 Structure of the COVID-19 virus M^{pro} complex containing Inhibitor N3 and its binding modes.

A, 3D Structure of the COVID-19 virus M^{pro} complex containing Inhibitor N3. For clarity, the interface receptor amino acids residues are shown in thin stick style and Inhibitor N3 is shown in thick stick style. Whole stucture is downloaded from PDB webstie (No. 6LU7)

B, Enlarged view of the interface in COVID-19 virus M^{pro} complex containing inhibitor N3.

C-D, Binding site between Inhibitor N3 and COVID-19 virus M^{pro} and the main interaction residues. Dashed line with different color represents the Non-covalent interaction between Inhibitor N3 and COVID-19 virus M^{pro}. Red dashed ring shows the S-C covalent bond between cysteine and Inhibitor N3.

E, 2D representation of interactions between the Inhibitor N3 and amino acid residues of COVID-19 virus M^{pro}.

Supplemental Figure S8. Structure feature of the docking pocket based on the overlap inhibitors.

A, H-Bonds donor and acceptor property of the docking pocket of COVID-19 virus M^{pro}. The overlapped inhibitors are shown in line style and the surface color represents the H-bonds donor and acceptor property, red represents donor and green represents acceptor.

B, Hydrophobicity property of the docking pocket of COVID-19 virus M^{pro}. The overlapped inhibitors are shown in line style and the surface color represents the hydrophobicity property, brown represents more hydrophobicity and blue represents more hydrophilicity.

C, Ionizability property of the docking pocket of COVID-19 virus M^{pro}. The overlapped inhibitors are shown in line style and the surface color represents the Ionizability property, blue represents more basic; red represents more acidic.

D, Aromatic property of the docking pocket of COVID-19 virus M^{pro}. The overlapped inhibitors are shown in line style and the surface color represents the aromatic property, blue represents more edge of aromatic group and brown represents more face of aromatic group.

E-F, Overlap of all inhibitory molecules and their representative feature in different area. Red dashed ring in E represents the typical H-bond acceptor of inhibitor and blue dashed ring represents the H-bond donor. Red dashed ring in F represents the typical acidic site of inhibitor and blue dashed ring represents the typical basic site.

G-H, Structure feature of complex of the overlapped inhibitors and the main bind amnio acid residues.

Supplemental Figure S9 Structure of the COVID-19 virus M^{pro} complex containing PM1 and its binding modes after molecule dynamic simulation.

A, 3D Structure of the COVID-19 virus M^{pro} complex containing PM1. For clarity, the interface receptor amino acids residues are shown in thin stick style and PM1 is shown in thick stick style. PM1 is the peptide, Ile-Val-Gly-Arg-Pro-Arg.

B, Enlarged view of the interface in COVID-19 virus M^{pro} complex containing PM1.
C-D, Binding site between PM1 and COVID-19 virus M^{pro} and the main interaction residues. Dashed line with different color represents the Non-covalent interaction between PM1 and COVID-19 virus M^{pro}.
E, 2D representation of interactions between PM1 and amino acid residues of COVID-19 virus M^{pro}.

Supplemental Figure S10 Docking Poses of the top three peptides inhibiting COVID-19 virus Mpro comparing with Inhibitor N3.
The inhibitors are shown in stick style and the background color represents the hydrophobicity, brown show more hydrophobicity and blue show less.

Supplemental Figure S11 Structure of the COVID-19 virus RdRp and reported inhibitory drugs.
A, Structure downloaded from PDB website (PDB: 7BV2).
B, Blank structure of the COVID-19 virus RdRp by taking out the Ridsivir monophosphate (RMP) and from 7BV2.
C, Docking site of COVID-19 virus RdRp, marked in red sphere object with radiu of 8.705 Å
D-F, Structure of reported molecular (drugs) with inhibitory activity against the COVID-19 virus RdRp. D represents Remdesivir, CAS NO. 1809249-37-3; E represents Ridsivir monophosphate (RMP); F represents Polyphosphate (POP). The structure is downloaded from DrugBank.

Supplemental Figure S12 Structure of the COVID-19 virus RdRp complex containing Remdesivir and its binding modes.
A, 3D Structure of the COVID-19 virus RdRp complex containing Remdesivir. For clarity, the interface receptor amino acids residues are shown in thin stick style and Remdesivir (RMP, POP) is shown in thick stick style. Whole stucture is downloaded from PDB webstie (No. 7BV2).

B, Enlarged view of the interface in COVID-19 virus RdRp complex containing Remdesivir.

C-D, Binding site between RMP and COVID-19 virus RdRp and the main interaction residues. Dashed line with different color represents the Non-covalent interaction between RMP and COVID-19 virus RdRp. Red dashed ring shows the O-P covalent bond between Uracil ribonucleotide and RMP.

E, 2D representation of interactions between RMP and amino acid residues of COVID-19 virus RdRp.

Supplemental Figure S13 Structure feature of the docking pocket based on the overlap inhibitors.

A, H-Bonds donor and acceptor property of the docking pocket of COVID-19 virus RdRp. The overlapped inhibitors are shown in line style and the surface color represents the H-bonds donor and acceptor property, red represents donor and green represents acceptor.

B, Hydrophobicity property of the docking pocket of COVID-19 virus RdRp. The overlapped inhibitors are shown in line style and the surface color represents the hydrophobicity property, brown represents more hydrophobicity and blue represents more hydrophilicity.

C, Ionizability property of the docking pocket of COVID-19 virus RdRp. The overlapped inhibitors are shown in line style and the surface color represents the Ionizability property, blue represents more basic; red represents more acidic.

D, Aromatic property of the docking pocket of COVID-19 virus RdRp. The overlapped inhibitors are shown in line style and the surface color represents the aromatic property, blue represents more edge of aromatic group and brown represents more face of aromatic group.

E-F, Overlap of all inhibitory molecules and their representative feature in different area. Red dashed ring in E represents the typical H-bond acceptor of inhibitor and blue dashed ring represents the H-bond donor. Red dashed ring in F represents the typical acidic site of inhibitor and blue dashed ring represents the typical basic site.

G-H, Structure feature of complex of the overlapped inhibitors and the main bind amnio acid residues.

Supplemental Figure S14 Structure of the COVID-19 virus RdRp complex containing PR1 and its binding modes after molecular dynamic simulation.

A, 3D Structure of the COVID-19 virus RdRp complex containing PR1. For clarity, the interface receptor amino acids residues are shown in thin stick style and PR1 is shown in thick stick style. PR1 is the peptides, Asp-Glu-Asn-Ser-Lys-Phe.

B, Enlarged view of the interface in COVID-19 virus RdRp complex containing PR1.

C-D, Binding site between PR1 and COVID-19 virus RdRp and the main interaction residues. Dashed line with different color represents the Non-covalent interaction between PR1 and COVID-19 virus RdRp.

E, 2D representation of interactions between PR1 and amino acid residues of COVID-19 virus RdRp.

Supplemental Figure S15 Docking Poses of the top three peptides inhibiting COVID-19 virus RdRp comparing with Remdesivir.

The inhibitors are shown in stick style and the background color represents the hydrophobicity, brown show more hydrophobicity and blue show less.

Supplemental Figure S1

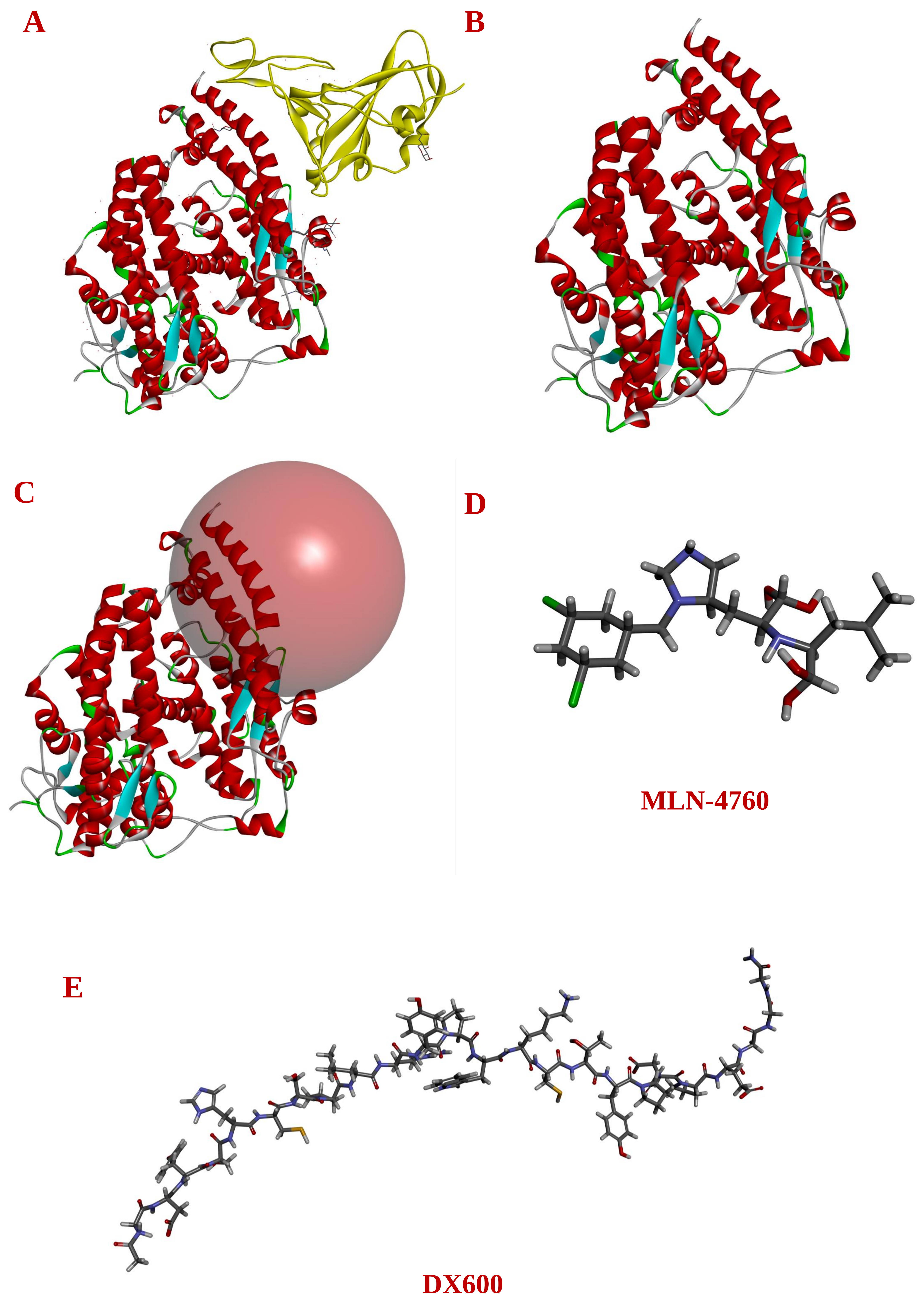

Supplemental Figure S2

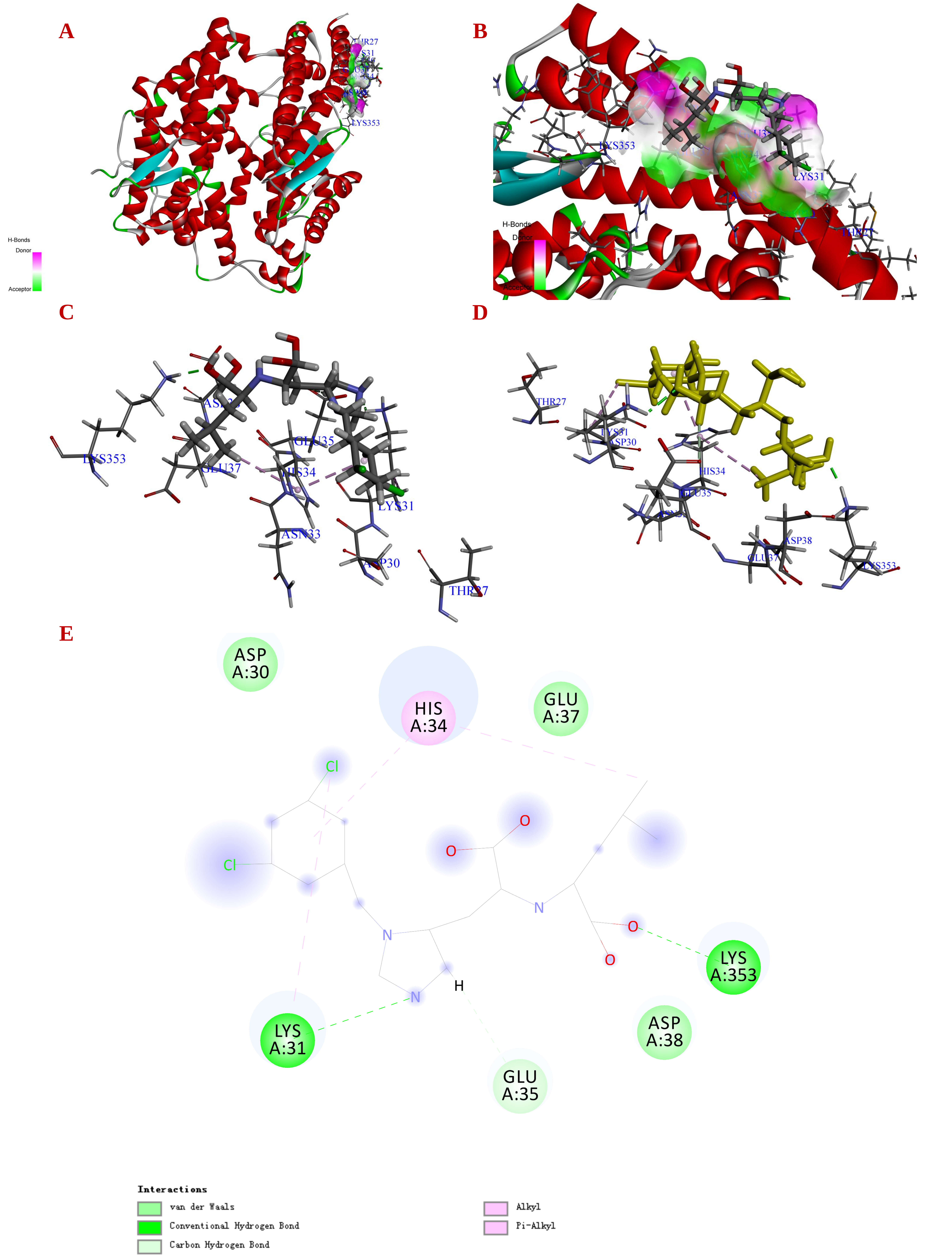

Supplemental Figure S3

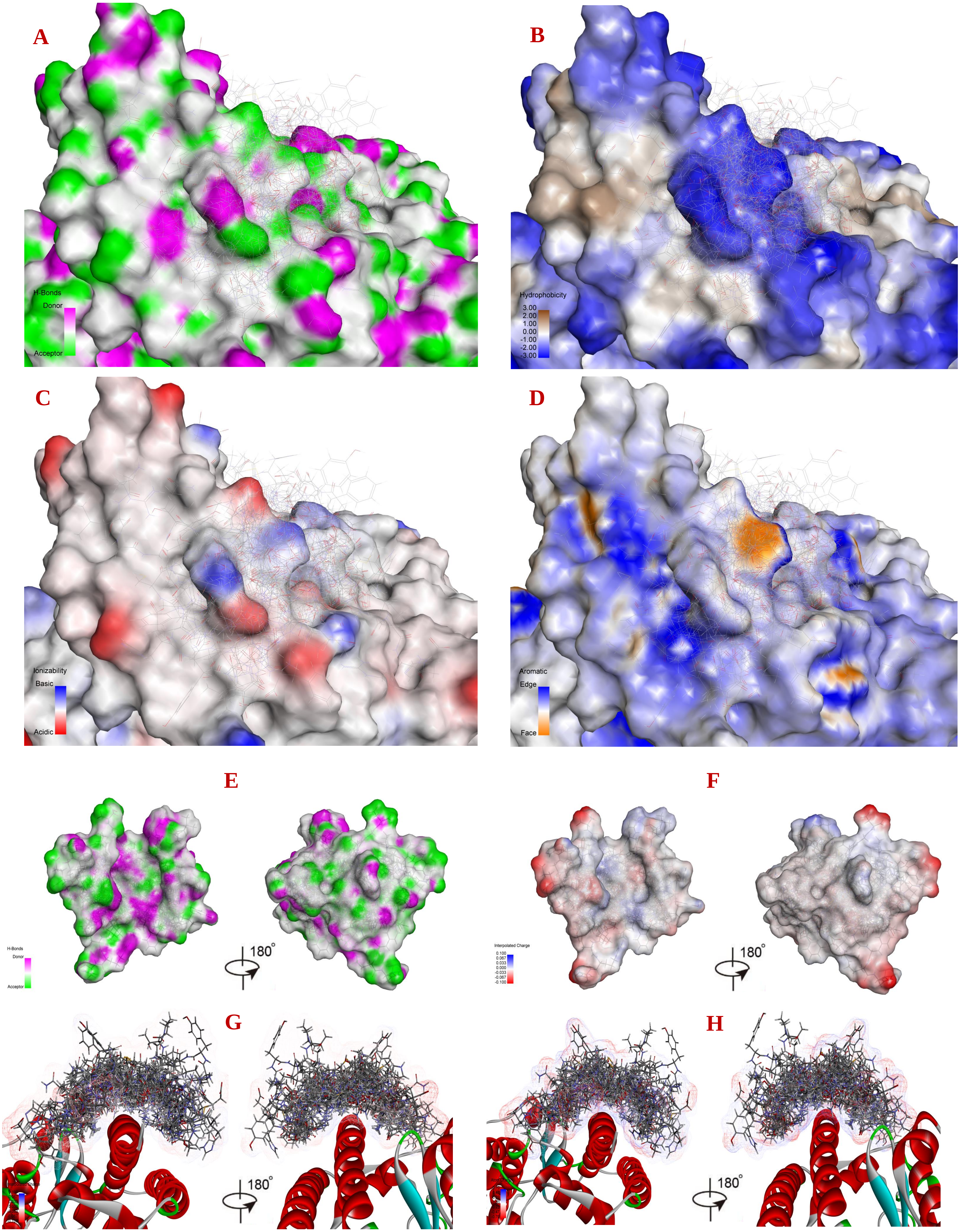

Supplemental Figure S4

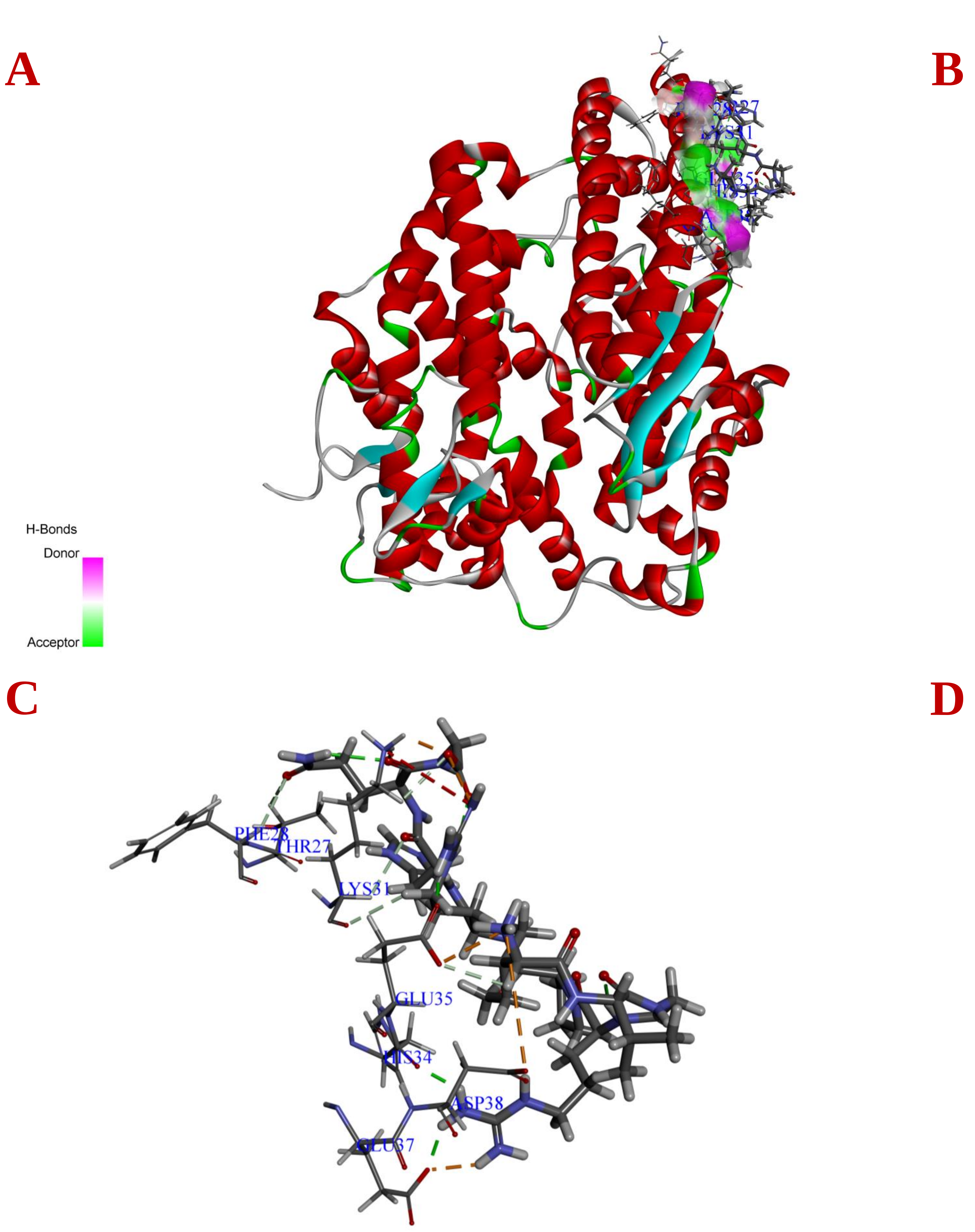

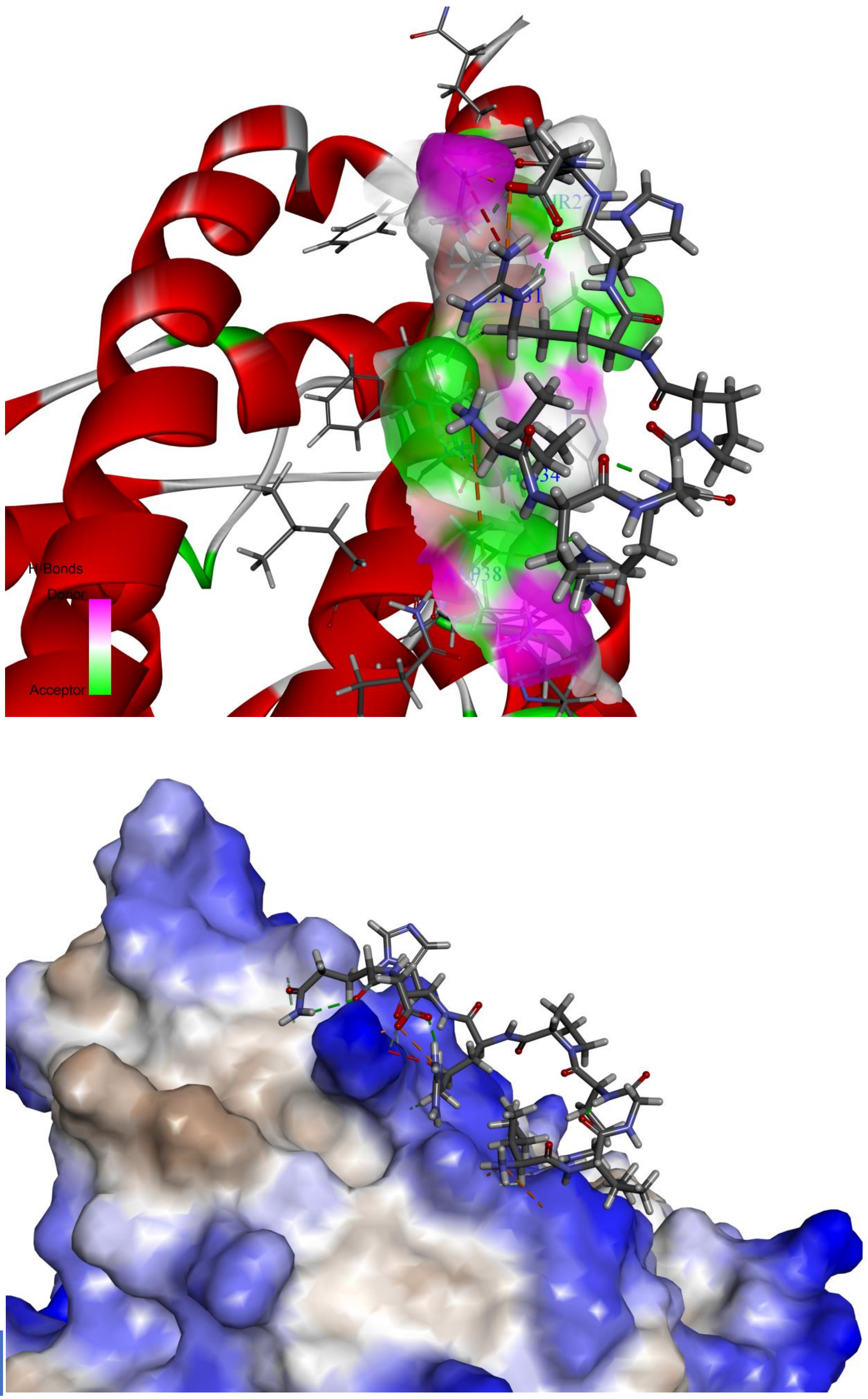

Molecule	Total Interaction Energy(kcal/mol)
PA1	-440.463287

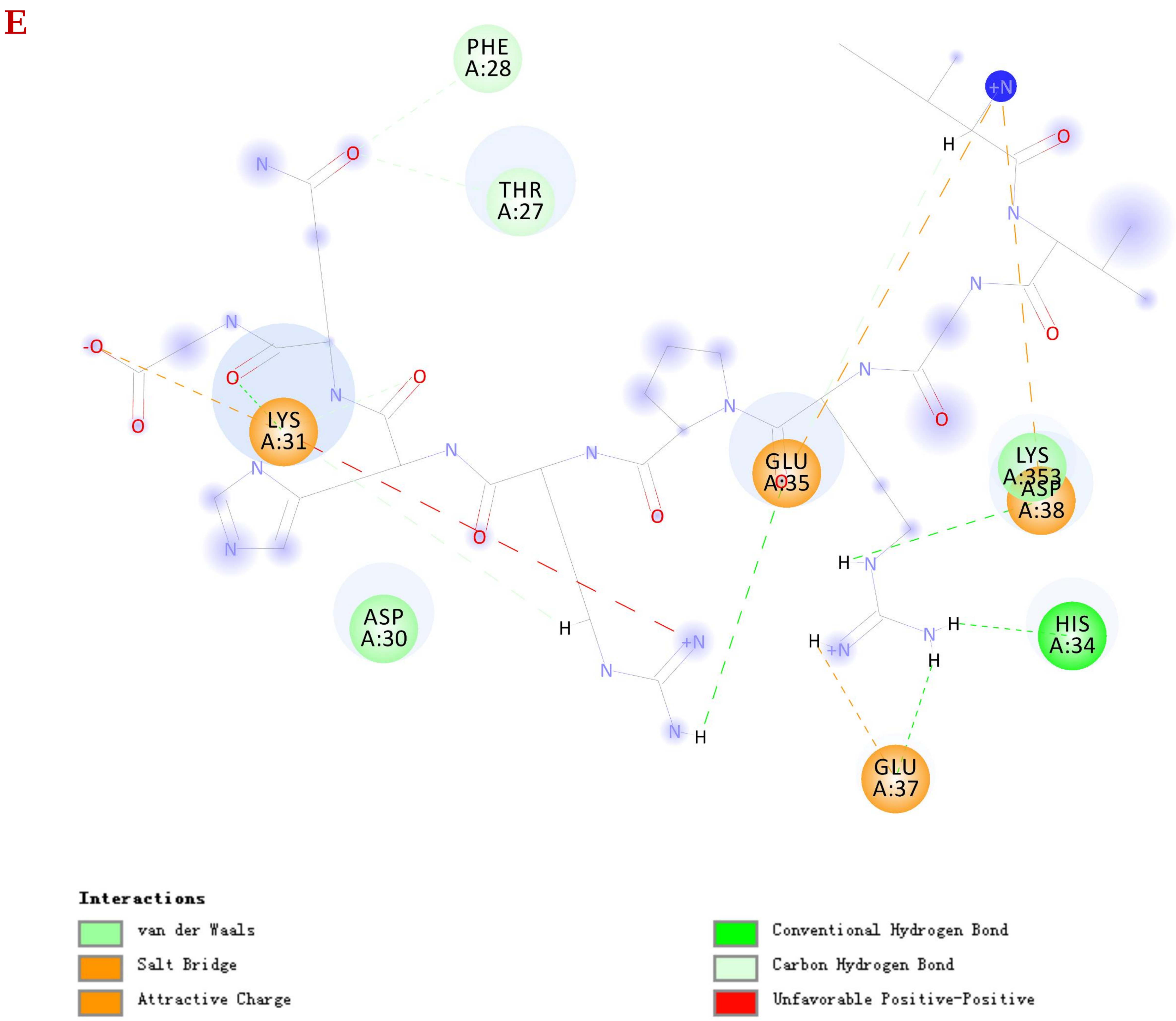

Supplemental Figure S5

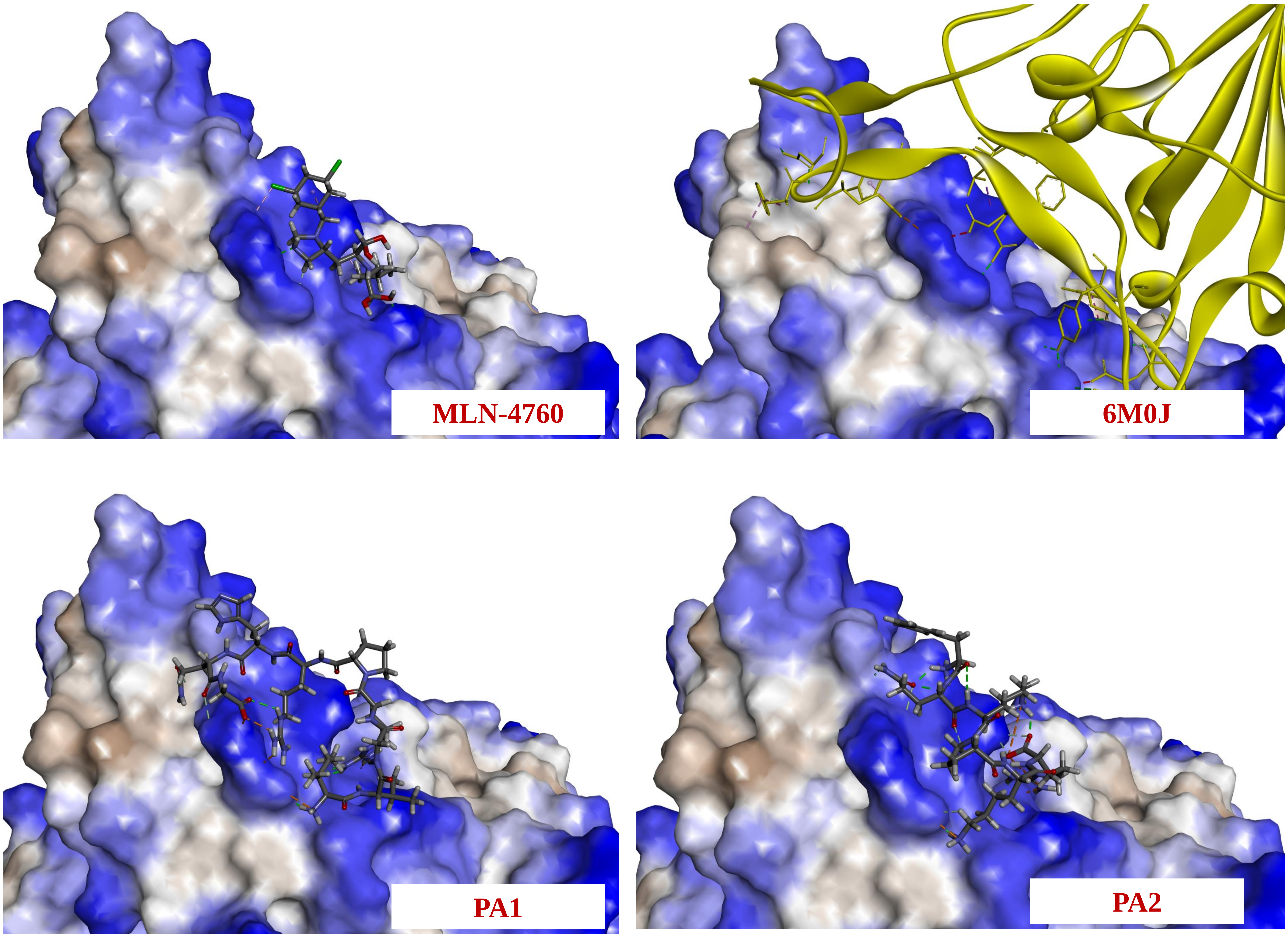

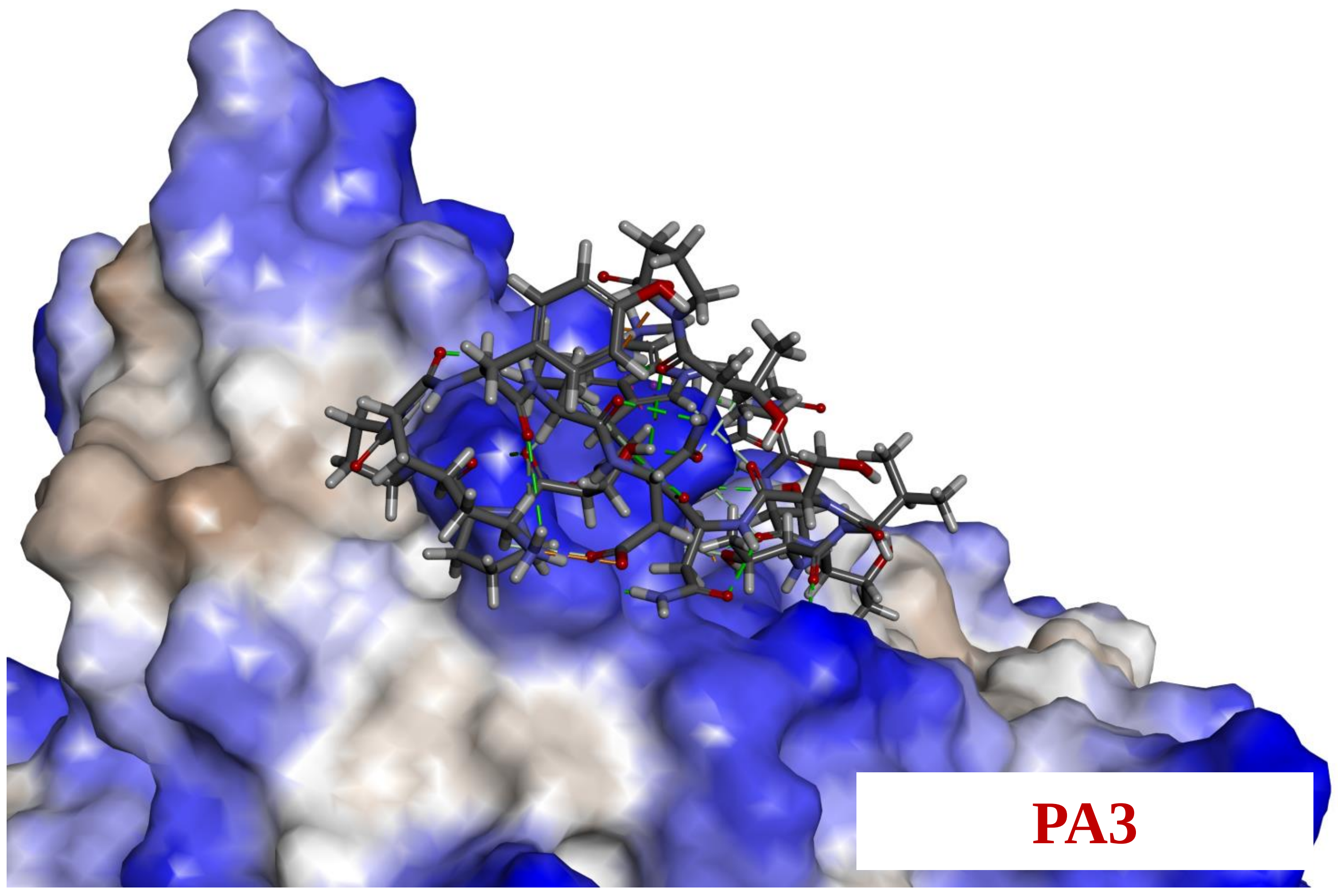

Molecule	Total Interaction Energy (kcal/mol)
MLN-4760	-86.40653
Spike Protein	-158.34175
PA1	-330.55571
PA2	-283.75101
PA3	-272.21947

Supplemental Figure S6

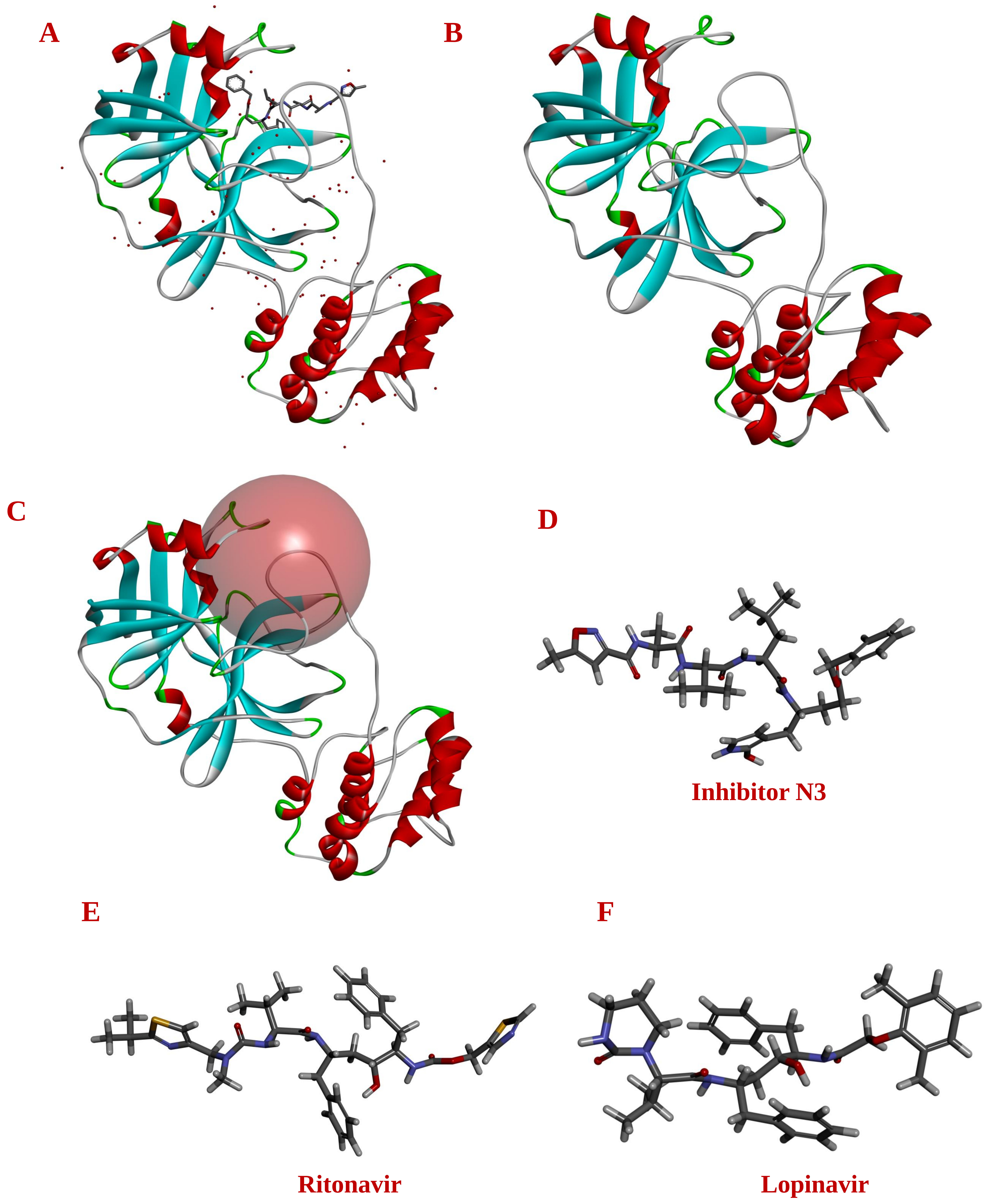

Supplemental Figure S7

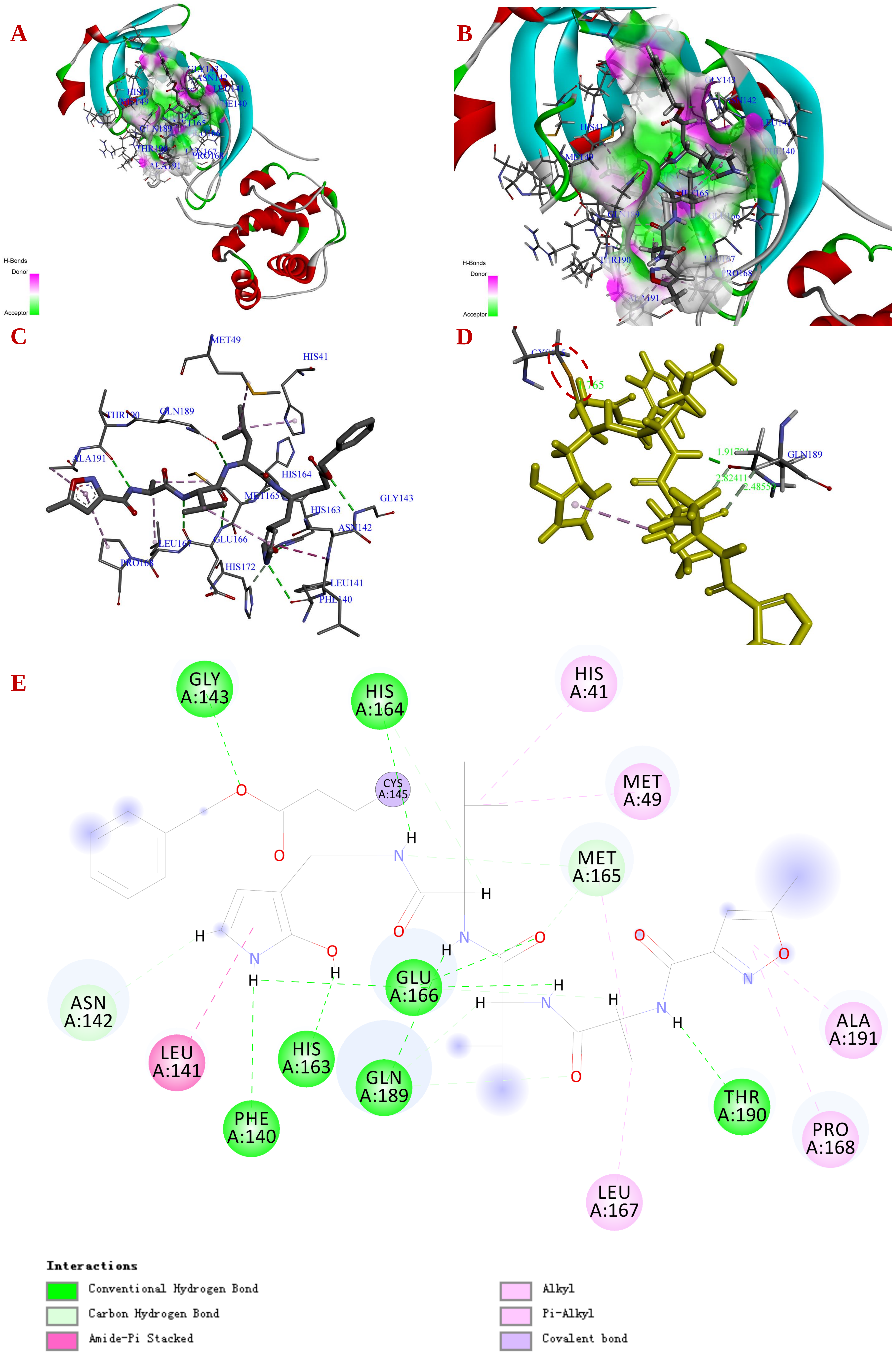

Supplemental Figure S8

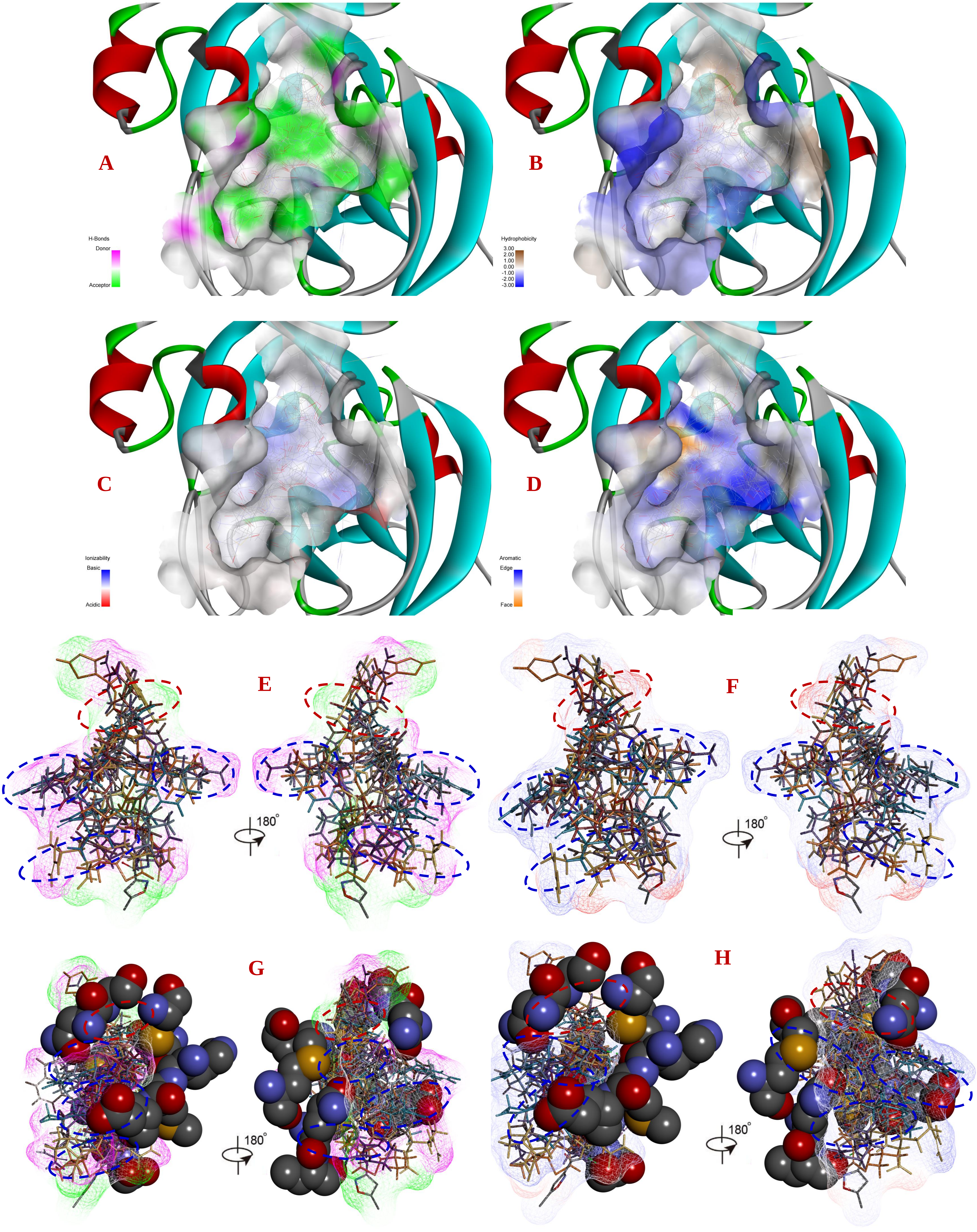

Supplemental Figure S9

A

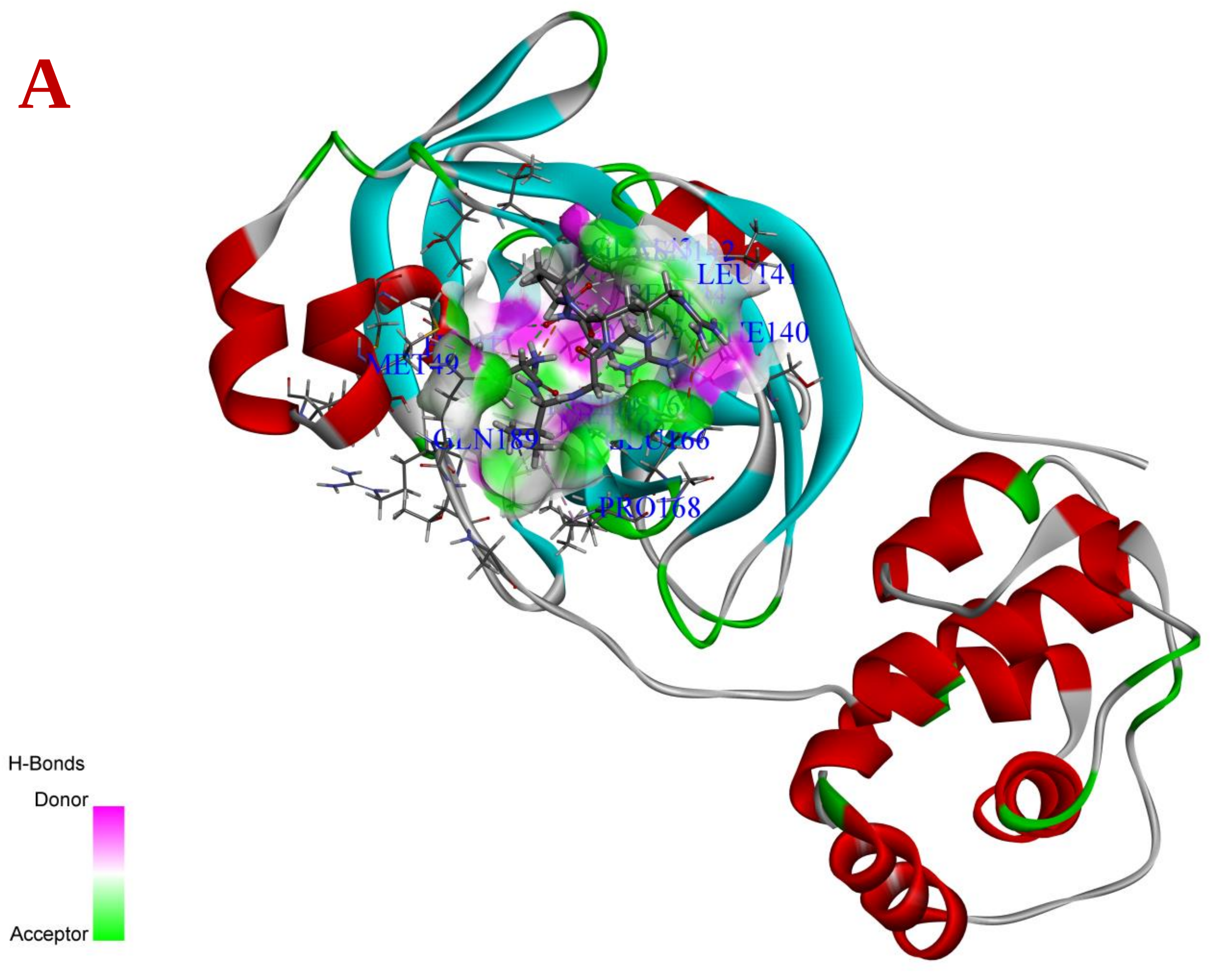

B

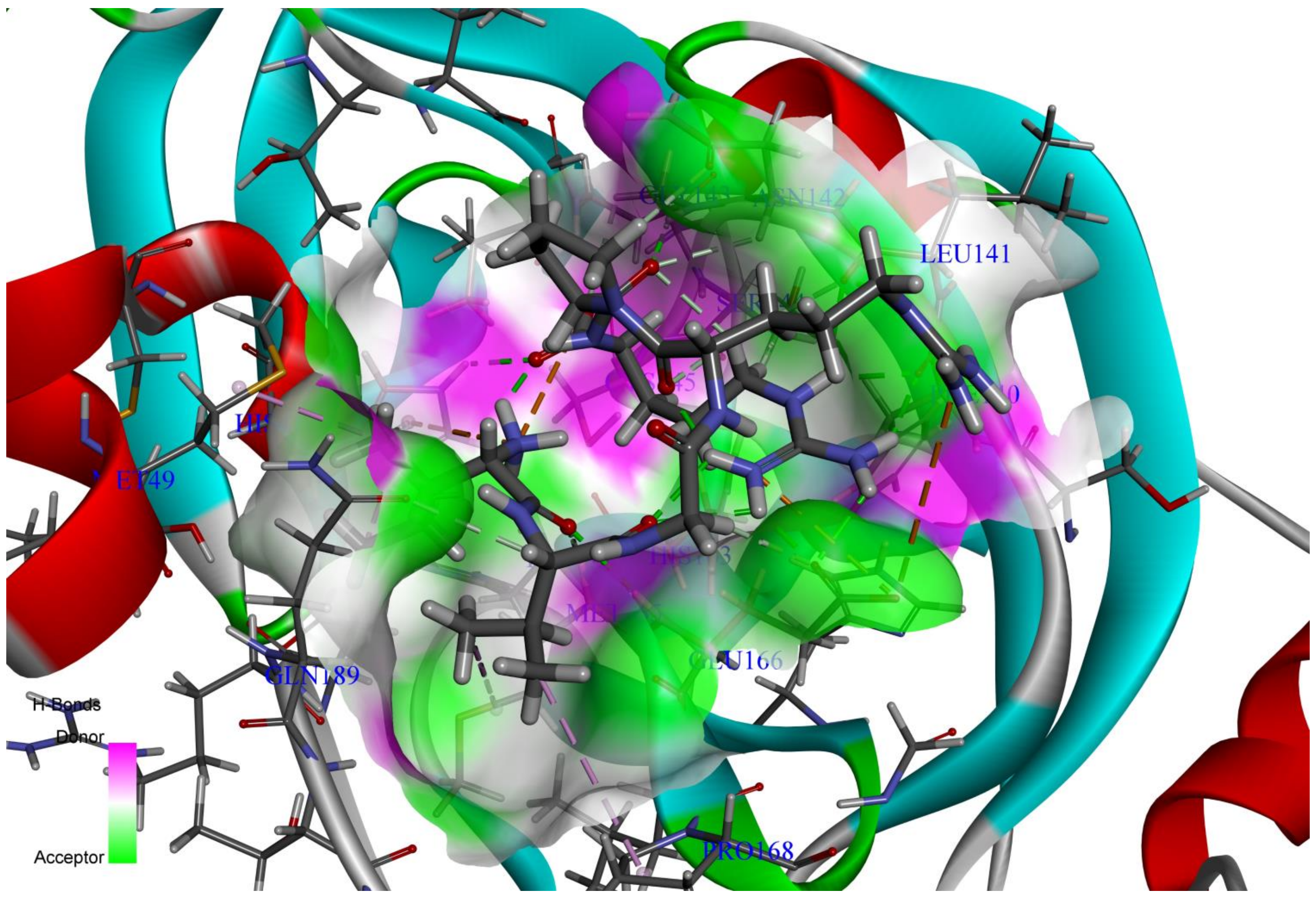

C

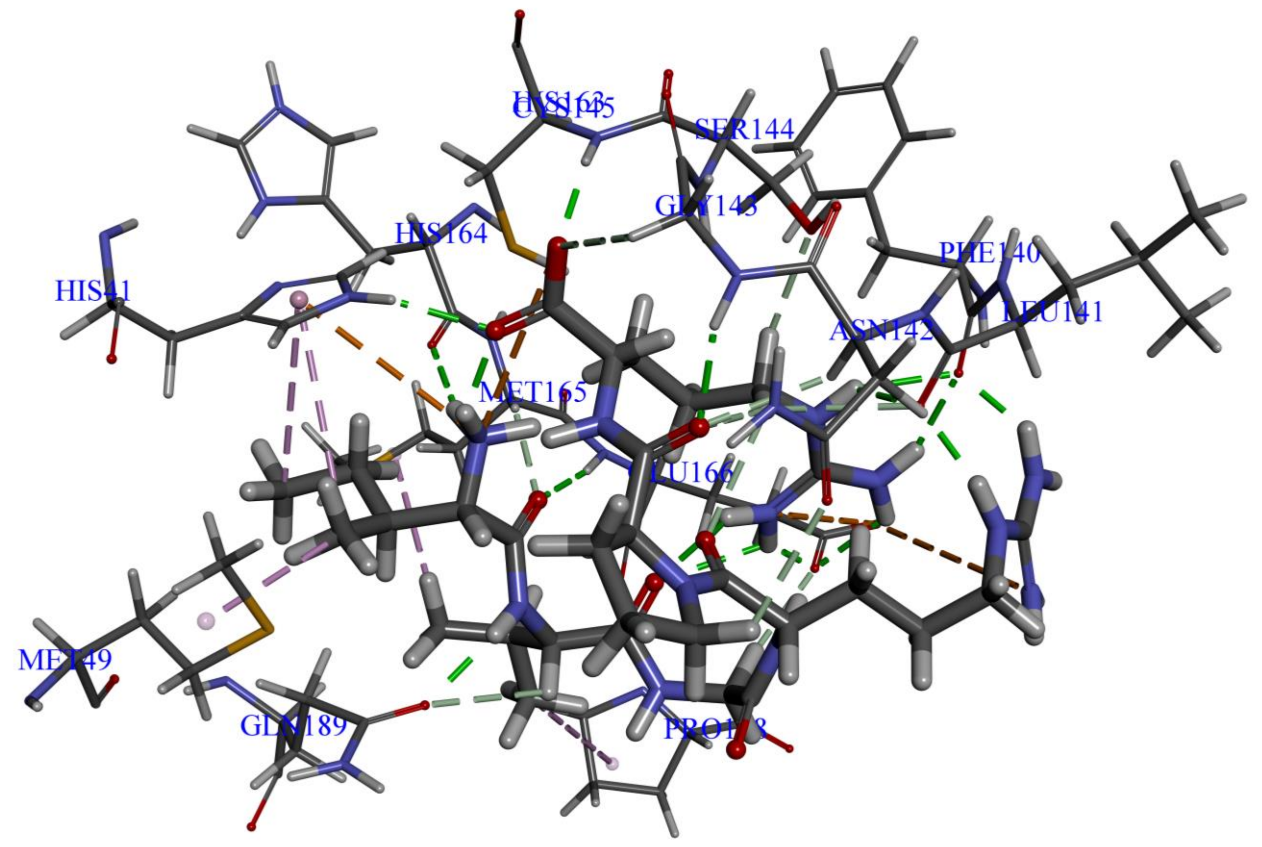

Molecule	Total Interaction Energy(kcal/mol)
PM1	-341.462362

D

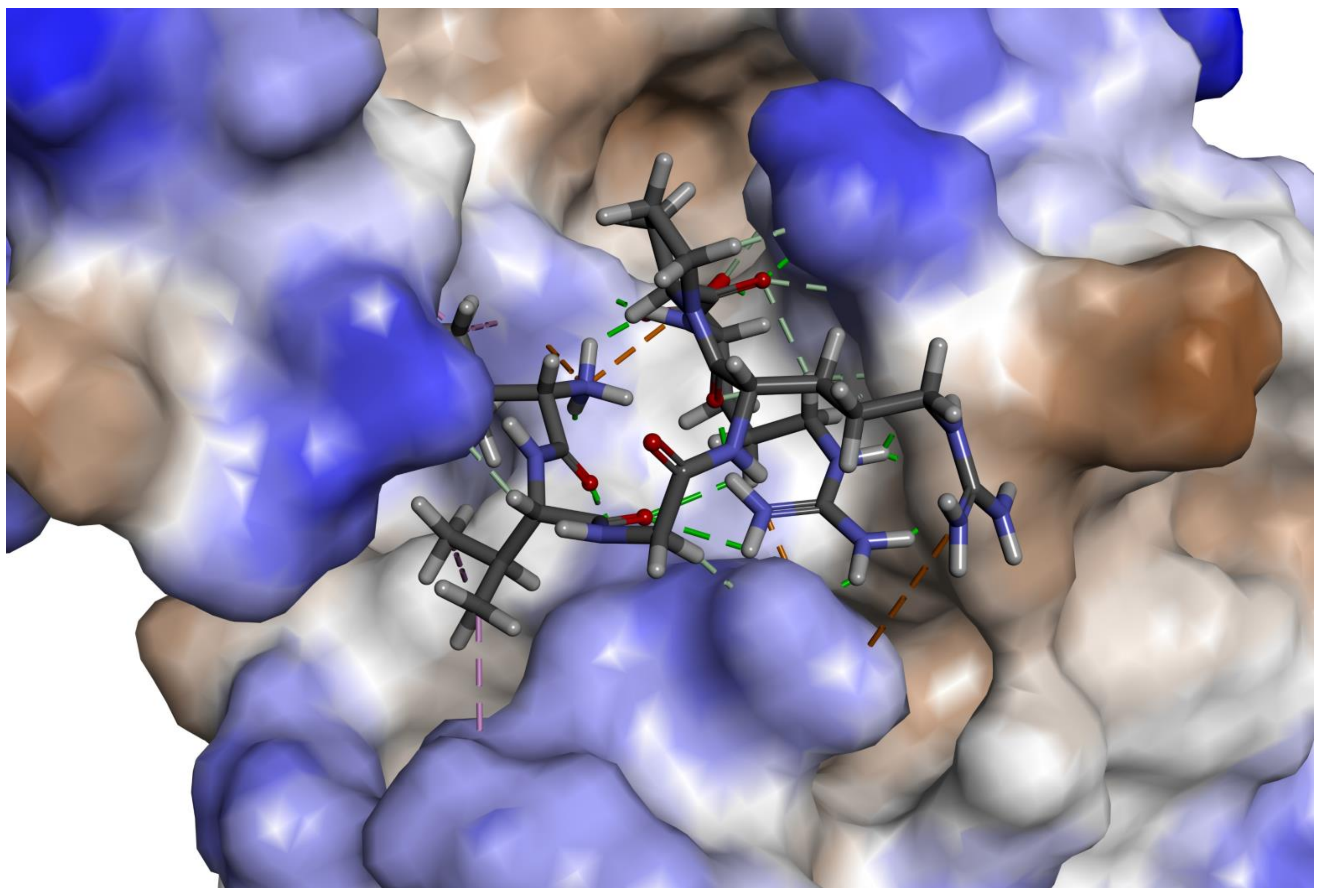

E

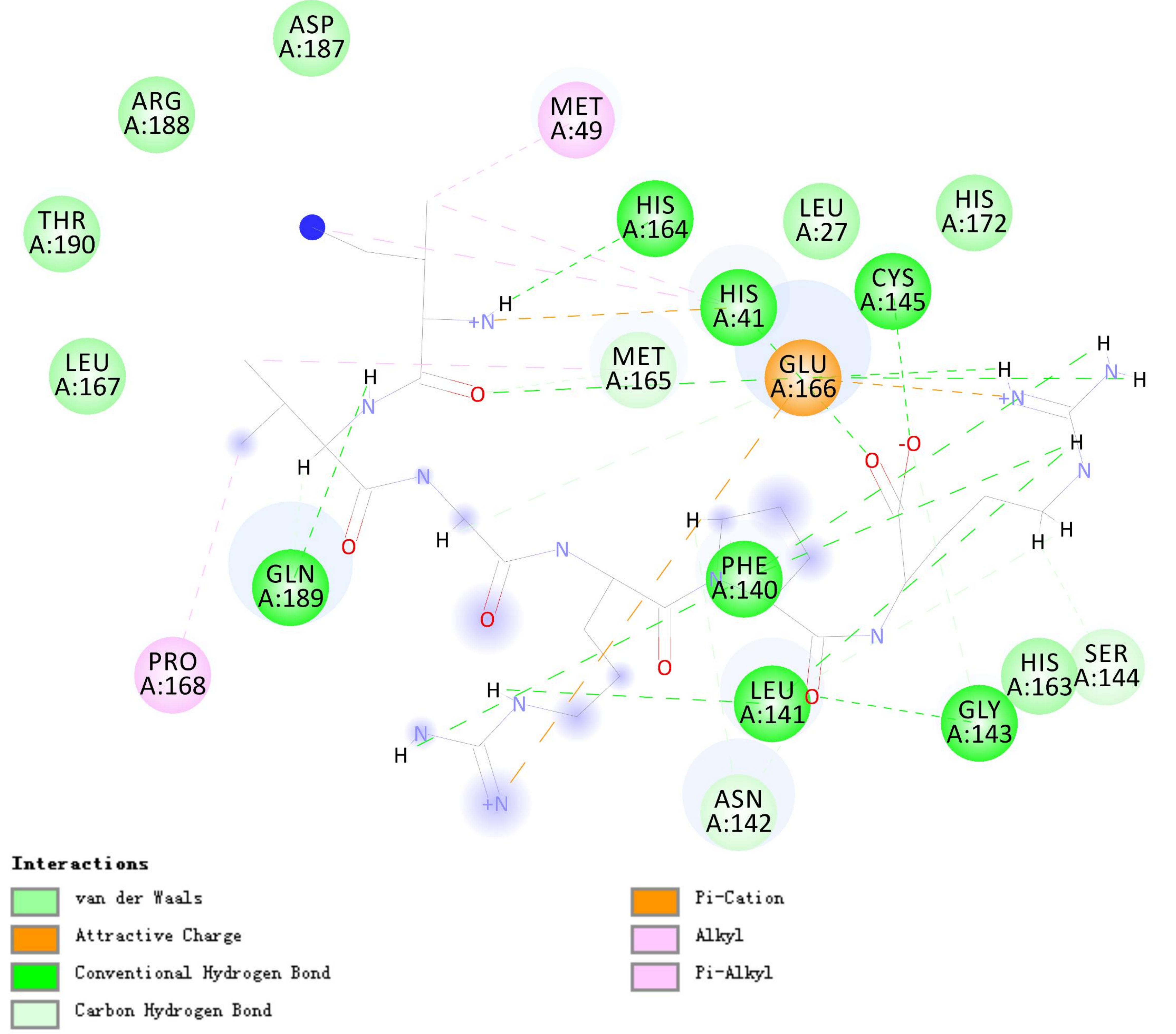

Supplemental Figure S10

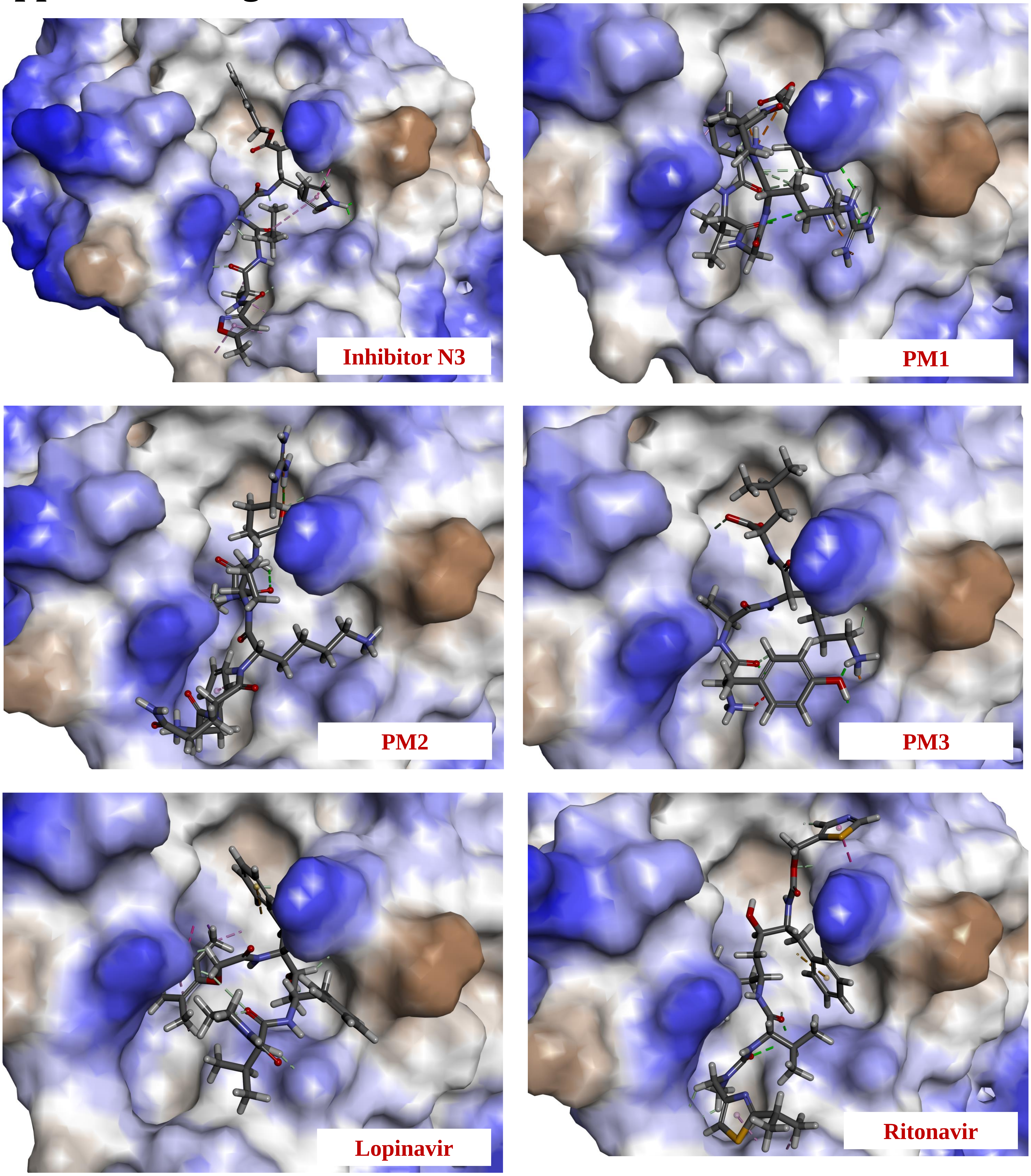

Molecule	Total Interaction Energy (Non-covalent, kcal/mol)
Inhibitor N3	-36.018383
Lopinavir	-102.49987
Ritonavir	-87.46983
PM1	-366.21436
PM2	-308.37838
PM3	-306.44818

Supplemental Figure S11

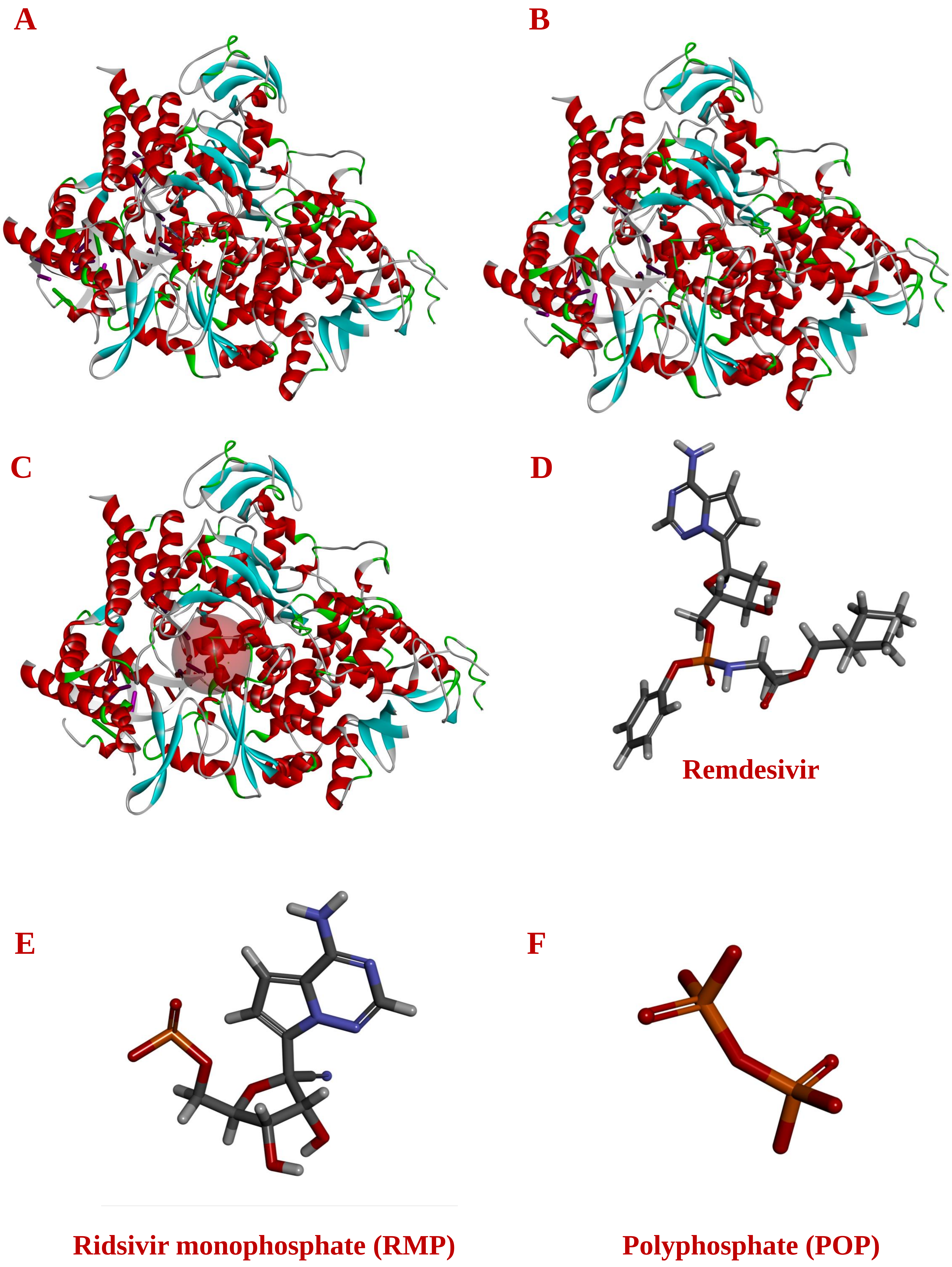

Supplemental Figure S12

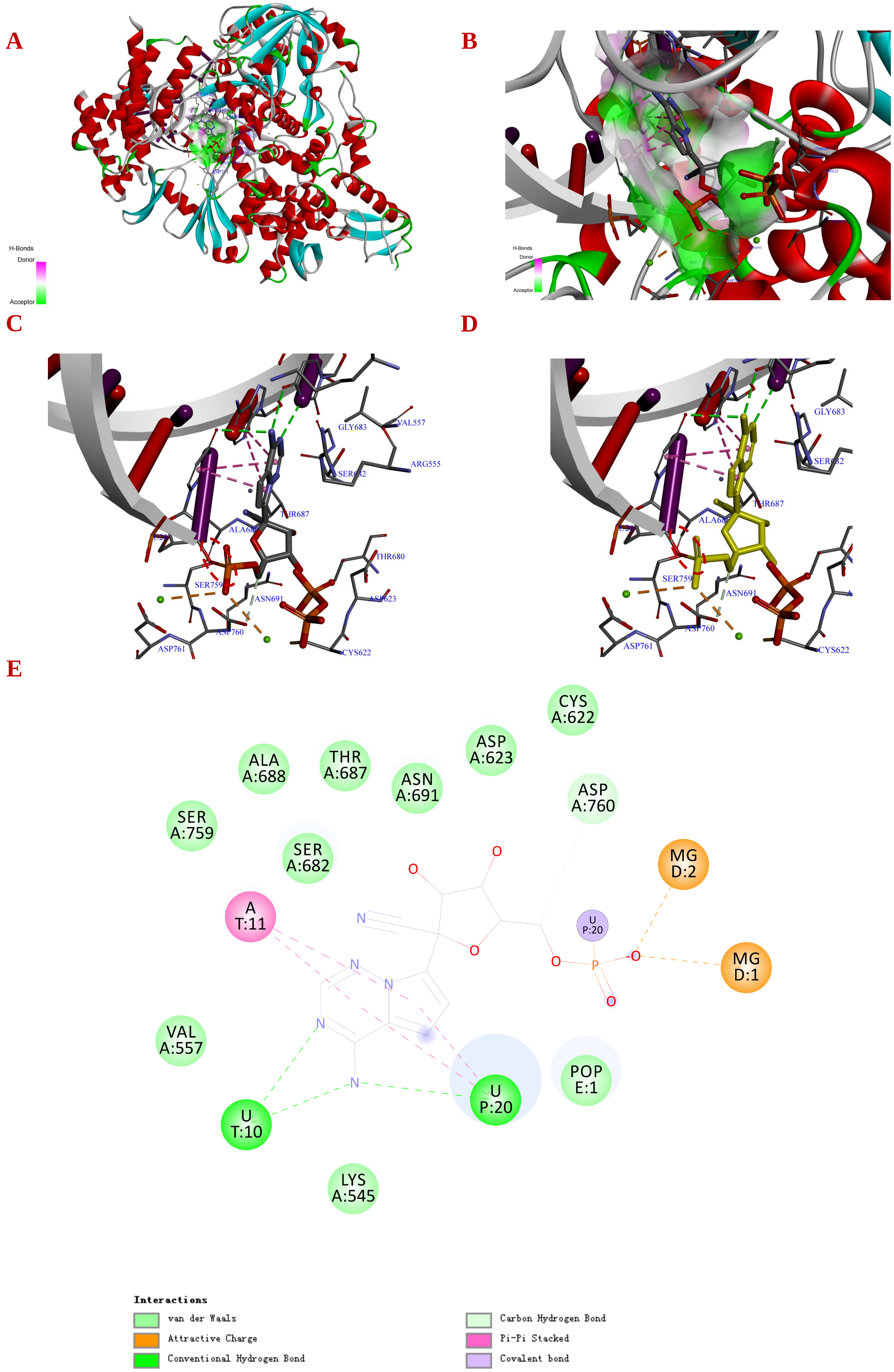

Supplemental Figure S13

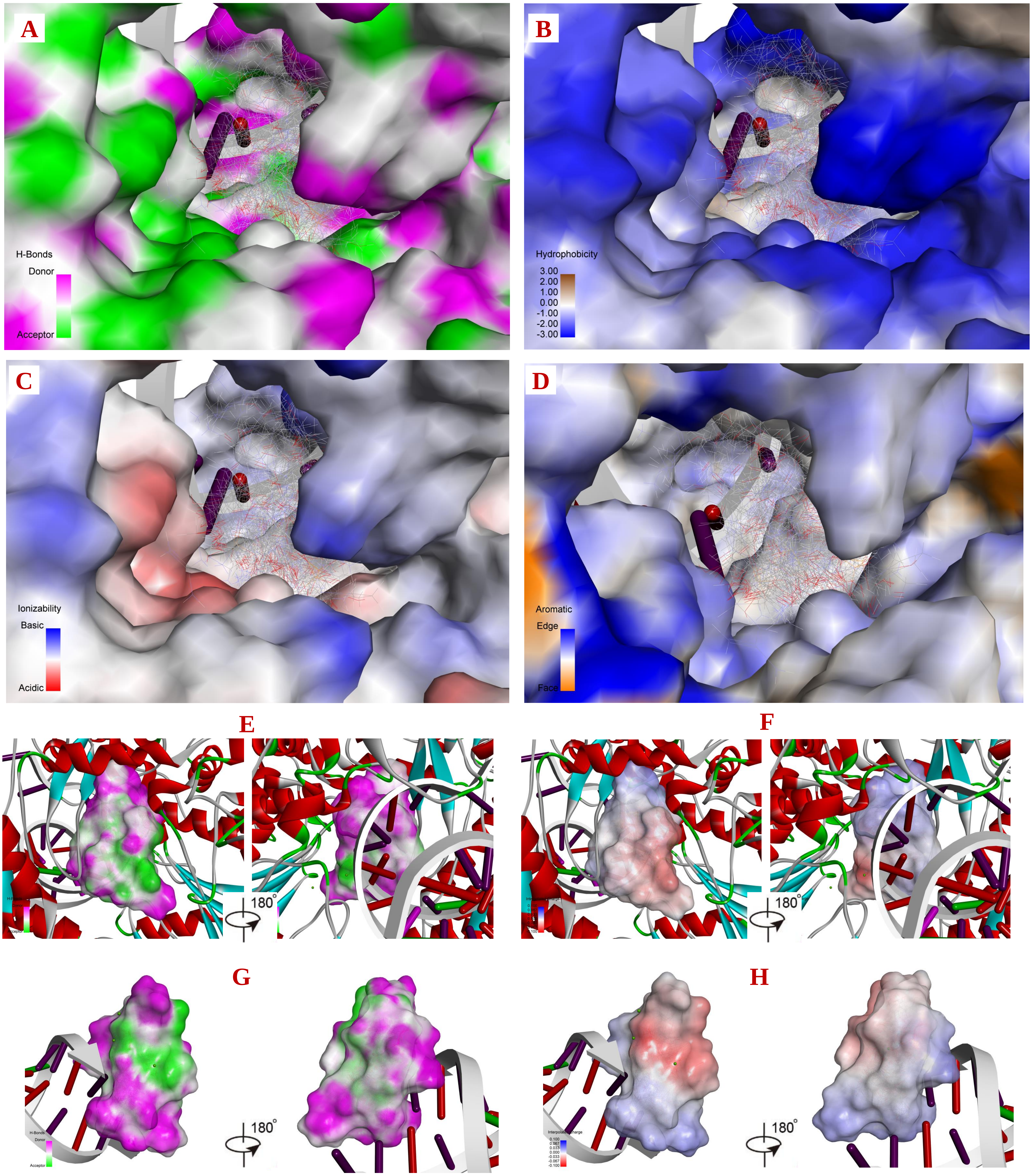

Supplemental Figure S14

A

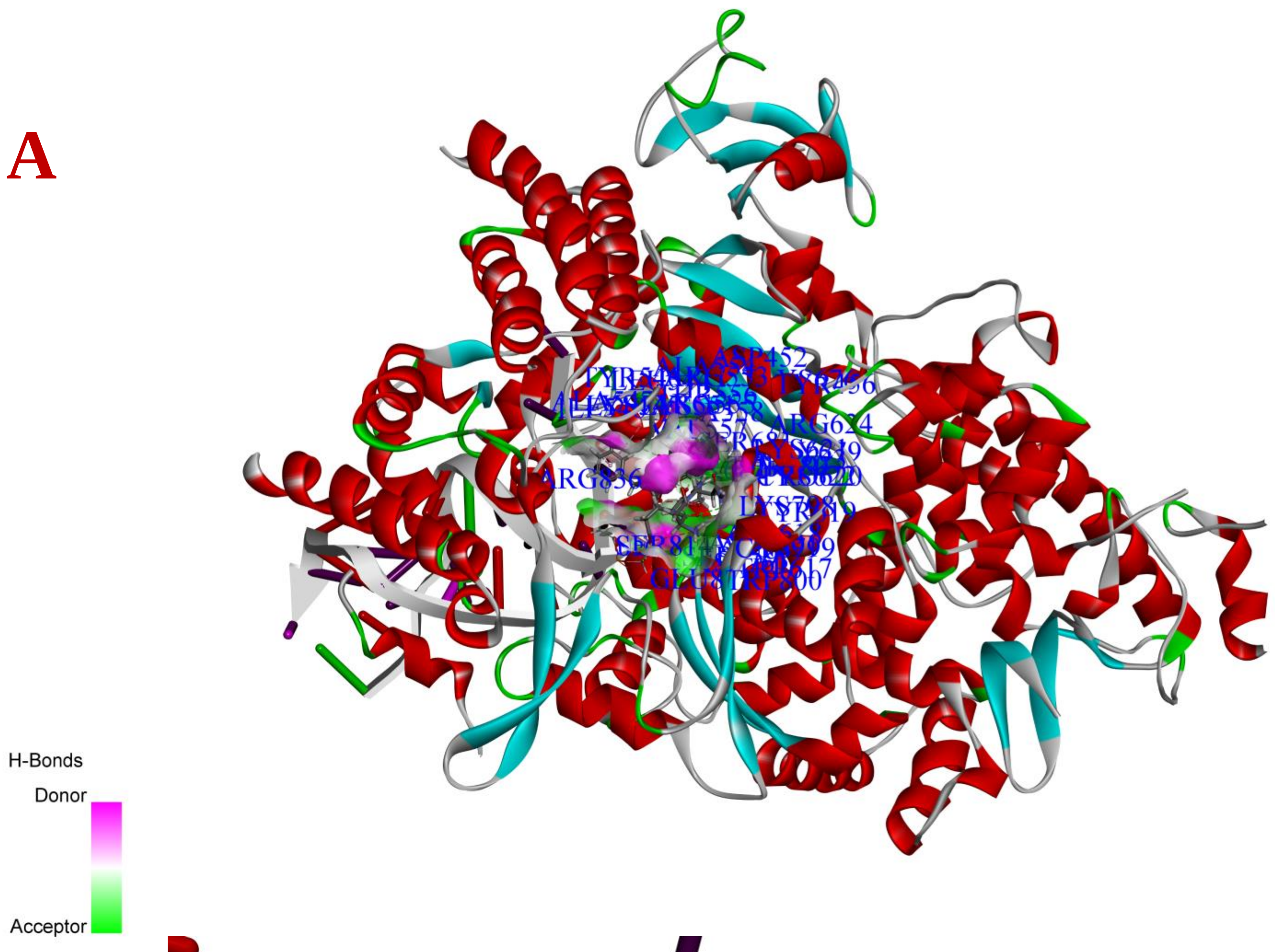

B

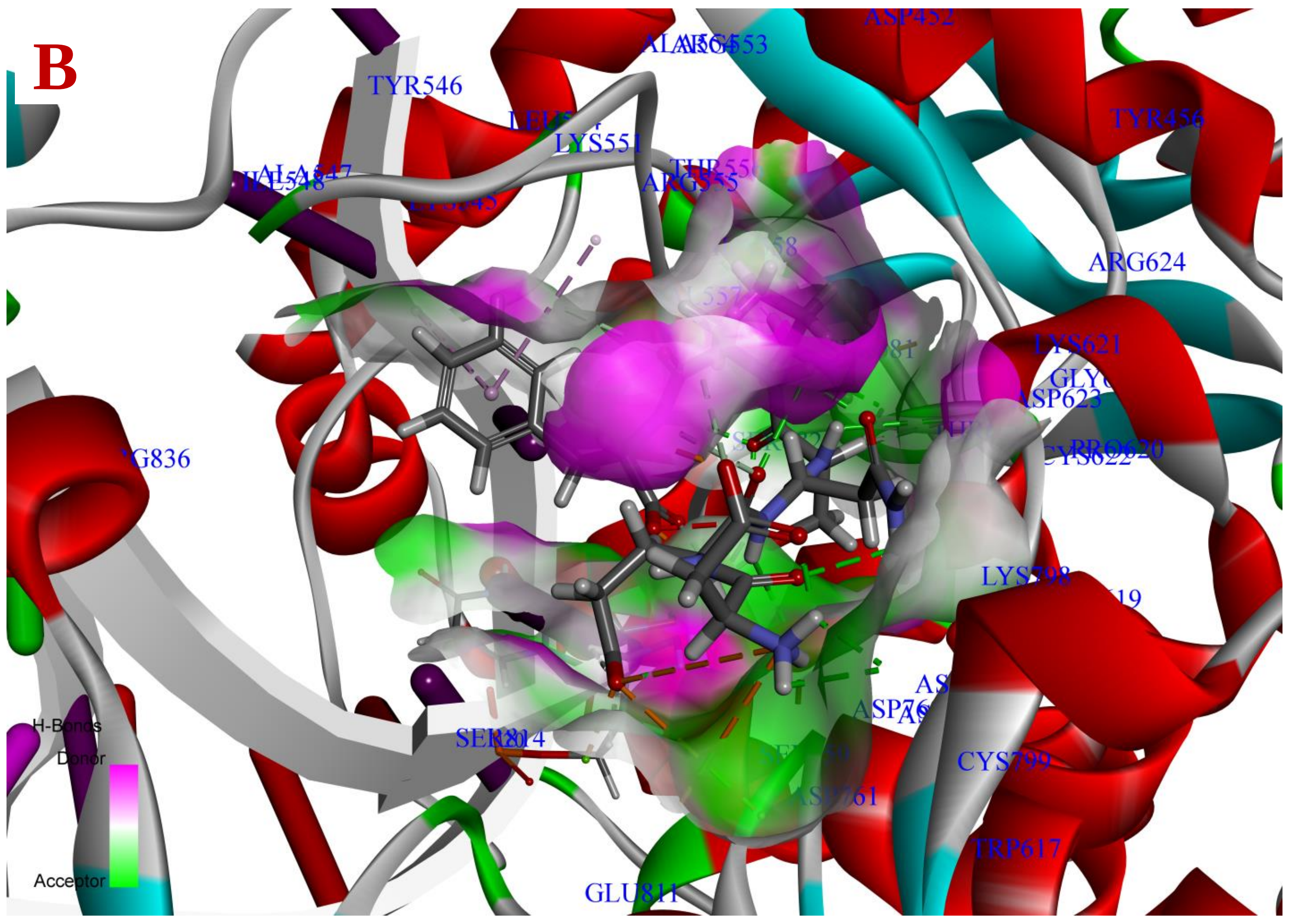

C

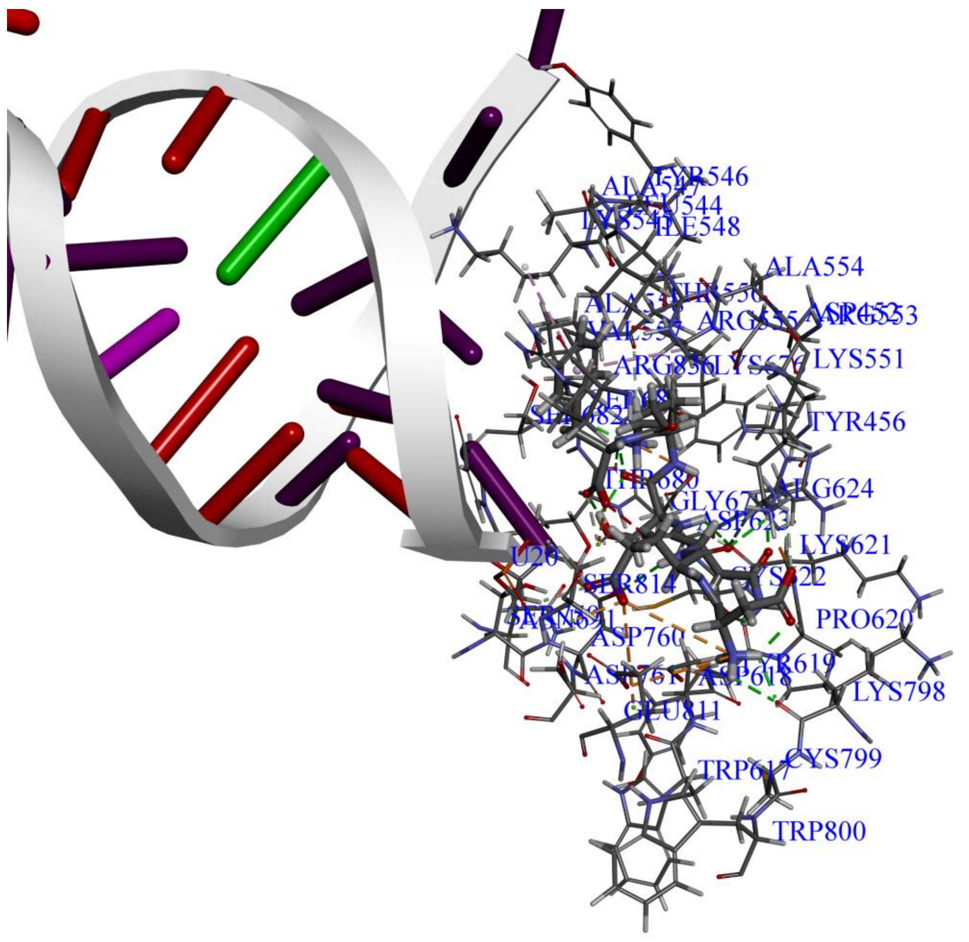

D

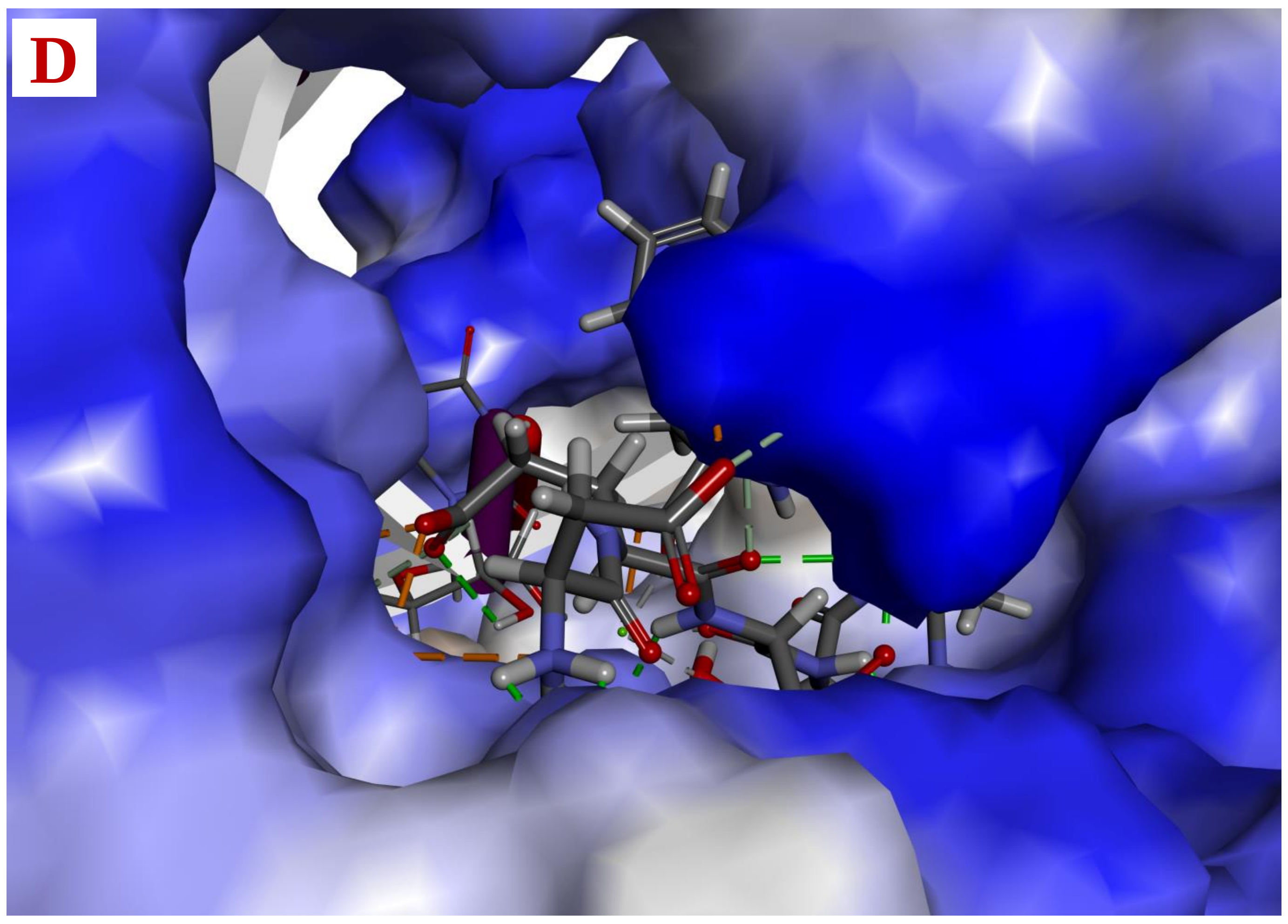

Molecule	Total Interaction Energy(kcal/mol)
PR1	-971.6133

E

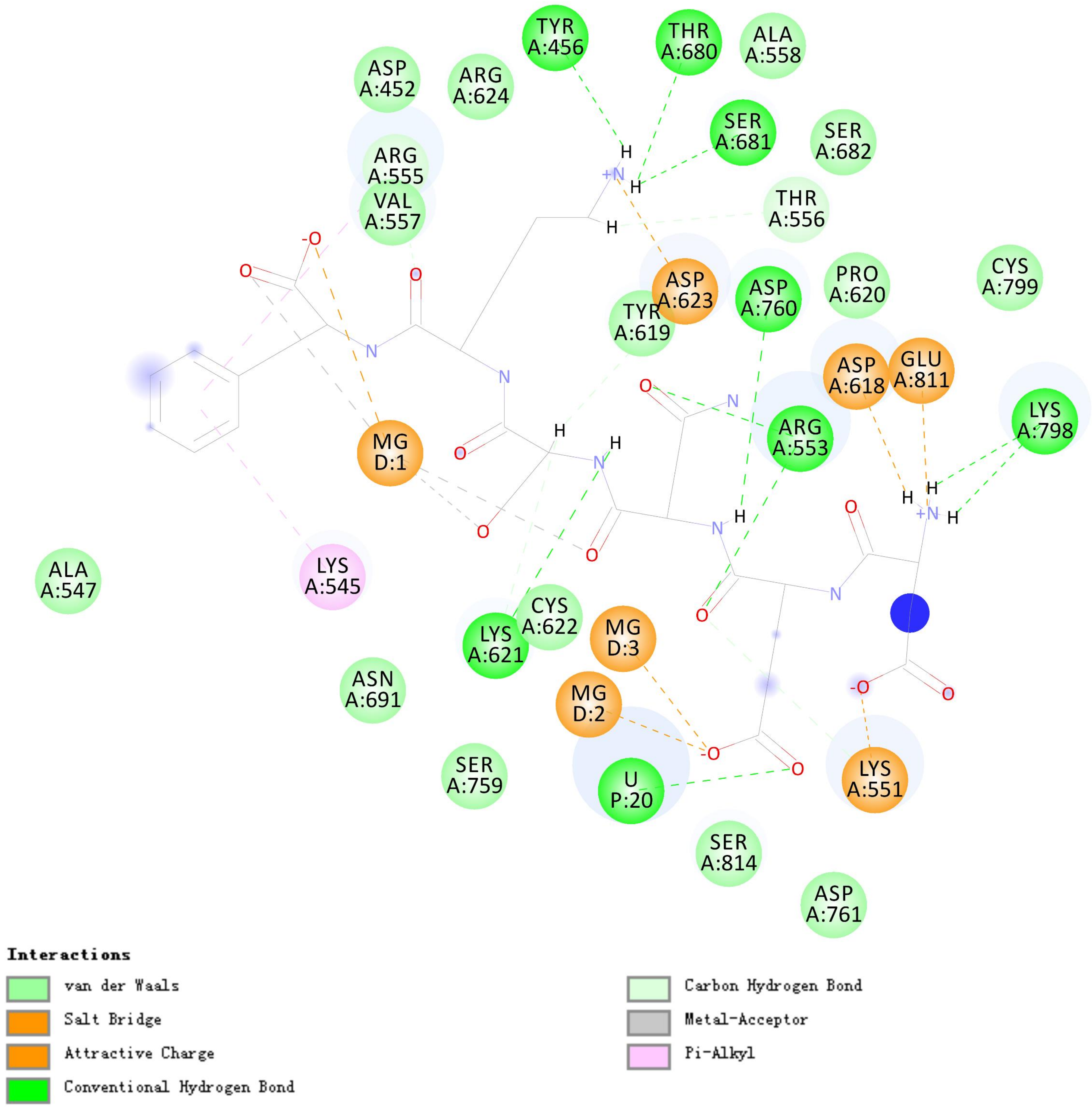

Supplemental Figure S15

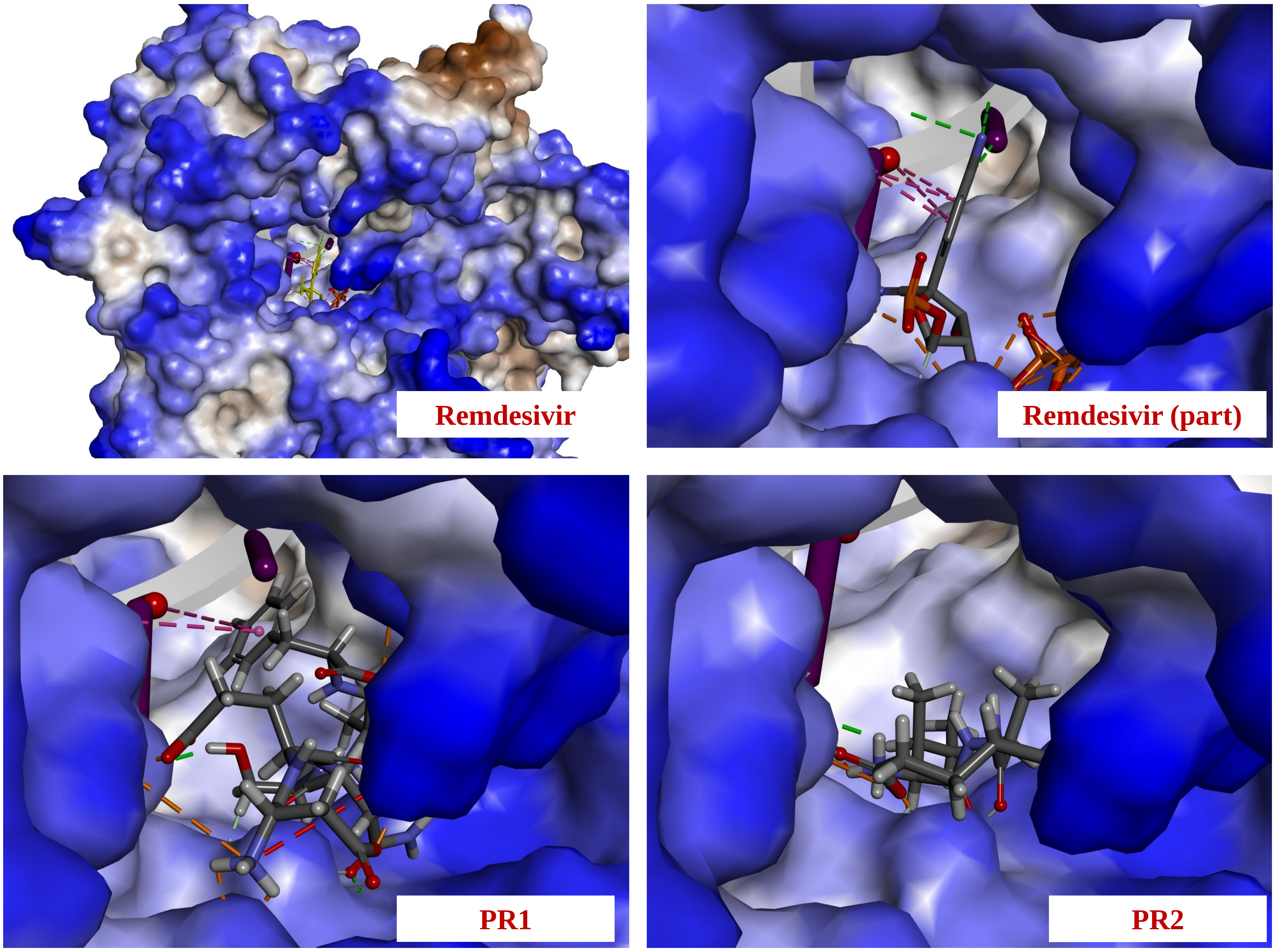

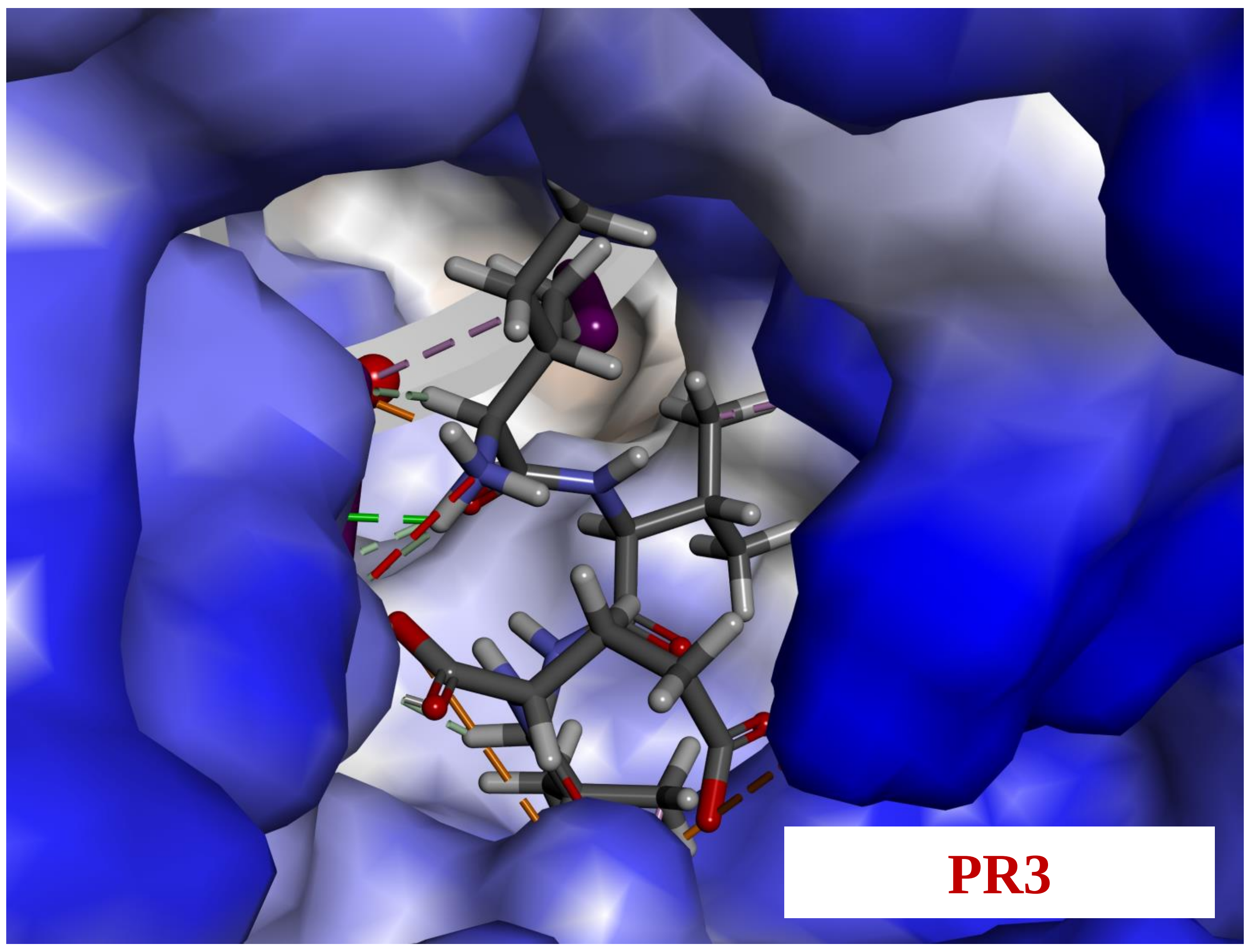

Molecule	Total Interaction Energy (Non-covalent, kcal/mol)
Remdesivir (RMP)	-223.13061
Remdesivir (POP)	-1447.24517
PR1	-870.28747
PR2	-737.55523
PR3	-613.53135
